# HDAC7/c-Myc signaling pathway promotes the proliferation and metastasis of choroidal melanoma cells

**DOI:** 10.1038/s41419-022-05522-0

**Published:** 2023-01-18

**Authors:** Yimeng Zhang, Peng Ding, Yuanyong Wang, Changjian Shao, Kai Guo, Hanyi Yang, Yingtong Feng, Jiayi Ning, Minghong Pan, Ping Wang, Xiaolong Yan, Zhiqiang Ma, Jing Han

**Affiliations:** 1 Department of Ophthalmology, Tangdu Hospital, The Air Force Medical University, Xi’an, 710038 China; 2 Xi’an Medical University, Xi’an, 710086 China; 3 Department of Thoracic Surgery, Tangdu Hospital, The Air Force Medical University, Xi’an, 710038 China; 4 Department of Thoracic Surgery, Shaanxi Provincial People’s Hospital, The Third Affiliated Hospital of Xi’an Jiaotong University, Xi’an, 710068 China; 5 Department of Cardiothoracic Surgery, The Affiliated Huaihai Hospital of Xuzhou Medical University/The 71th Group Army Hospital of PLA, 236 Tongshan Road, Xuzhou, 221004 China; 6 Department of Medical Oncology, Senior Department of Oncology, Chinese PLA General Hospital, The Fifth Medical Center, Beijing, 100853 China

**Keywords:** Eye cancer, Cell migration

## Abstract

Choroidal melanoma (CM) is the most common type of diagnosed uveal melanoma (UM), which is prone to metastasis and exhibits a poor prognosis. The molecular mechanisms underlying CM progression need further elucidation to research effective therapeutic strategies. Histone deacetylase 7 (HDAC7) is very important in regulating cancer progression, but the significance and effect of HDAC7 on CM progression are unclear. In the present study, we found that HDAC7 is overexpressed in CM tissues versus normal tissues. We built HDAC7 overexpressing CM cell lines to study the functions of HDAC7 in CM progression and verified that upregulation of HDAC7 promoted the proliferation and metastasis of CM cells, while pharmacological inhibition of HDAC7 suppressed both the proliferation and metastasis of CM cells. Furthermore, we found that the aforementioned cancer-promoting effect of HDAC7 was mediated by c-Myc. Targeted inhibition of c-Myc inhibited CM progression by interfering with the HDAC7/c-Myc signaling pathway. Our study highlighted the function of targeting the HDAC7/c-Myc signaling pathway to intervene in the pathological process of CM, which provides potential therapeutic strategies for CM treatment.

## Introduction

Uveal melanoma (UM) is a kind of common primary intraocular malignancy in adults. Choroidal melanoma (CM) accounts for 90% of UM cases and always occurs in patients between the ages of 50 and 70 years [1]. A previous study reported a higher incidence of CM in Caucasian patients. In terms of sex, male patients account for a slightly higher proportion of CM patients [2]. Despite treatment, up to 50% of patients with CM will die from metastases, especially liver metastases, within 10 years of diagnosis. There are currently no effective treatment methods for patients with metastatic melanoma, and the prognosis remains extremely poor [3]. Therefore, novel therapeutic targets and approaches are desperately needed to improve the clinical efficacy of treatments and the prognosis of CM patients.

As a member of the histone deacetylase (HDAC) IIa subfamily, HDAC7 plays an indispensable role in regulating multiple transcription and corepressor factors by deacetylating both histone and nonhistone proteins [4]. A previous study reported that abnormal HDAC7 expression levels are correlated with cell proliferation, metastasis, and angiogenesis in different cancers, such as colorectal cancer, glioma, and gastric cancer [5–7]. High HDAC7 is also linked with advanced cancers and leads to a poor prognosis, which has been validated in breast and lung cancers [8, 9]. However, the significance of HDAC7 and its effect on CM progression have not yet been elucidated.

As a proto-oncogene, *c-Myc* is crucial in modulating cell proliferation via both transcriptional activation and repression [10, 11]. It has been validated that c-Myc is crucial for regulating the long-term proliferation of cancer cells. Abnormal expression of c-Myc contributes to a poorly differentiated phenotype, tumor progression, and a poor prognosis [12]. c-Myc inhibition can cause tumor regression in transgenic mouse models [13]. Some scholars found that silencing HDAC7 can suppress the expression of c-Myc and further block cell cycle progression in Hela and MCF-7 cells, which demonstrates the close relationship between HDAC7 and c-Myc in tumor progression. However, it remains unclear whether the potential HDAC7/c-Myc signaling pathway is involved in CM regulation.

The results of this paper indicate that HDAC7 is significantly overexpressed in CM tissues. Furthermore, We generated HDAC7 overexpressing CM cell lines to explore the functions of HDAC7 in CM progression and verified that upregulation of HDAC7 promoted the proliferation and metastasis of CM cells, while pharmacological inhibition of HDAC7 suppressed both the proliferation and metastasis of CM cells. In addition, we found that the aforementioned cancer-promoting effect of HDAC7 was mediated by c-Myc. Targeted inhibition of c-Myc inhibited CM progression by interfering with the HDAC7/c-Myc signaling pathway. Our study highlighted the function of targeting the HDAC7/c-Myc signaling pathway to intervene in the process of CM, which provides potential therapeutic strategies for CM treatment.

## Methods

### Cell culture and lentivirus infection

Human CM cell lines (OCM1 and C918) were obtained from Hunan Fenghui Biotechnology Co., Ltd. (Changsha, China) or Shanghai Fuxiang Biological Technology Co., Ltd. (Shanghai, China) and cultured in DMEM (Gibco, NY, USA) supplemented with 10% fetal bovine serum (FBS, Gibco, NY, USA) and penicillin–streptomycin solution (100 units/ml) (Solarbio, Beijing, China). HDAC7 and empty vector lentiviruses were obtained from GeneChem (Shanghai, China). OCM1 and C918 cells were infected with lentiviruses according to the protocol provided by GeneChem Corporation.

### CM tissue samples and tissue microarray immunohistochemistry (IHC)

We first collected the data of CM patients who underwent surgery in Tangdu Hospital from March 2012 to October 2021 and screened 16 eligible patients as the research objects (Supplementary Table 1). The author submitted the report to the hospital ethics committee and obtained the approval of the Department (TDLL-202207-09). Before the experiment, full communication and exchange were carried out with subjects, and all subjects signed written informed consent. The subjects included in the study had no other diseases and had not received radiotherapy or chemotherapy before. After the intervention, follow-up will be carried out for a certain period of time, and the relevant data or information will be updated until January 2022. After the subjects were determined, the collected samples were prepared into paraffin-embedded tissue chips. Then IHC staining was performed on the collected samples with the prepared anti-HDAC7 (1:100, #33,418, CST) primary antibody according to the established process [14]. The immunostaining intensity classification included 0 (negative), 1 (weak), 2 (moderate), or 3 (strong), and the proportion classification of positively stained cells included 0 (<5%), 1 (6–25%), 2 (26–50%), 3 (51–75%), or 4 (>75%). The two numbers were multiplied to determine the total score. According to the average score, CM samples were divided into a low HDAC7 expression group and the high HDAC7 expression group.

### Colony formation assay

A total of 800 OCM1 or C918 cells were cultured in 6-well plates for 14 days. Then, colonies were fixed with formalin (Solarbio, Beijing, China). The colonies containing 50 cells were counted for further analysis.

### 5-Ethynyl-2′-deoxyuridine (EdU) incorporation assay

The BeyoClick EdU Cell kit with Alexa Fluor 594 (Beyotime, Shanghai, China) was used to detect the newly synthesized DNA and cell proliferation according to the manufacturer’s directions. Cells were imaged by an Olympus FV1000 confocal microscope (Olympus, Tokyo, Japan). EdU-positive cells were manually counted as percentages of cells calculated by the Hoechst 33342 nuclear label.

### Wound healing assay

Prepare the medium containing fetal bovine serum, and then inoculate the cells into its 6-well plate in strict accordance with the established process. When the cells reach 90–100% fusion, then use the prepared 200 μL pipette tip to produce scratches. The samples prepared by the above steps were washed three times with 1× phosphate-buffered saline, and after the impurities were completely removed, a new culture medium was added, and then photos were taken and stored. One day later, observe the gap between the edges of the wound, record, and save the results.

### Transwell cell migration assay

In the detection of cell migration and diffusion, the classical transwell detection method is introduced. First, the cells were incubated in a serum-free medium (SFM) for one day. After completion, inoculate in the upper chamber (Costar, Corning, NY, USA). The second step is to fill 200 μL SFM in the upper chamber according to the established process, and the lower chamber was filled with the same medium supplemented with 10% FBS. The third step is to fix the cells with formalin after 24 h, then stained them with 0.1% crystal violet, and finally record and save the results.

### Western blot

Western blotting method was used in the following experiment [14, 15], loading 25 μg of total protein lysate per lane. The following primary antibodies were used: anti-HDAC7 (1:1000, #33,418, CST), anti-c-Myc (1:1000, #5605, CST), anti-CDK1 (1:1000, 19,532-1-AP, Proteintech), anti-Cyclin B1 (1:1000, 55,004-1-AP, Proteintech), anti-CDK2 (1:1000, 10,122-1-AP, Proteintech), anti-CyclinA2 (1:1000, 18,202-1-AP, Proteintech), anti-E-cadherin (1:1000,#14,472, CST), anti-Zeb1 (1:1000, 21,544-1-AP, Proteintech), anti-α-SMA (1:1000, 14,395-1-AP, Proteintech) and anti-β-actin (1:1000, #3700, CST). The secondary antibodies used were HRP-linked anti-IgG antibodies (1:5000, Zhongshan Company, Beijing, China).

### Cell treatment

SAHA, TMP269, and 10058-F4 (MedChemExpress, USA) stock solutions were prepared in DMSO media prior to the subsequent experiments. The CM cells were treated with these chemical compounds for 48 h and then harvested for further analyses.

### In vivo tumor xenograft models

The animal experiment was authorized by the Animal Experimentation Ethics Committee of The Air Force Medical University (No. IACUC-20210557). Male athymic nude mice (6-8 weeks) were purchased from formal institutions. Different groups (LV-Control/LV-HDAC7) of 7 × 10^6^ C918 cells were separately injected into the left/right flanks of nude mice. Then days after injection, tumors were palpable and an equal number of mice from LV-Control/LV-HDAC7 groups were randomly selected to be treated with 50 mg/kg/d SAHA intragastrically. After 21–28 days, the mice were all sacrificed at the same time, and the tumors were excised and weighed for further analysis.

### Statistical analyses

In this study, the experimental data are input into SPSS 23.0 software for statistics and processing. In addition, this paper also introduces χ^2^ test to further characterize the relationship between HDAC7 content and pathological characteristics of CM patients. When comparing between groups, the student t-test is introduced. The data collected in the experiment is described by mean ± SD. Statistical significance was set at *P* < 0.05.

## Results

### HDAC7 overexpression promotes CM cell proliferation

**Fig. 1:**
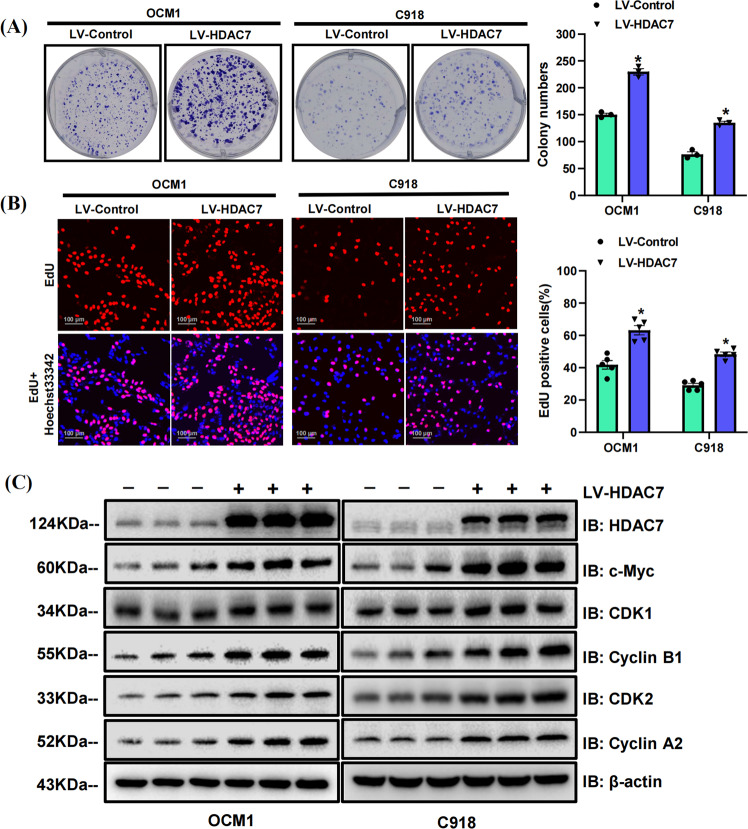
HDAC7 overexpression facilitates OCM1 and C918 cell proliferation in vitro. **A** Typical picture and statistical analysis of the colony formation assay in LV-HDAC7 OCM1/C918 and LV-Control OCM1/C918 cells. **B** Typical pictures and statistical analysis of the EdU incorporation assay in LV-HDAC7 OCM1/C918 and LV-Control OCM1/C918 cells. The results were calculated as the ratio of the number of EdU-positive cells (red fluorescence) to the total number of Hoechst 33342 stained cells (blue fluorescence). Scale bar, 100 μm (inset). **C** Typical Western blot results of HDAC7, c-Myc, CDK1, Cyclin B1, CDK2, and Cyclin A2 in LV-HDAC7 OCM1/C918 and LV-Control OCM1/C918 cells. β-actin was used as an internal control. Data are exhibited as the mean ± SD. Student’s *t*-test, *P < 0.05 vs. LV-Control group. Abbreviations: LV lentivirus, HDAC7 histone deacetylase 7.

To detect the expression of HDAC7 in CM, we conducted IHC staining analysis to investigate the HDAC7 level in a tissue microarray including 16 CM tumor-normal tissues, and it was significantly higher in CM tissues (*P* < 0.001, Fig S1A). In addition, CM patients with T3/T4 stage exhibited higher HDAC7 expression than those with T1/T2 stage (*P* < 0.01, Fig. S1B). To explore the function of HDAC7 in regulating the CM proliferative phenotype, stable HDAC7-overexpressing OCM1 and C918 cell lines were generated through HDAC7 lentivirus. We conducted colony formation and EdU incorporation assays to assess the role of HDAC7 in OCM1 and C918 cell proliferation in vitro. In contrast with the control group, we found that HDAC7 overexpression obviously facilitated colony formation and enhanced the number of EdU-positive cells in both OCM1 and C918 cells (*P* < 0.05, Fig. 1A, B). Western blot analysis showed that c-Myc, which plays an indispensable role in tumor progression, was dramatically upregulated in the HDAC7 overexpression groups (Fig. 1C). We also detected the expression level of some cell cycle proteins and found that CDK1, Cyclin B1, CDK2, and Cyclin A2 could be upregulated by HDAC7 overexpression while others exhibited no evident changes (Fig. 1C, Fig. S2A). To summarize, the results demonstrate that HDAC7 has a proliferation-promoting effect on CM cells.

### Pharmacological inhibition of HDAC7 suppresses the proliferative abilities of CM cells

**Fig. 2:**
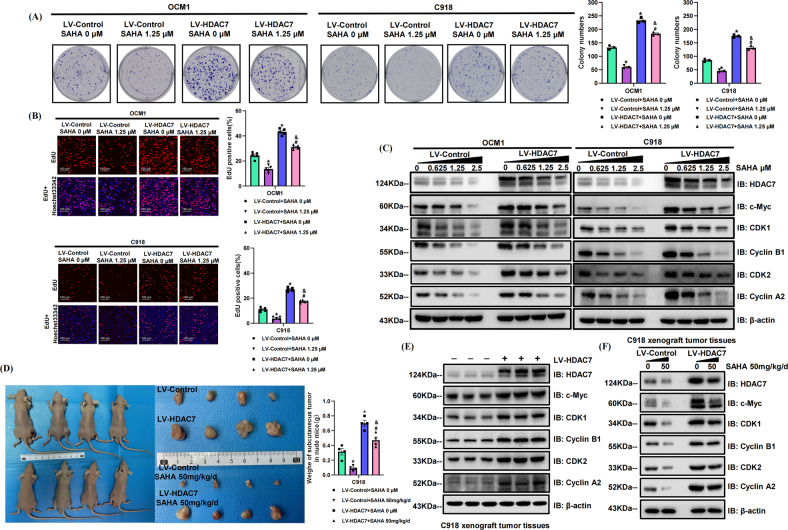
SAHA suppresses HDAC7-mediated CM cell proliferation both in vitro and in vivo. LV-Control and LV-HDAC7 OCM1/C918 cells were pretreated with SAHA for 48 h in vitro. Athymic nude mice were treated with 50 mg/kg/d SAHA intragastrically. **A** Typical pictures and statistical analysis of the colony formation assay. **B** Typical pictures and statistical analysis of the EdU incorporation assay in LV-HDAC7 OCM1/C918 and LV-Control OCM1/C918 cells. The results were calculated as the ratio of the number of EdU-positive cells (red fluorescence) to the total number of Hoechst 33342 stained cells (blue fluorescence). Scale bar, 100 μm (inset). **C** Typical Western blot results of HDAC7, c-Myc, CDK1, Cyclin B1, CDK2, and Cyclin A2 in LV-HDAC7 OCM1/C918 and LV-Control OCM1/C918 cells. **D** Representative pictures of tumor weight changes after subcutaneous injection of C918 cells. Photographs show tumor xenograft morphologies in the LV-Control and LV-HDAC7 C918 groups treated with/without SAHA. **E** Typical Western blot results of HDAC7, c-Myc, CDK1, Cyclin B1, CDK2, and Cyclin A2 in the LV-Control and LV-HDAC7 C918 xenograft tumor tissues. **F** Typical Western blot results of HDAC7, c-Myc, CDK1, Cyclin B1, CDK2, and Cyclin A2 in the LV-Control and LV-HDAC7 C918 xenograft tumor tissues treated with/without SAHA. β-actin was used as an internal control. Data are exhibited as the mean ± SD. Student’s *t*-test, in vitro*:* *P < 0.05 vs. the LV-Control + SAHA 0 μM group; ^#^*P* < 0.05 vs. the LV-Control + SAHA 1.25 μM group; ^&^*P* < 0.05 vs. the LV-HDAC7 + SAHA 0 μM group, in vivo*:* *P < 0.05 vs. the LV-Control + SAHA 0 mg/kg/d group; ^#^*P* < 0.05 vs. the LV-Control + SAHA 50 mg/kg/d group; ^&^*P* < 0.05 vs. the LV-HDAC7 + SAHA 0 mg/kg/d group. Abbreviations: LV lentivirus, HDAC7 histone deacetylase 7.

**Fig. 3:**
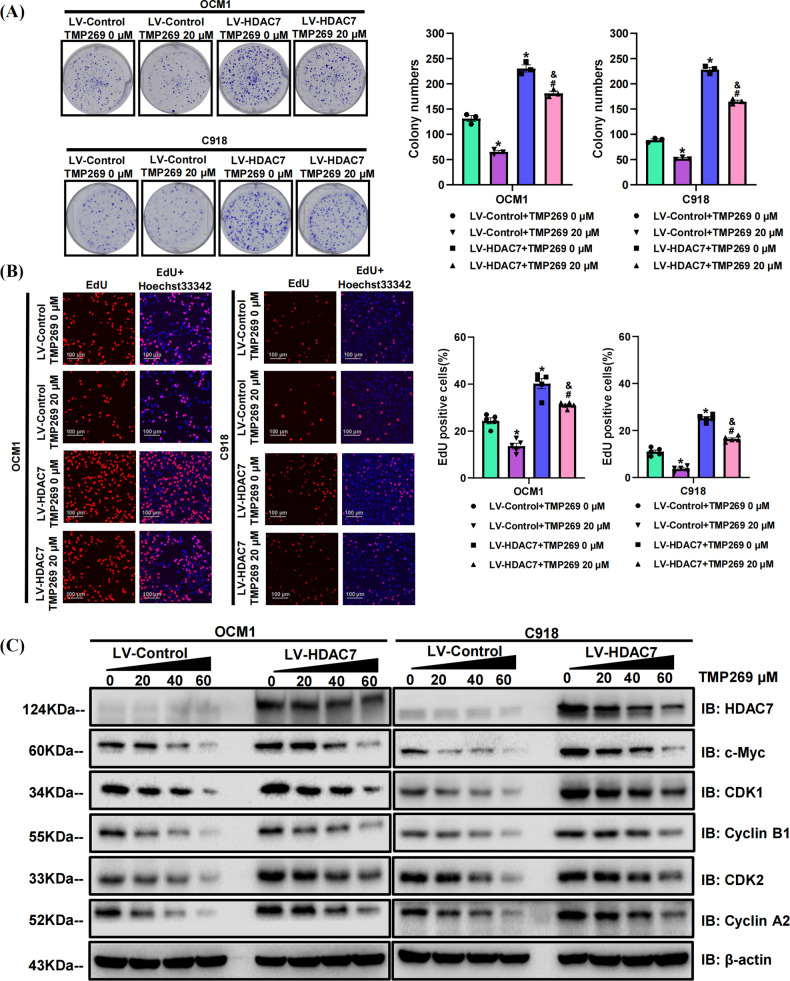
TMP269 suppresses HDAC7-mediated OCM1 and C918 cell proliferation. LV-Control and LV-HDAC7 OCM1/C918 cells were pretreated using TMP269 for 48 h. **A** Typical pictures and statistical analysis of the colony formation assay. **B** Typical pictures and statistical analysis of the EdU incorporation assay in LV-HDAC7 OCM1/C918 and LV-Control OCM1/C918 cells. The results were calculated as the ratio of the number of EdU-positive cells (red fluorescence) to the total number of Hoechst 33342 stained cells (blue fluorescence). Scale bar, 100 μm (inset). **C** Typical Western blot results of HDAC7, c-Myc, CDK1, Cyclin B1, CDK2, and Cyclin A2 in LV-HDAC7 OCM1/C918 and LV-Control OCM1/C918 cells. β-actin was used as an internal control. Data are exhibited as the mean ± SD. Student’s *t* test, *P < 0.05 vs. the LV-Control + TMP269 0 μM group; ^#^*P* < 0.05 vs. the LV-Control + TMP269 20 μM group; ^&^*P* < 0.05 vs. the LV-HDAC7 + TMP269 0 μM group. Abbreviations: LV lentivirus, HDAC7 histone deacetylase 7.

To further confirm the proliferation-promoting role of HDAC7 in CM, the HDAC7-overexpressing and control OCM1/C918 cell lines were treated with the pan-inhibitor vorinostat (SAHA) for 48 h. The HDAC7-induced promotion of proliferation was significantly attenuated by SAHA, which was confirmed by colony formation and EdU incorporation assays (Fig. 2A, B). Western blot results validated that increased SAHA concentrations contributed to decreased HDAC7 levels in both cell lines (Fig. 2C). Consistently, the upregulation in c-Myc, CDK1, Cyclin B1, CDK2, and Cyclin A2 level induced by HDAC7 overexpression were also inhibited by SAHA, and there is a positive correlation between the corresponding inhibition level and dose (Fig. 2C). The average expression levels of HDAC7, c-Myc, CDK1, Cyclin B1, CDK2, and Cyclin A2 in the HDAC7-overexpressing groups were higher than those in the normal cell groups (Fig. 2C). To further validate the proliferation-promoting role of HDAC7 in vivo, we established tumor xenograft nude mice models with LV-Control/LV-HDAC7 C918 cells and found that the weight of xenograft tumors in the HDAC7 overexpression group was evidently greater (*P* < 0.05, Fig. 2D). Western blot results exhibited increased expression of c-Myc, CDK1, Cyclin B1, CDK2, and Cyclin A2 in HDAC7 overexpressing xenograft tumors, which were consistent with the results of in vitro experiments (Fig. 2E). After treating with SAHA, the weight of xenograft tumors in the SAHA treatment group was evidently less than those in group without SAHA (*P* < 0.05, Fig. 2D). Western blotting also showed that after treatment with SAHA, the expression levels of HDAC7, c-Myc, CDK1, Cyclin B1, CDK2 and Cyclin A2 were downregulated in both the HDAC7-overexpressing groups and control groups (Fig. 2F). To further verify the above results, we repeated colony formation assay, EdU incorporation assay and Western blot with the specific class IIa inhibitor TMP269 and obtained the conclusions consistent with the above experimental results utilizing SAHA (Fig. 3A–C). Therefore, HDAC7-induced CM cell proliferation was partially reversed by HDAC7 inhibition.

**Fig. 4:**
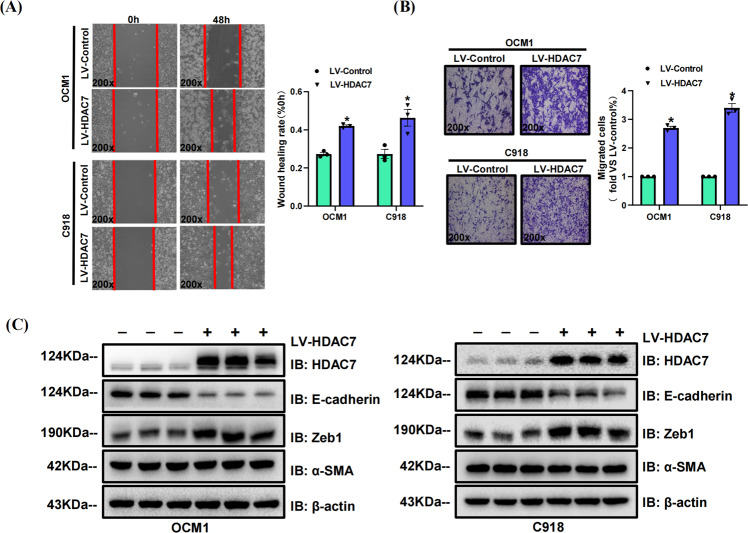
HDAC7 overexpression facilitates OCM1 and C918 cell metastasis in vitro. **A** Typical pictures (×200) and statistical analysis of the wound healing assay. In wound healing assays, the migratory capacity was shown as the mean scratch area. The initial scratch area (0 h) was set to 100%. **B** Typical pictures (×200) and statistical analysis of the transwell assay. **C** Typical Western blot results of HDAC7, E-cadherin, Zeb1, and α-SMA in LV-HDAC7 OCM1/C918 and LV-Control OCM1/C918 cells. β-actin was used as an internal control. Data are exhibited as the mean ± SD. Student’s *t*-test, *P < 0.05 vs. LV-Control group. Abbreviations: LV lentivirus, HDAC7 histone deacetylase 7.

### HDAC7 overexpression promotes CM cell metastasis

To further study the role of HDAC7 in CM progression, we assessed the metastatic changes of HDAC7-overexpressing and control OCM1/C918 cell lines in vitro. We conducted wound healing and transwell assays, validating that HDAC7 overexpression contributed to increased migration capacity in both OCM1 and C918 cells (Fig. 4A, B). In addition, we detected the level of some essential proteins associated with the EMT process using Western blotting and found that HDAC7 overexpression obviously downregulated E-cadherin expression but upregulated Zeb1. However, the level of another EMT biomarker, α-SMA, did not change (Fig. 4C). Therefore, the results above demonstrate that HDAC7 has a metastasis-promoting effect on CM cells.

**Fig. 5:**
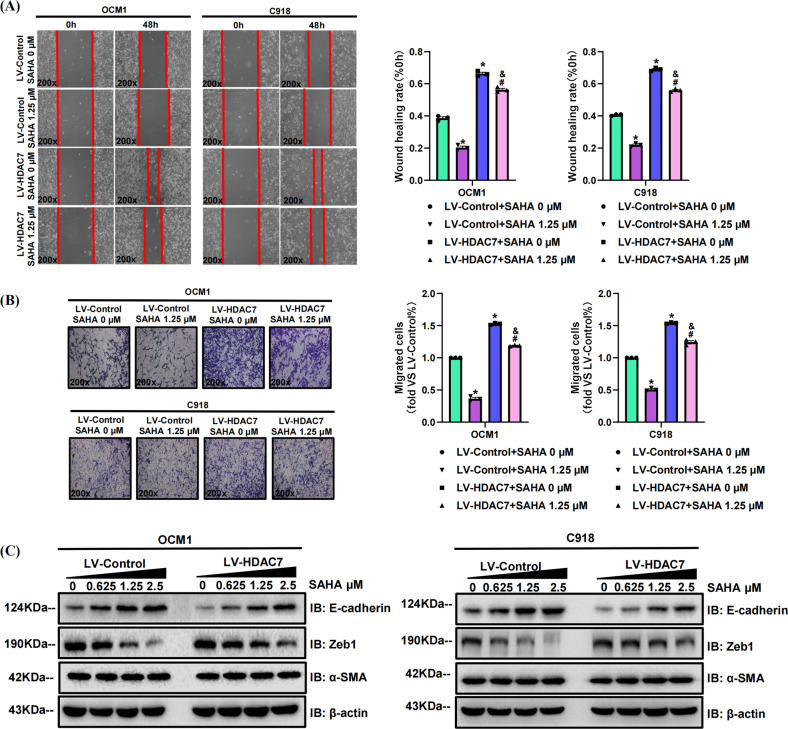
SAHA suppresses HDAC7-mediated OCM1 and C918 cell metastasis. LV-Control and LV-HDAC7 OCM1/C918 cells were pretreated with SAHA for 48 h. **A** Typical pictures (×200) and statistical analysis of the wound healing assay. In wound healing assays, the migratory capacity was shown as the mean scratch area. The initial scratch area (0 h) was set to 100%. **B** Typical pictures (×200) and statistical analysis of the transwell assay. **C** Typical Western blot results of HDAC7, E-cadherin, Zeb1, and α-SMA in LV-HDAC7 OCM1/C918 and LV-Control OCM1/C918 cells. β-actin was used as an internal control. Data are exhibited as the mean ± SD. Student’s *t*-test, *P < 0.05 vs. the LV-Control + SAHA 0 μM group; ^#^*P* < 0.05 vs. the LV-Control + SAHA 1.25 μM group; ^&^*P* < 0.05 vs. the LV-HDAC7 + SAHA 0 μM group. Abbreviations: LV lentivirus, HDAC7 histone deacetylase 7.

### Pharmacological inhibition of HDAC7 suppresses the metastatic abilities of CM cells

**Fig. 6:**
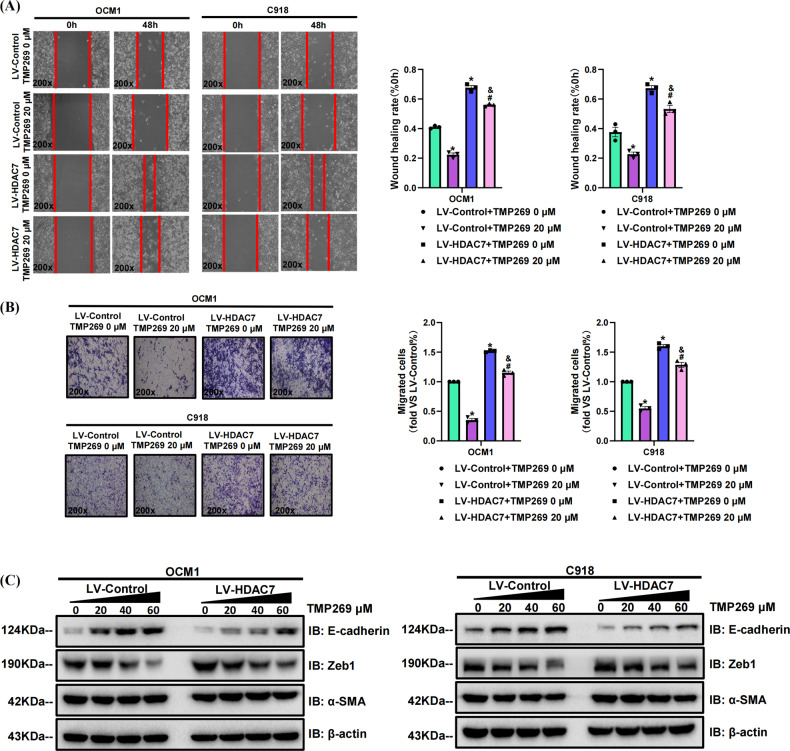
TMP269 suppresses HDAC7-mediated OCM1 and C918 cell metastasis. LV-Control and LV-HDAC7 OCM1/C918 cells were pretreated with TMP269 for 48 h. **A** Typical pictures (×200) and statistical analysis of the wound healing assay. In wound healing assays, the migratory capacity was shown as the mean scratch area. The initial scratch area (0 h) was set to 100%. **B** Typical pictures (×200) and statistical analysis of the transwell assay. **C** Typical Western blot results of HDAC7, E-cadherin, Zeb1, and α-SMA in LV-HDAC7 OCM1/C918 and LV-Control OCM1/C918 cells. β-actin was used as an internal control. Data are exhibited as the mean ± SD. Student’s *t-*test, *P < 0.05 vs. the LV-Control + TMP269 0 μM group; ^#^*P* < 0.05 vs. the LV-Control + TMP269 20 μM group; ^&^*P* < 0.05 vs. the LV-HDAC7 + TMP269 0 μM group. Abbreviations: LV lentivirus, HDAC7 histone deacetylase 7.

We also incubated the HDAC7-overexpressing and control OCM1/C918 cell lines with SAHA for 48 h to further confirm the metastasis-promoting role of HDAC7 in CM. We found that the HDAC7-induced promotion of metastasis was significantly attenuated by SAHA, which was confirmed by wound healing and transwell assays (Fig. 5A, B). Western blot results validated that increased SAHA concentrations contributed to decreased HDAC7 and Zeb1 levels in both cell lines, but upregulated E-cadherin levels (Fig. 5C). The average expression levels of HDAC7 and Zeb1 in the HDAC7-overexpressing groups were higher than those in the control groups, while the E-cadherin level was higher in the control groups (Fig. 5C). To further verify the above results, we repeated wound healing assay, transwell assay and Western blot with TMP269 and obtained the conclusions consistent with the above experimental results utilizing SAHA (Fig. 6A–C). Therefore, HDAC7-induced CM cell metastasis is partially reversed by HDAC7 inhibition.

### HDAC7 promotes the proliferation and metastasis of CM cells via c-Myc

**Fig. 7:**
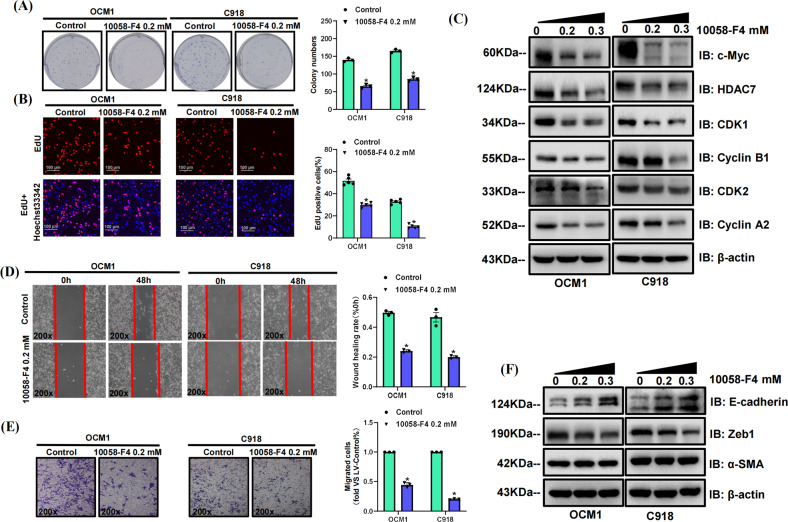
10058-F4 suppresses the HDAC7/c-Myc signaling pathway to inhibit OCM1 and C918 cell proliferation and metastasis. OCM1/C918 cells were pretreated with 10058-F4 for 48 h. **A** Typical pictures and statistical analysis of the colony formation assay. **B** Typical pictures and statistical analysis of the EdU incorporation assay in OCM1/C918 cells. The results were calculated as the ratio of the number of EdU-positive cells (red fluorescence) to the total number of Hoechst 33342 stained cells (blue fluorescence). Scale bar, 100 μm (inset).**C** Typical Western blot results of c-Myc, HDAC7, CDK1, Cyclin B1, CDK2, and Cyclin A2 in OCM1/C918 cells. **D** Typical pictures (×200) and statistical analysis of the wound healing assay. In wound healing assays, the migratory capacity was shown as the mean scratch area. The initial scratch area (0 h) was set to 100%. **E** Typical pictures (×200) and statistical analysis of the transwell assay. **F** Typical Western blot results of E-cadherin, Zeb1, and α-SMA in OCM1/C918 cells. β-actin was used as an internal control. Data are exhibited as the mean ± SD. Student’s *t*-test, *P < 0.05 vs. control group. Abbreviations: HDAC7 histone deacetylase 7.

A previous study reported that *c-Myc* gene amplification is linked to tumor progression and increased aggressiveness [16]. In our study, we found upregulated c-Myc expression level and proliferative capacity in HDAC7-overexpressing OCM1/C918 cell lines. Besides, silencing HDAC7 contributed to decreased c-Myc level, further demonstrating that c-Myc expression was modulated by HDAC7 (Fig. S2B, C). To explore the potential function of c-Myc in CM progression, we treated original OCM1 and C918 cell lines with the c-Myc-specific inhibitor 10058-F4 to evaluate the proliferative and metastatic capacity of CM cells. We conducted colony formation and EdU incorporation assays in 10058-F4-treated OCM1 and C918 cells, showing that c-Myc inhibition could significantly suppress CM cell proliferation (Fig. 7A, B). Western blot results showed that with increased concentrations of 10058-F4, the levels of both c-Myc and HDAC7 were decreased (Fig. 7C). Consistently, the levels of CDK1, Cyclin B1, CDK2, and Cyclin A2 were also decreased, which indicated that c-Myc inhibition might attenuate the proliferative capacity of CM cells (Fig. 7C). We also evaluated the role of c-Myc in metastasis. Both wound healing and transwell assays validated that c-Myc inhibition contributed to decreased migration capacity in both OCM1 and C918 cells (Fig. 7D-E). Western blot results showed that with increasing concentrations of 10058-F4, the E-cadherin expression level was increased, while the Zeb1 level was decreased. The α-SMA expression level still showed no change (Fig. 7F). Altogether, these results revealed that HDAC7 promotes the proliferation and metastasis of CM cells via c-Myc, thus facilitating CM progression.

### Targeted inhibition of c-Myc suppresses CM progression by interfering with the HDAC7/c-Myc signaling pathway

**Fig. 8:**
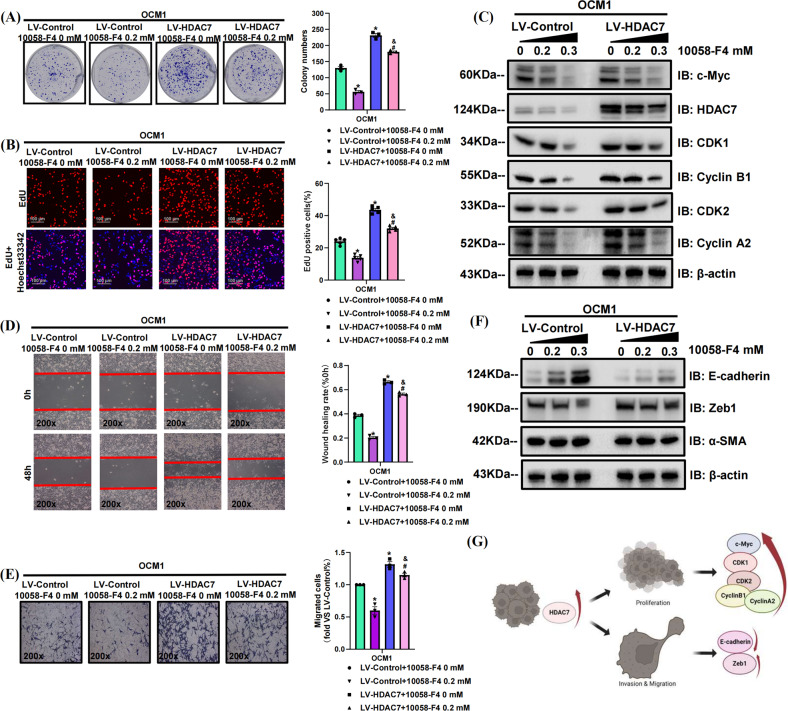
10058-F4 suppresses the HDAC7/c-Myc signaling pathway to inhibit the proliferation and metastasis of OCM1 cells. LV-Control and LV-HDAC7 OCM1 cell was pretreated with 10058-F4 for 48 h. **A** Typical pictures and statistical analysis of the colony formation assay. **B** Typical pictures and statistical analysis of the EdU incorporation assay in LV-HDAC7 and LV-Control OCM1 cell. The results were calculated as the ratio of the number of EdU-positive cells (red fluorescence) to the total number of Hoechst 33342 stained cells (blue fluorescence). Scale bar, 100 μm (inset).**C** Typical Western blot results of HDAC7, c-Myc, CDK1, Cyclin B1, CDK2, and Cyclin A2 in LV-HDAC7 and LV-Control OCM1 cells. **D** Typical pictures (×200) and statistical analysis of the wound healing assay. In wound healing assays, the migratory capacity was shown as the mean scratch area. The initial scratch area (0 h) was set to 100%. **E** Typical pictures (×200) and statistical analysis of the transwell assay. **F** Typical Western blot results of E-cadherin, Zeb1, and α-SMA in LV-HDAC7 and LV-Control OCM1 cell. **G** Schematic picture of the molecular mechanism by which HDAC7 facilitates CM progression. HDAC7/c-Myc signaling pathway upregulates the level of CDK1, Cyclin B1, CDK2, Cyclin A2, and Zeb1 and decreases E-cadherin levels, thereby facilitating CM proliferation and metastasis. β-actin was used as an internal control. Data are exhibited as the mean ± SD. Student’s *t* test, *P < 0.05 vs. the LV-Control + 10058-F4 0 mM group; ^#^*P* < 0.05 vs. the LV-Control + 10058-F4 0.2 mM group; ^&^*P* < 0.05 vs. the LV-HDAC7 + 10058-F4 0 mM group. Abbreviations: LV lentivirus, HDAC7, histone deacetylase 7.

To further validate the role of the HDAC7/c-Myc signaling pathway in CM progression, we also incubated the HDAC7-overexpressing and control OCM1 cell line with 10058-F4 for 48 h. In proliferation-related assays, the HDAC7-induced promotion of proliferation was significantly attenuated by 10058-F4, which was confirmed by both colony formation and EdU incorporation assays (Fig. 8A, B). Western blot results showed that increased 10058-F4 concentrations contributed to decreased HDAC7 and c-Myc levels (Fig. 8C). Consistently, the changes in CDK1, Cyclin B1, CDK2, and Cyclin A2 expression induced by HDAC7 overexpression were also inhibited by 10058-F4 and there is a positive correlation between the corresponding inhibition level and dose (Fig. 8C). The average expression levels of HDAC7, c-Myc, CDK1, Cyclin B1, CDK2, and Cyclin A2 in the HDAC7-overexpressing groups were higher than those in the normal cell groups (Fig. 8C). In metastasis-related assays, the HDAC7-induced promotion of metastasis was significantly attenuated by 10058-F4, which was confirmed by both wound healing and transwell assays (Fig. 8D, E). Western blot results validated that increased 10058-F4 concentration contributed to decreased Zeb1 level, while the level of E-cadherin was upregulated (Fig. 8F). The average expression of Zeb1 in the HDAC7-overexpressing groups was higher, while the E-cadherin level was higher in the control groups (Fig. 8F). In addition, there has no obvious difference in the α-SMA expression level between the HDAC7-overexpressing and control groups or among the different drug concentration groups (Fig. 8F). Altogether, these results revealed that the effects of the HDAC7/c-Myc signaling pathway in promoting CM cell proliferation and metastasis can be partially reversed by c-Myc inhibition.

## Discussion

CM is the most common type of UM and is prone to metastasis and exhibits poor prognosis [1]. At present, the mechanism of CM progression has not been fully clarified, so more in-depth research and discussion are needed in order to effectively control the disease. The results of this study showed that HDAC7 is significantly overexpressed in CM tissues and high AJCC T stage CM. HDAC7 promoted both the proliferation and metastasis of CM cells. In addition, our preliminary results indicated that CM progression could be enhanced via the HDAC7/c-Myc signaling pathway, thus providing a potential therapeutic target for future CM treatment.

HDAC7 plays an indispensable role in the modulation of various cellular activities, mainly including migration, proliferation, and apoptosis [16]. Dysregulated HDAC7 expression has been validated in various cancers, such as ovarian cancer, and glioma. HDAC7 overexpression contributes to migration and is linked with a poor prognosis in lung cancer, nasopharyngeal carcinoma, and esophageal squamous cell carcinoma (ESCC) [17–19]. In the present study, the IHC analysis results based on 16 paired CM tumors and normal samples showed that the HDAC7 level was significantly higher in CM tumor tissues. These results confirmed the carcinogenic function of HDAC7 in CM.

Previous research has reported that HDAC7 plays an indispensable pro-proliferative role in some cancer cells, including MCF-7, HeLa, and HCT116 [20, 21]. A recent study on lung cancer also validated HDAC7 contributed to the proliferation of cancer cells by deacetylating β-catenin and further activating FGF18 expression [18]. In our study, we explore the effect of HDAC7 on CM proliferation both in vivo and in vitro. The results showed that HDAC7 overexpression could enhance the proliferative ability of CM cells, which was validated by colony formation and EdU incorporation assays. Additionally, pharmacological inhibition of HDAC7 consistently suppressed CM cell proliferation. Western blot analysis indicated that HDAC7 overexpression upregulated c-Myc and some cell cycle regulatory molecules, further modulating the cell proliferative process. Consistently, pharmacologic inhibition of HDAC7 downregulated the molecules described above.

Studies have also reported that HDAC7 is crucial in cancer metastasis, for instance, in breast cancer, nasopharyngeal cancer, and ovarian cancer [17, 22]. A previous study verified that HDAC7 dramatically increases Snail levels and decreases E-cadherin levels, further promoting the migration and invasion of ESCC [19]. Our current study revealed that HDAC7 overexpression promotes the metastasis of CM cells, which was validated by wound healing and transwell assays. Pharmacological inhibition of HDAC7 also consistently suppressed CM cell metastasis. Western blot analysis showed that HDAC7 overexpression upregulated Zeb1 while downregulated E-cadherin, which suggested that CM metastasis might be facilitated by HDAC7-mediated changes in the expression of specific EMT biomarkers.

As an important transcription factor, c-Myc correlates with tumor progression and contributes to poor clinical outcomes in the context of multiple tumors [23]. Recent studies have shown that HDAC7 and c-Myc have a close relationship in promoting tumor aggression. Studies reveal that the carcinogenic effect of HDAC7 depends on the amplification of c-Myc, which promotes tumor cell escape from the cellular senescence process and facilitates tumor cell growth by suppressing the expression of p21/p27, thereby accelerating the G1-S cell cycle transition [20, 24, 25]. A recent study reported that HDAC7/β-catenin/c-Myc could form a positive feedback loop to enhance tumor cell growth in ESCC [26]. Besides, HDAC7 inactivation results in the inhibition of breast cancer stem cell phenotype by downregulating c-Myc [4]. HDAC7 silencing also attenuates c-Myc expression in MCF-7 and Hela cells, thereby blocking cell cycle progression [20]. In our study, we found that HDAC7 overexpression promoted CM progression and c-Myc expression while HDAC7 silencing caused decreased c-Myc level. Pharmacological inhibition of HDAC7 also consistently downregulated c-Myc levels. Surprisingly, c-Myc inhibition also decreased the expression of HDAC7 and partially attenuated HDAC7 overexpression-induced CM cell proliferation and metastasis. These results further validated that HDAC7 and c-Myc formed a signaling pathway to enhance CM progression. Targeted inhibition of c-Myc suppresses CM progression by interfering with the HDAC7/c-Myc signaling pathway.

Despite the findings in our study, there are some limitations that should be noted. The mechanism by which HDAC7 regulates c-Myc has not been fully elucidated. Previous studies indicated that β-catenin was essential in the HDAC7/c-Myc regulatory pathway. As the key mediator of the Wnt/β-catenin signaling, β-catenin is associated with various diseases including cancer [27]. Ma et al. validated that HDAC7 deacetylated β-catenin and promoted its nuclear import, further upregulating c-Myc expression and promoting ESCC tumor growth [26]. Guo et al. also reported that HDAC7 facilitated non-small cell lung cancer proliferation and metastasis via β-catenin/FGF18 pathway [18]. Therefore, it is inferred that β-catenin may play an indispensable role in the HDAC7/c-Myc signaling pathway during CM progression. Relevant conclusions need to be clarified by further experiments. In addition to the limitation mentioned above, we focused on the role and mechanism of HDAC7, and the upstream signaling pathway of HDAC7 has not been thoroughly explored. Besides, only one kind of HDAC7-overexpressing CM cell line was selected for both in vivo and c-Myc pharmacological inhibition experiments (not both the OCM1 and C918 cell lines). Our further experiments aim to answer the above questions and increase the reliability of our study.

## Conclusion

Overall, our studies first confirmed that HDAC7 functioned as a tumor promoter by enhancing CM cell proliferation and metastasis. We demonstrated that HDAC7 is overexpressed in CM tissues compared with normal tissues. More importantly, CM progression could be enhanced via the HDAC7/c-Myc signaling pathway, thus providing new ideas for future CM treatment.

## Supplementary information

Supplementary figure legends

Figure S1

Figure S2

Supplementary Table 1

Original Western Blots

Original Data File

Checklist

## Data Availability

The datasets are available from the corresponding author upon reasonable request.
