# Supplementary material for: HDAC7/c-Myc signaling pathway promotes the proliferation and metastasis of choroidal melanoma cells
Source: Cell Death Dis. 2023 Jan 18;14(1):38. doi: 10.1038/s41419-022-05522-0 (PMC9849404; doi:10.1038/s41419-022-05522-0)
Supplement: Supplementary file 1 — Supplementary figure legends [file 41419_2022_5522_MOESM1_ESM.docx]

**Figure S1** Correlation of high HDAC7 expression levels with tumor/nontumor tissues and low/high AJCC tumor size. (A) Typical IHC images exhibiting HDAC7 level in CM tissue and adjacent non-tumor tissue. (B) The levels of HDAC7 in 16 CM patients were statistically analyzed based on the IHC results of tissue microarray. Abbreviations: HDAC7, histone deacetylase 7.

**Figure S2** (A) Typical Western blot results of CDK4, CDK6, Cyclin D1, Cyclin D3 and Cyclin E1 in LV-HDAC7 and LV-control OCM1/C918 cells. (B) Typical Western blot results of HDAC7 in LV-shHDAC7 #1, #2 and LV-control OCM1/C918 cells. (C) Typical Western blot results of HDAC7 and c-Myc in LV-shHDAC7 and LV-control OCM1/C918 cells. β-actin was used as an internal control. Abbreviations: LV, lentivirus; HDAC7, histone deacetylase 7.
