# Supplementary figures and images for: HDAC7/c-Myc signaling pathway promotes the proliferation and metastasis of choroidal melanoma cells
Source: Cell Death Dis. 2023 Jan 18;14(1):38. doi: 10.1038/s41419-022-05522-0 (PMC9849404; doi:10.1038/s41419-022-05522-0)

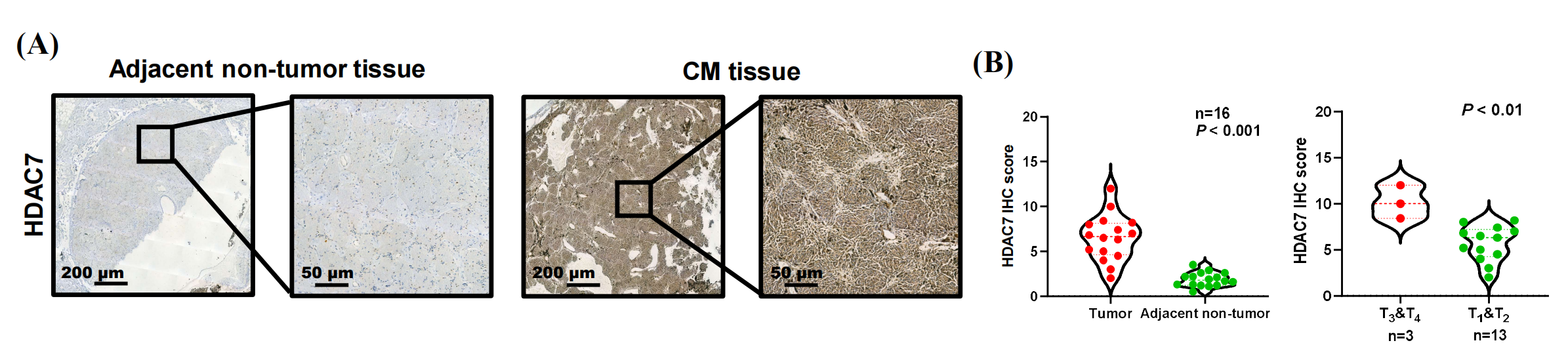

Supplement: Supplementary file 2 — Figure S1 [file 41419_2022_5522_MOESM2_ESM.tif]

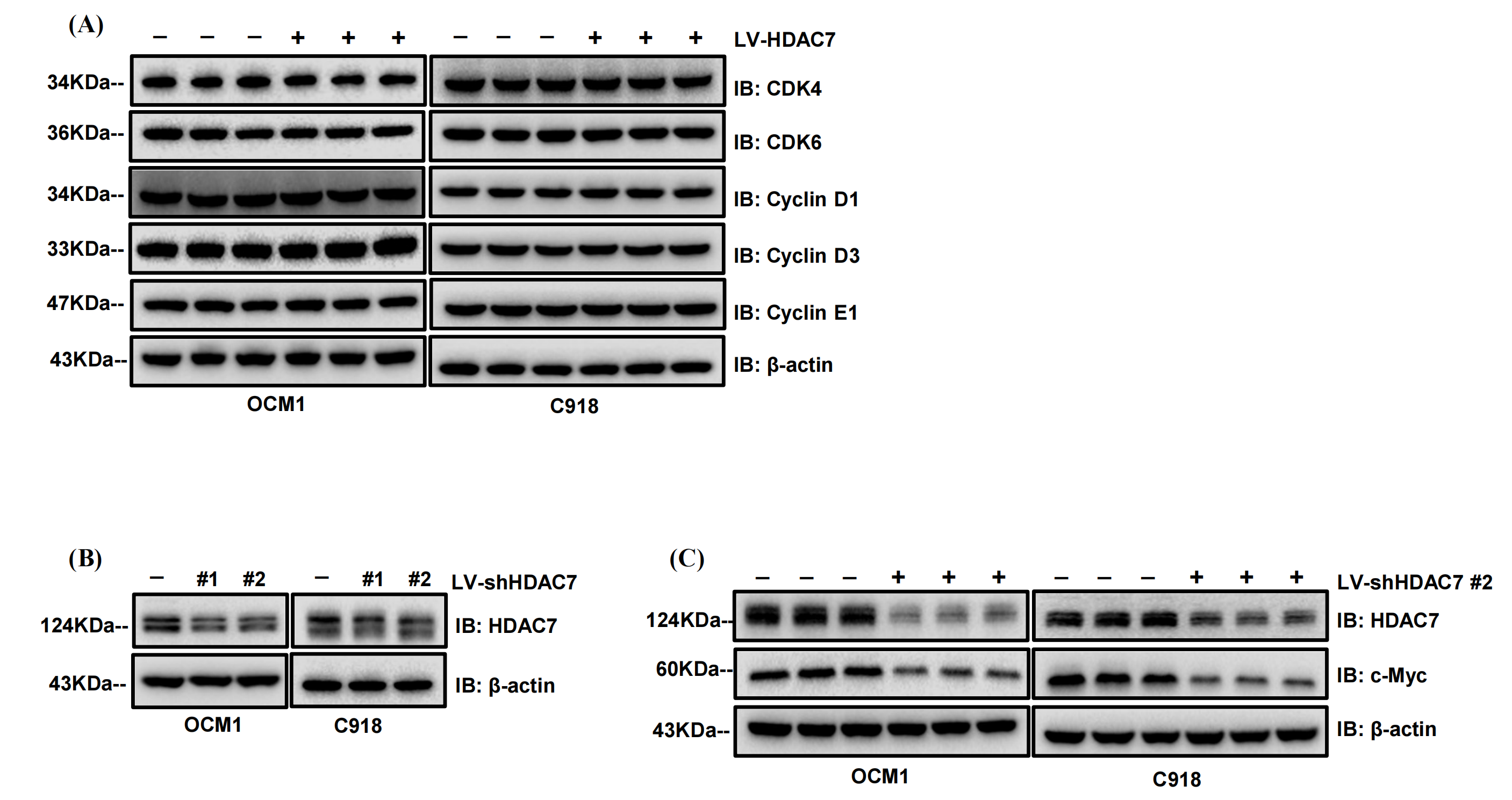

Supplement: Supplementary file 3 — Figure S2 [file 41419_2022_5522_MOESM3_ESM.tif]
