# Supplementary material for: HDAC7/c-Myc signaling pathway promotes the proliferation and metastasis of choroidal melanoma cells
Source: Cell Death Dis. 2023 Jan 18;14(1):38. doi: 10.1038/s41419-022-05522-0 (PMC9849404; doi:10.1038/s41419-022-05522-0)
Supplement: Supplementary file 4 — Supplementary Table 1 [file 41419_2022_5522_MOESM4_ESM.docx]

**Supplementary Table 1.** **Correlation of HDAC7 expression with the clinicopathological characteristics of patients with CM**

| **Clinicopathological variables** | **N** | **HDAC7 expression** | | |
| --- | --- | --- | --- | --- |
|  |  | **Low** | **High** | ***P*-value** |
| **Age** |  |  |  | 0.696 |
| <50 | 7 | 3 | 4 |  |
| ≥50 | 9 | 3 | 6 |  |
| **Gender** |  |  |  | 0.424 |
| Female | 6 | 3 | 3 |  |
| Male | 10 | 3 | 7 |  |
| **Tumor size** |  |  |  | 0.411 |
| <10 mm | 12 | 4 | 8 |  |
| 10-16 mm | 3 | 2 | 1 |  |
| >16 mm | 1 | 0 | 1 |  |
| **Tumor height** |  |  |  | 0.889 |
| <2.5 mm | 11 | 4 | 7 |  |
| 2.5-10 mm | 5 | 2 | 3 |  |
| >10 mm | 0 | 0 | 0 |  |
| **Ciliary body involvement** |  |  |  | 0.424 |
| Yes | 1 | 0 | 1 |  |
| No | 15 | 6 | 9 |  |
| **extraocular extension** |  |  |  | 0.696 |
| Yes | 2 | 1 | 1 |  |
| No | 14 | 5 | 9 |  |
| **TNM stages** |  |  |  | 0.869 |
| I–II | 13 | 5 | 8 |  |
| III–IV | 3 | 1 | 2 |  |
| **Distant Metastasis** |  |  |  | 0.242 |
| M0 | 14 | 6 | 8 |  |
| M1 | 2 | 0 | 2 |  |
