# Supplementary material for: HDAC7/c-Myc signaling pathway promotes the proliferation and metastasis of choroidal melanoma cells
Source: Cell Death Dis. 2023 Jan 18;14(1):38. doi: 10.1038/s41419-022-05522-0 (PMC9849404; doi:10.1038/s41419-022-05522-0)
Supplement: Supplementary file 5 — Original Western Blots [file 41419_2022_5522_MOESM5_ESM.pdf]

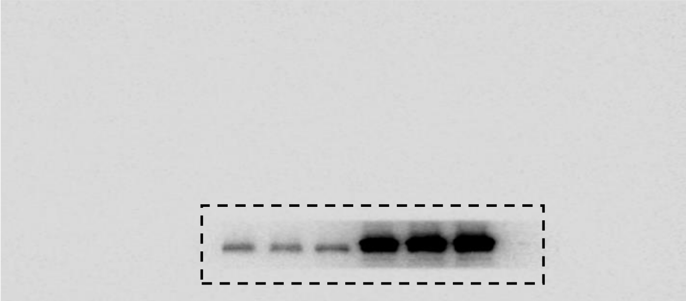
FIG1.C-OCM1-HDAC7

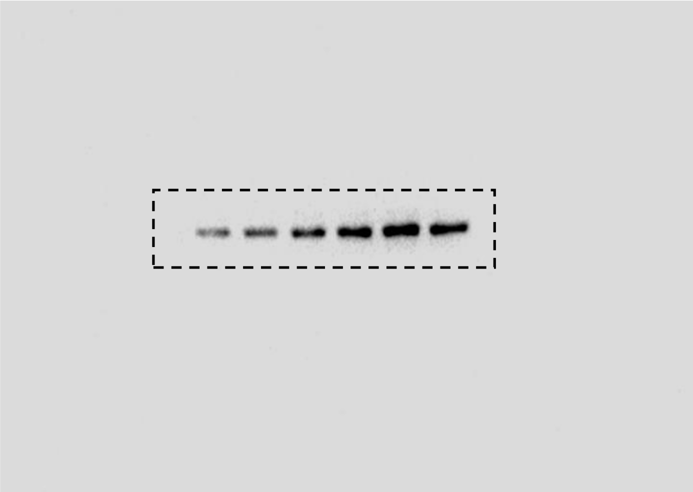
FIG1.C-OCM1- c-Myc

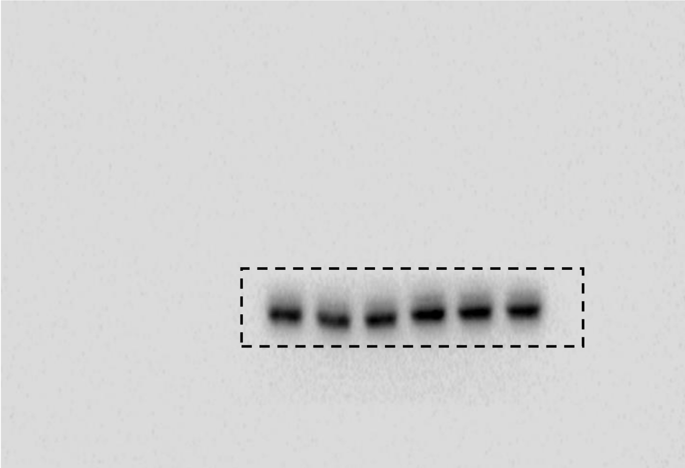
FIG1.C-OCM1-CDK1

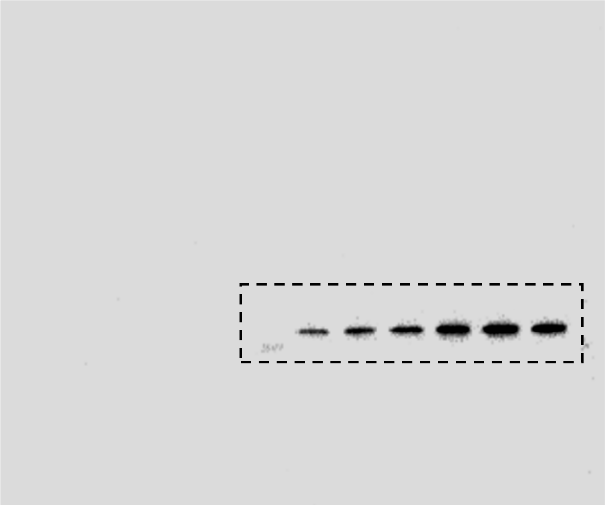
FIG1.C-OCM1- CyclinB1

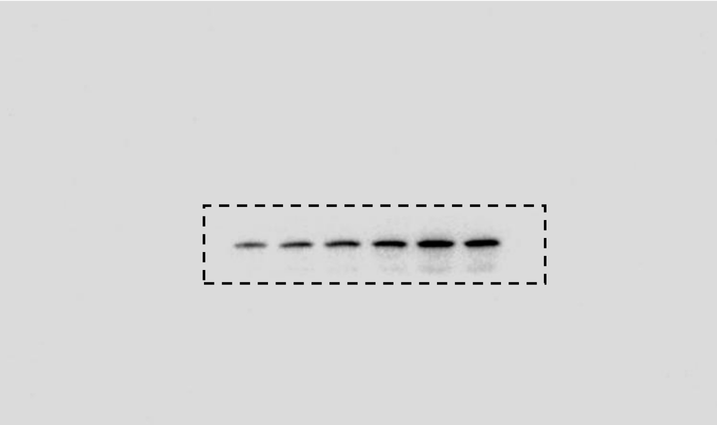
FIG1.C-OCM1-CDK2

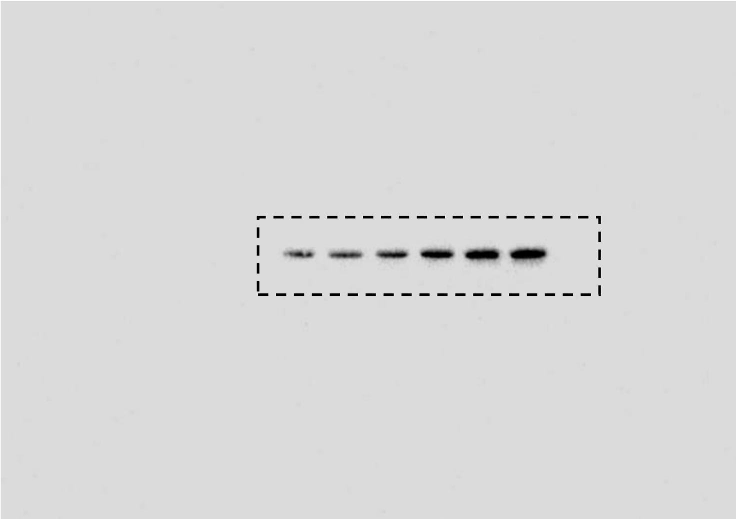
FIG1.C-OCM1- CyclinA2

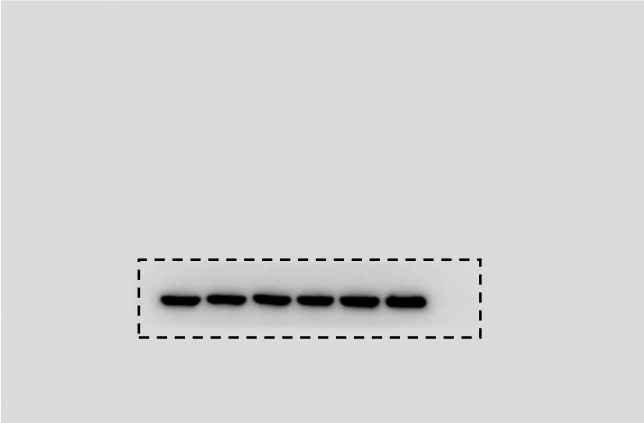
FIG1.C-OCM1- β-actin

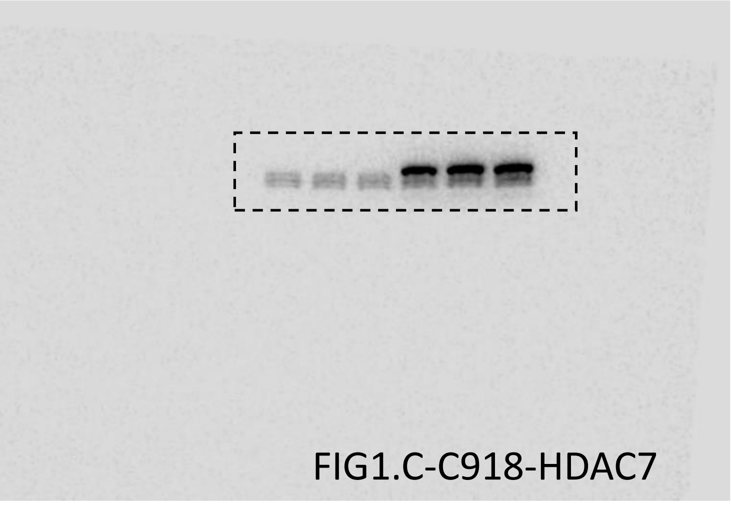
FIG1.C-C918-HDAC7

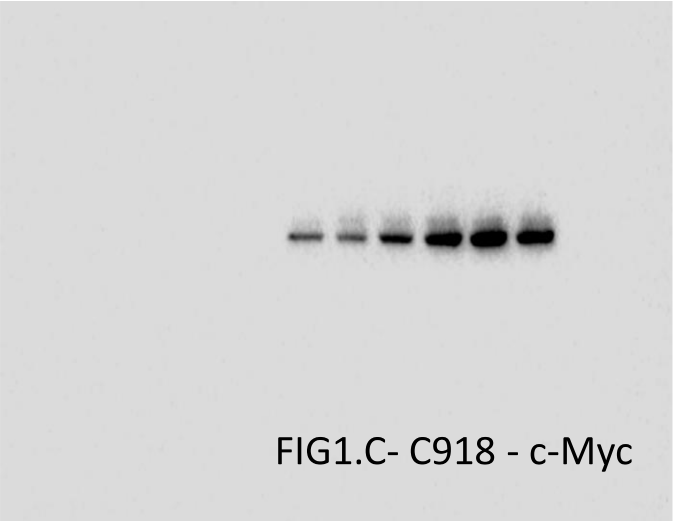
FIG1.C- C918 - c-Myc

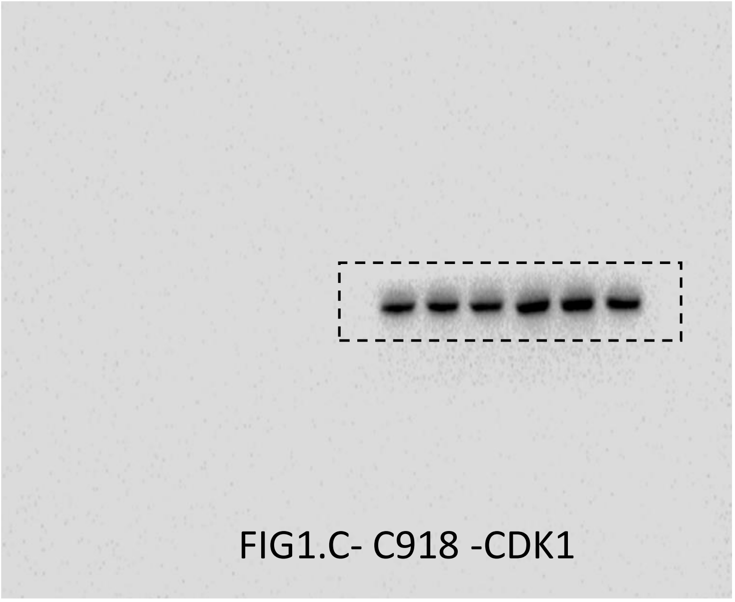
FIG1.C- C918 -CDK1

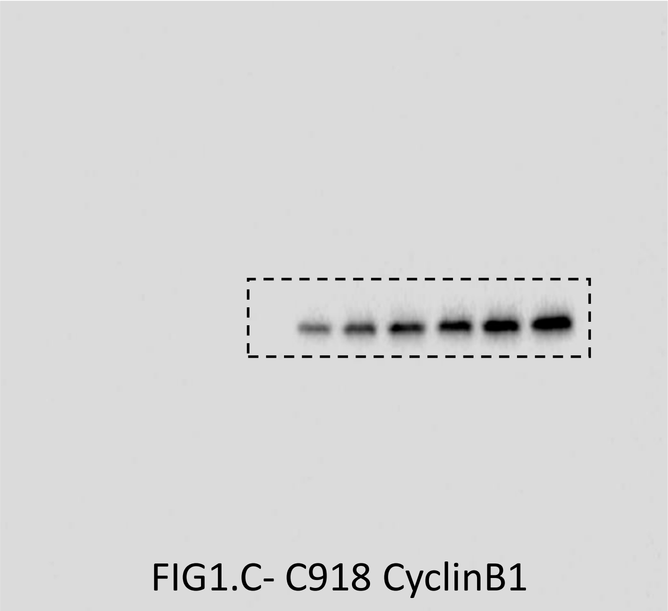
FIG1.C- C918 CyclinB1

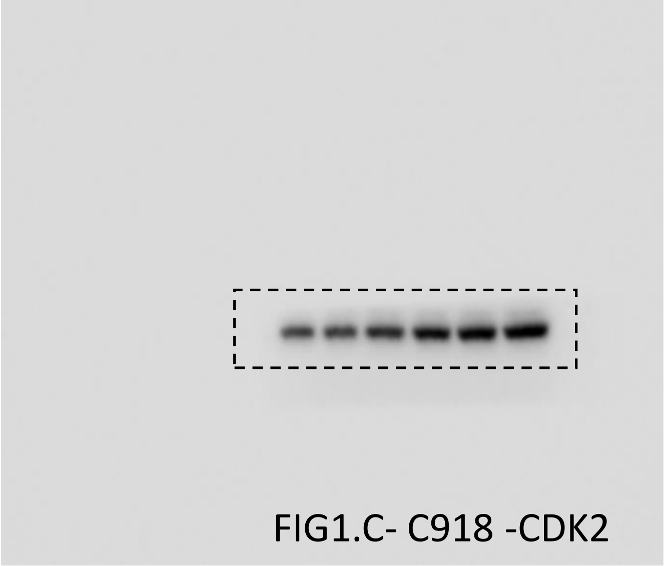
FIG1.C- C918 -CDK2

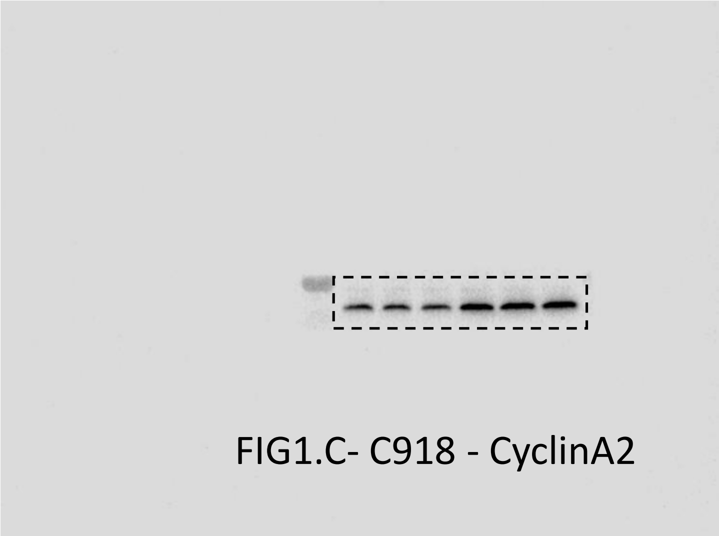
FIG1.C- C918 - CyclinA2

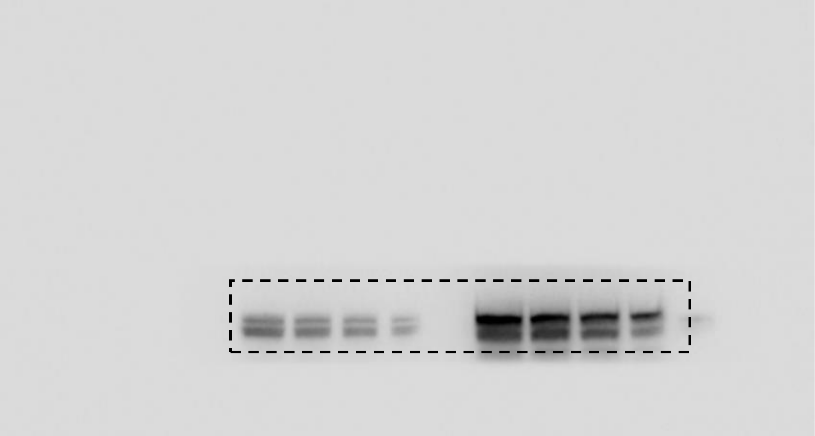

FIG2.C-OCM1-HDAC7

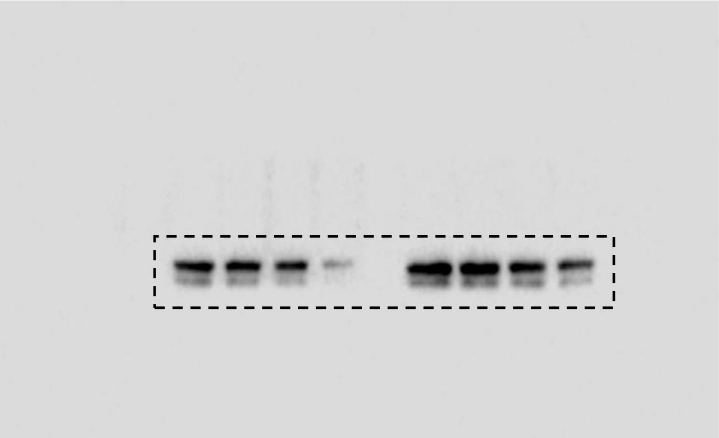

FIG2.C-OCM1- c-Myc

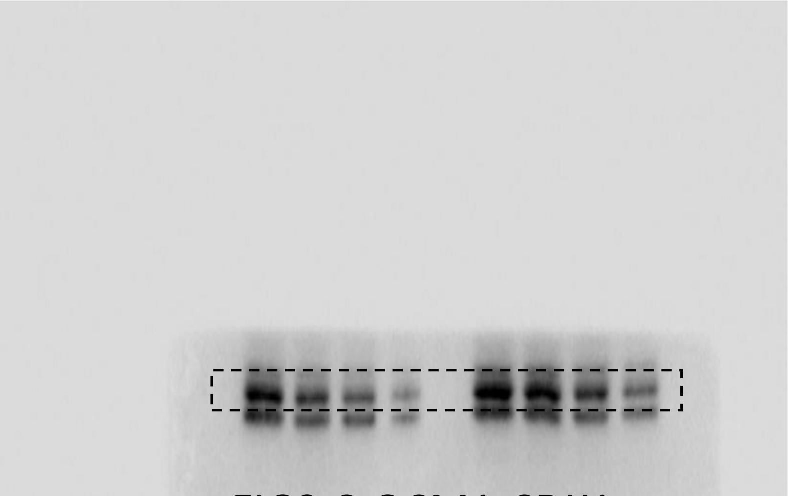

FIG2.C-OCM1-CDK1

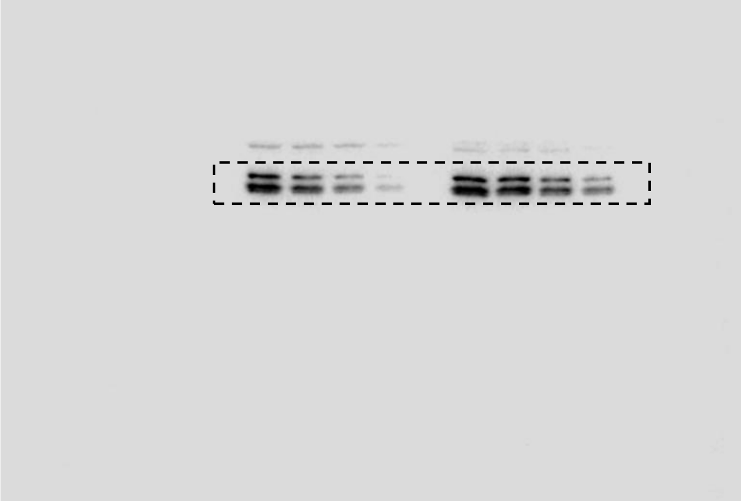

FIG2.C-OCM1- CyclinB1

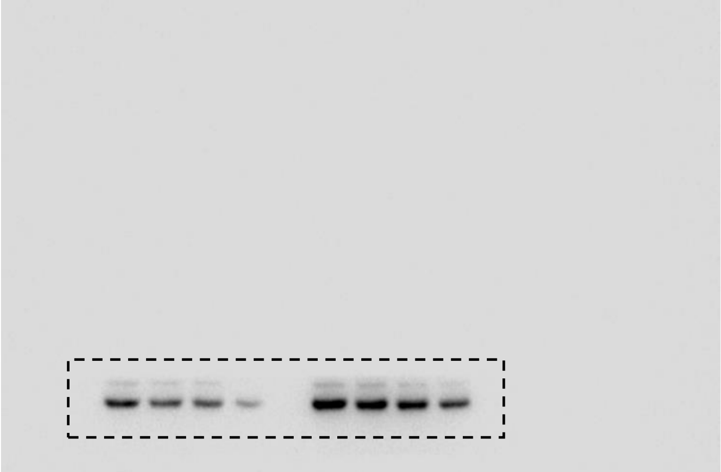

FIG2.C-OCM1- CDK2

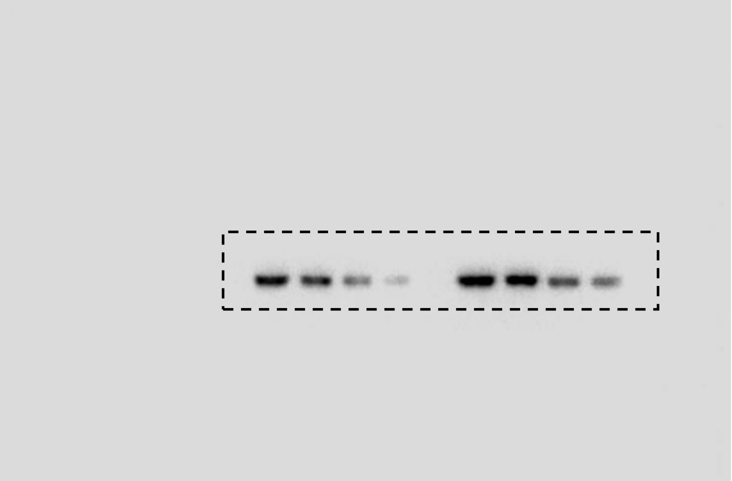

FIG2.C-OCM1- CyclinA2

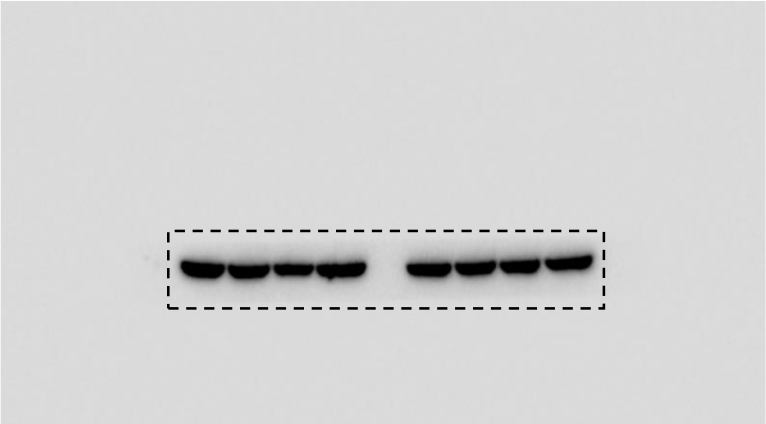

FIG2.C-OCM1- β-actin

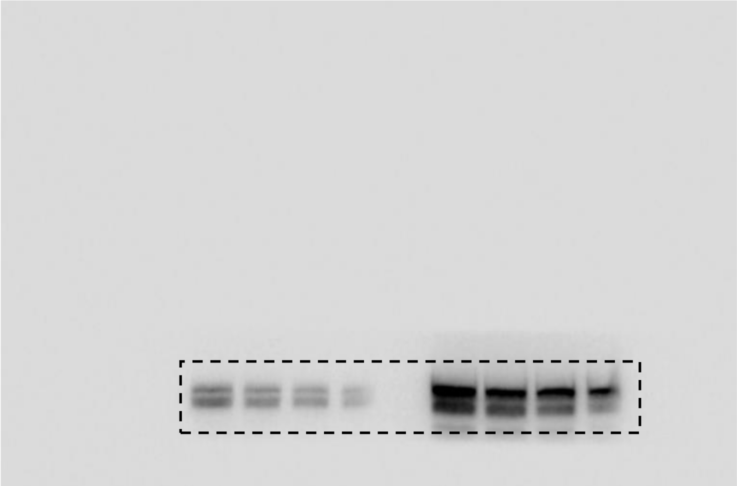

FIG2.C- C918 –HDAC7

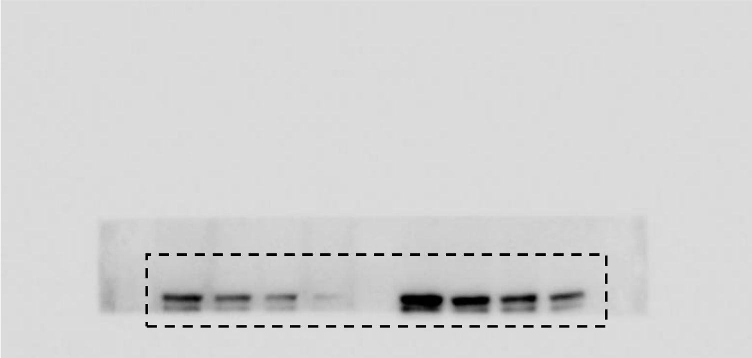

FIG2.C-C918- c-Myc

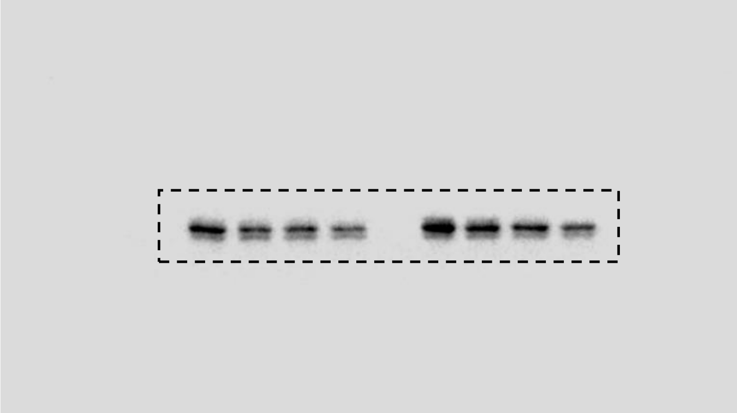

FIG2.C- C918 -CDK1

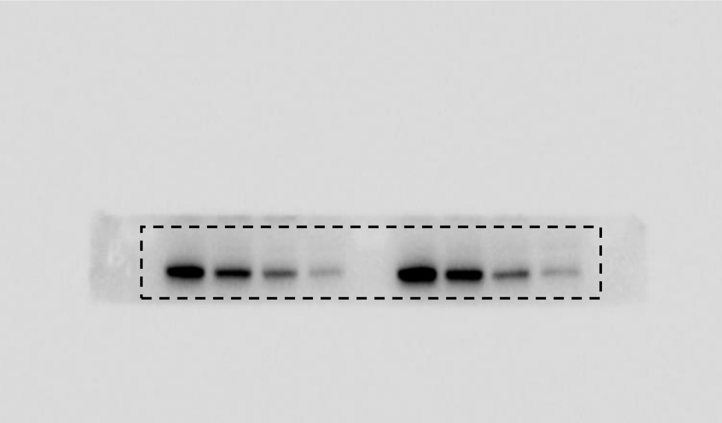

FIG2.C- C918 - CyclinB1

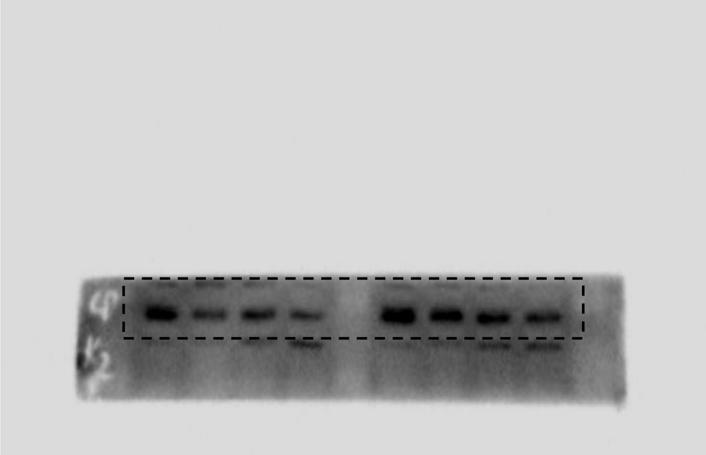

FIG2.C- C918 -CDK2

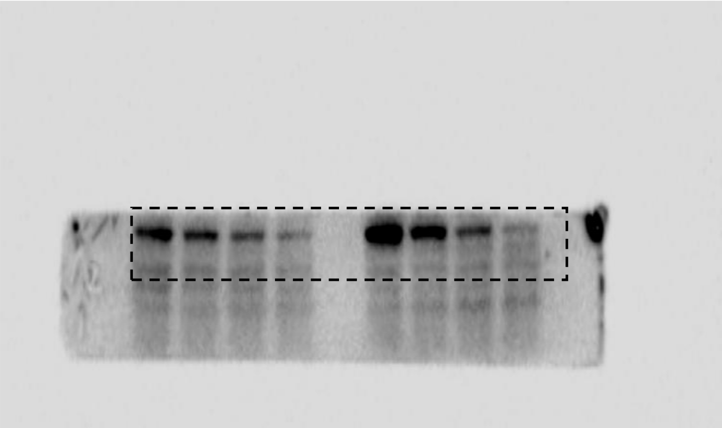

FIG2.C- C918 - CyclinA2

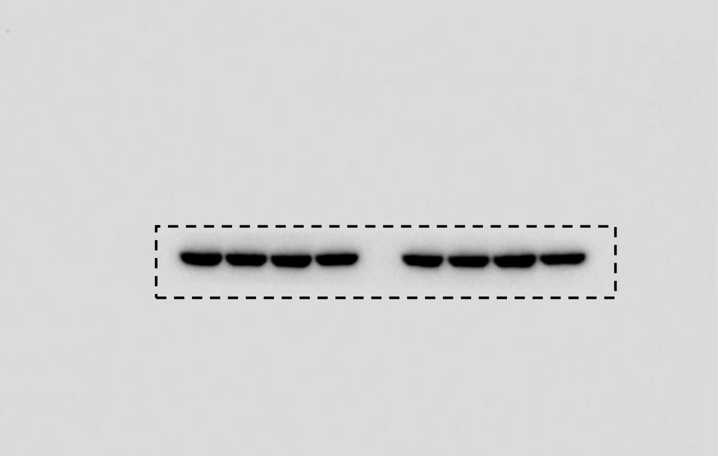

FIG2.C- C918 - β-actin

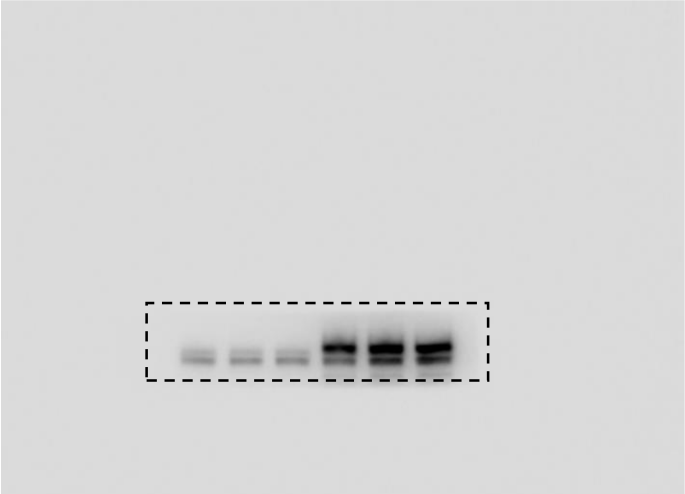

FIG2.E-C918-HDAC7

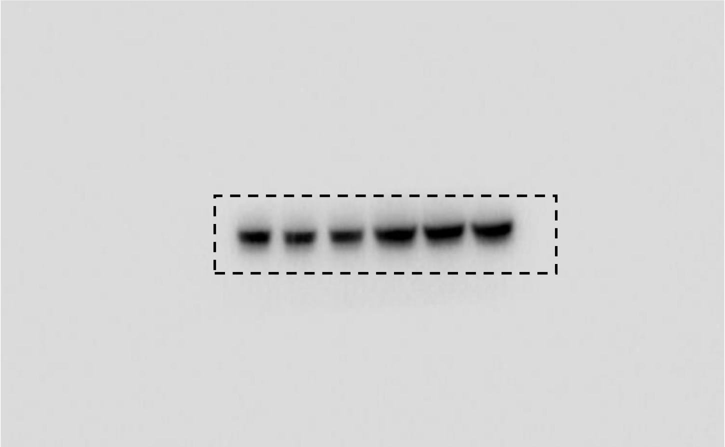

FIG2.E-C918- c-Myc

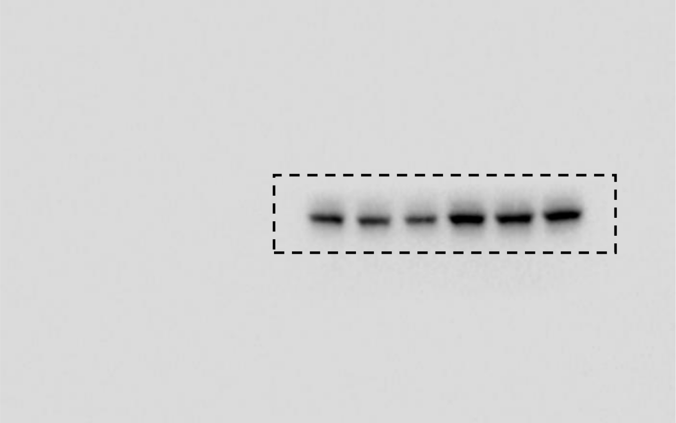

FIG2.E-C918- CDK1

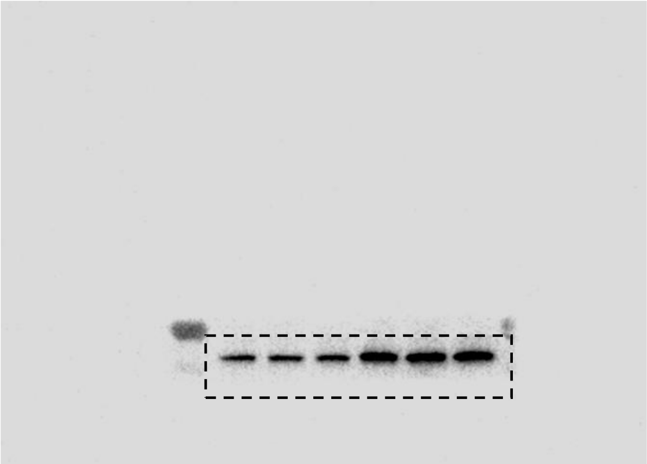

FIG2.E-C918- CyclinB1

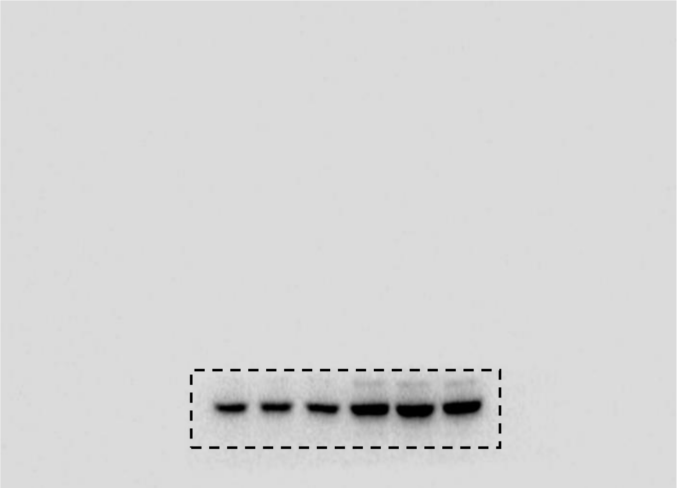

FIG2.E-C918- CDK2

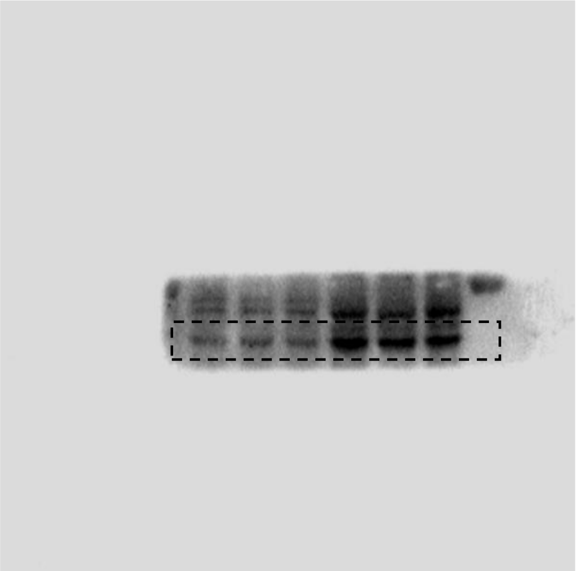

FIG2.E-C918- CyclinA2

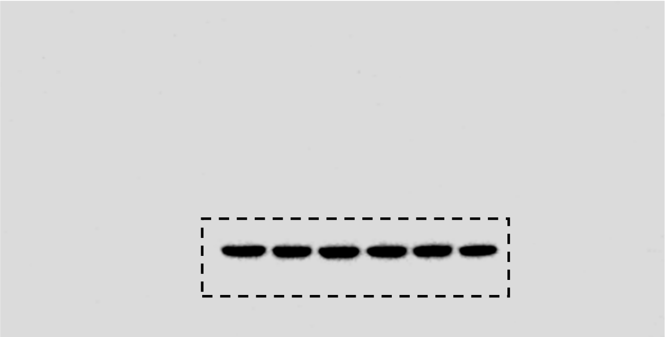

FIG2.E-C918- β-actin

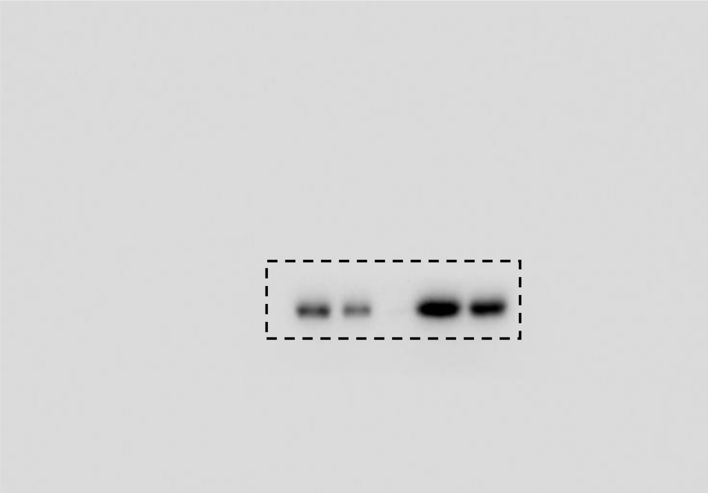

FIG2.F-C918-HDAC7

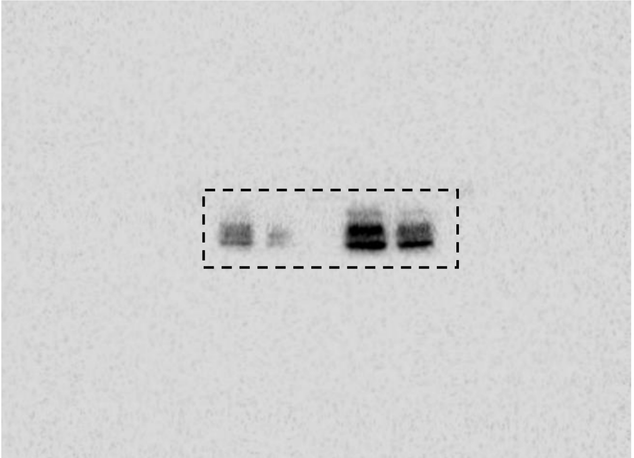

FIG2.F-C918- c-Myc

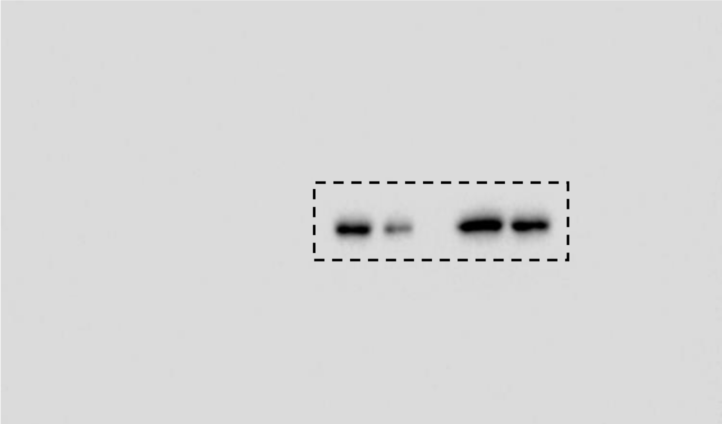

FIG2.F-C918- CDK1

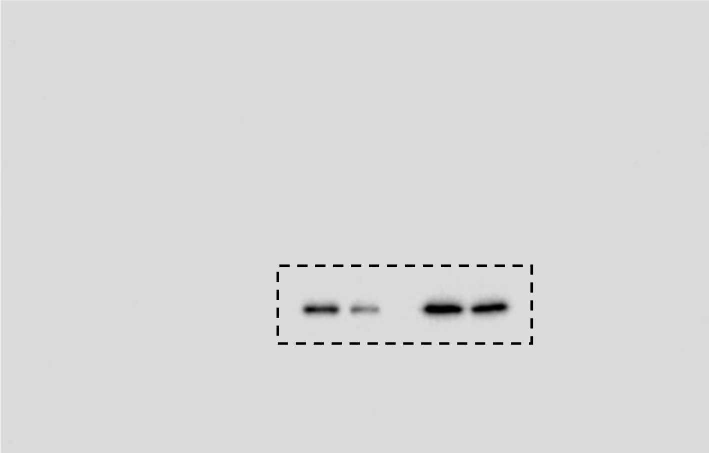

FIG2.F-C918- CyclinB1

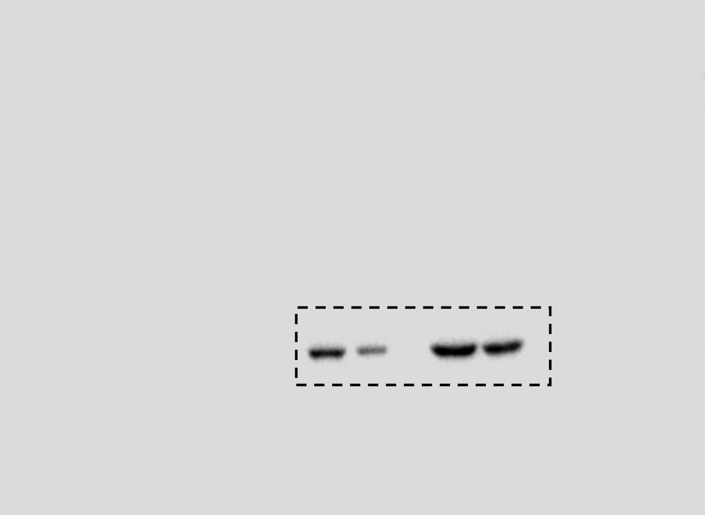

FIG2.F-C918- CDK2

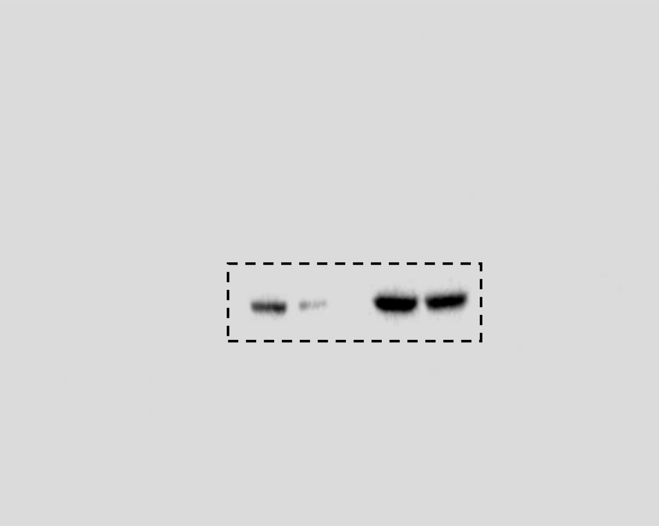

FIG2.F-C918- CyclinA2

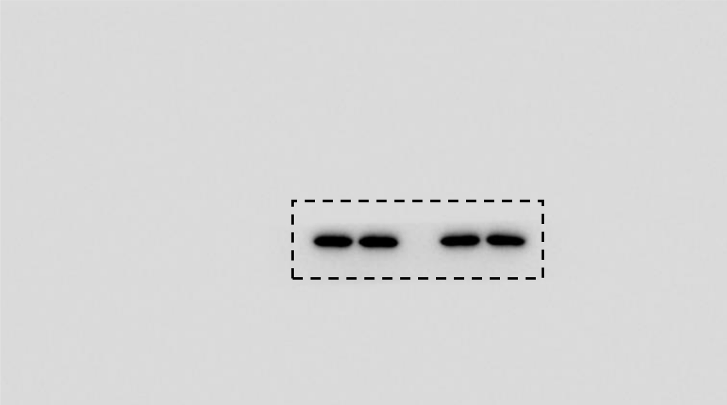

FIG2.F-C918- β-actin

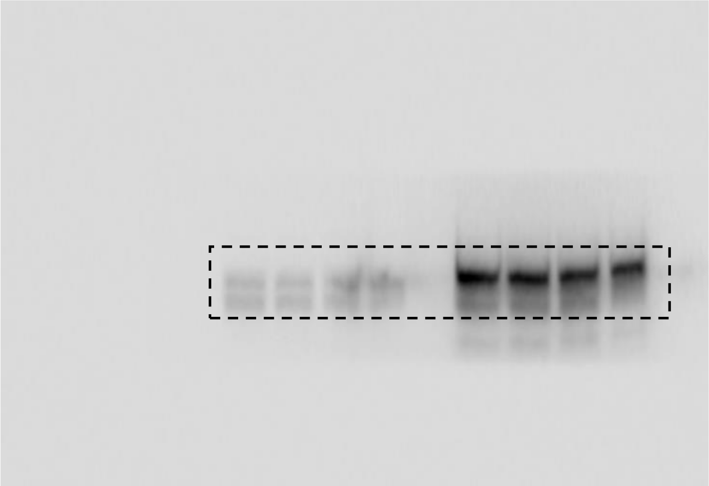

FIG3.C-OCM1- HDAC7

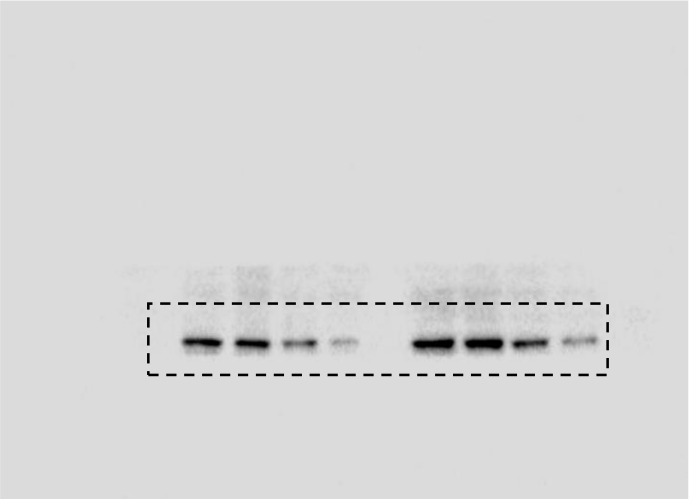

FIG3.C-OCM1- c-Myc

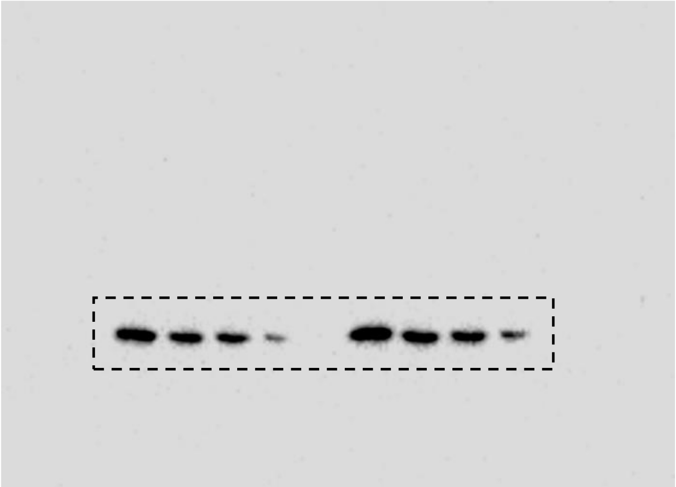

FIG3.C-OCM1-CDK1

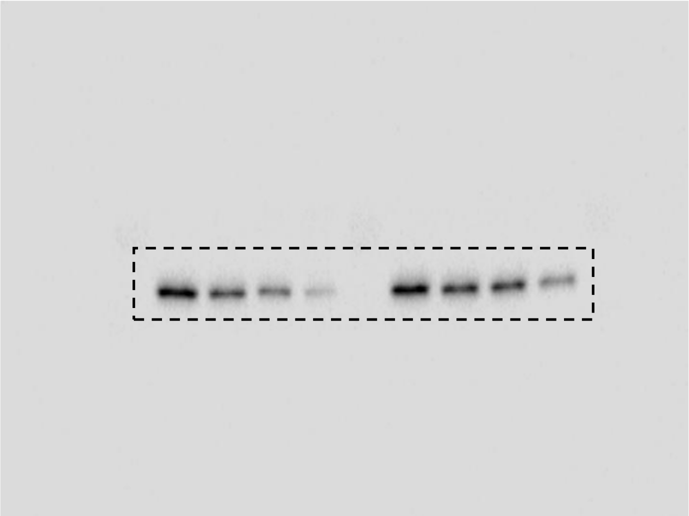

FIG3.C-OCM1- CyclinB1

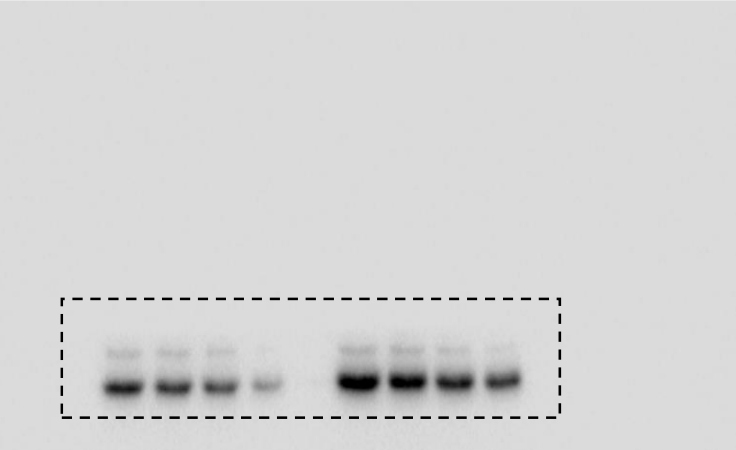

FIG3.C-OCM1-CDK2

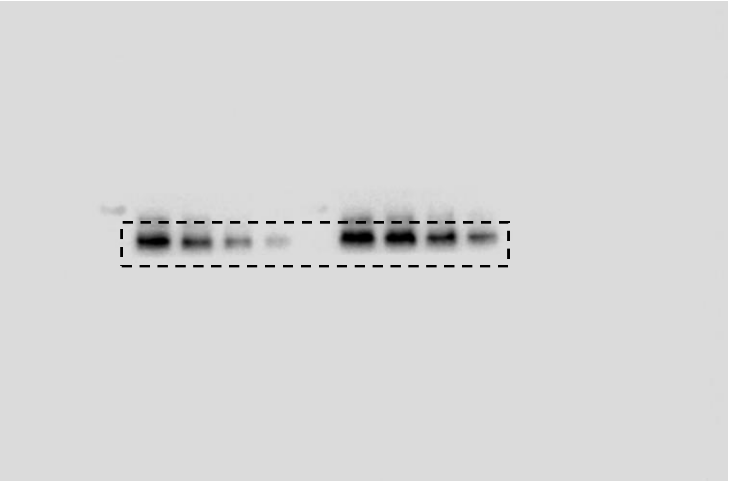

FIG3.C-OCM1- CyclinA2

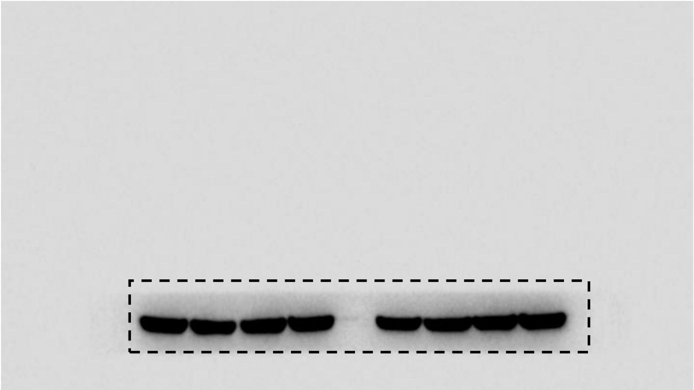

FIG3.C-OCM1- β-actin

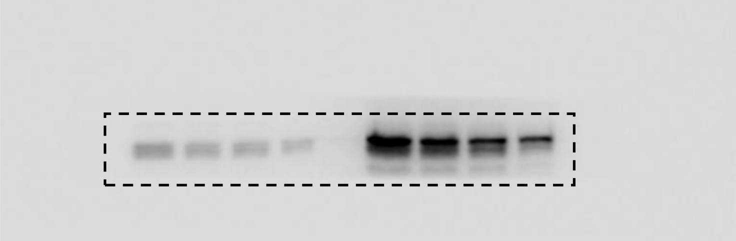

FIG3.C-C918- HDAC7

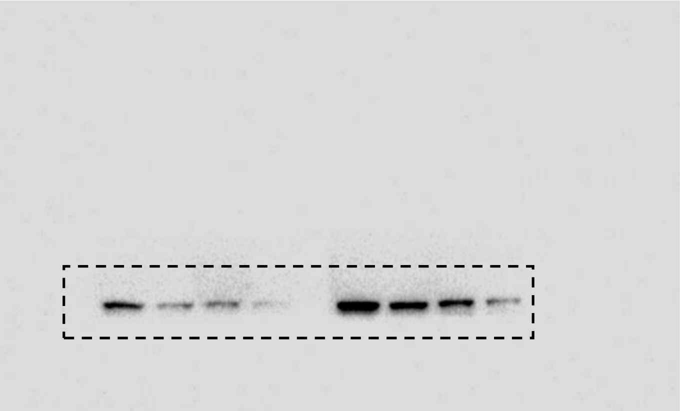

FIG3.C-C918- c-Myc

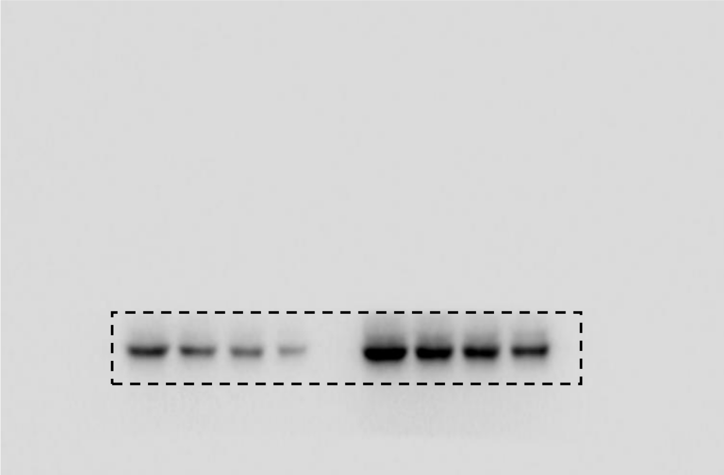

FIG3.C- C918 -CDK1

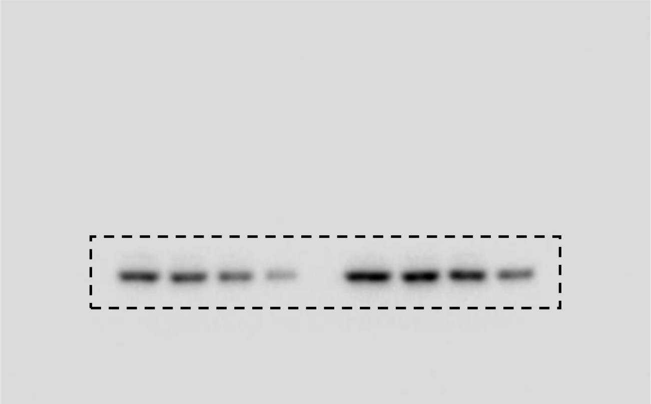

FIG3.C- C918 - CyclinB1

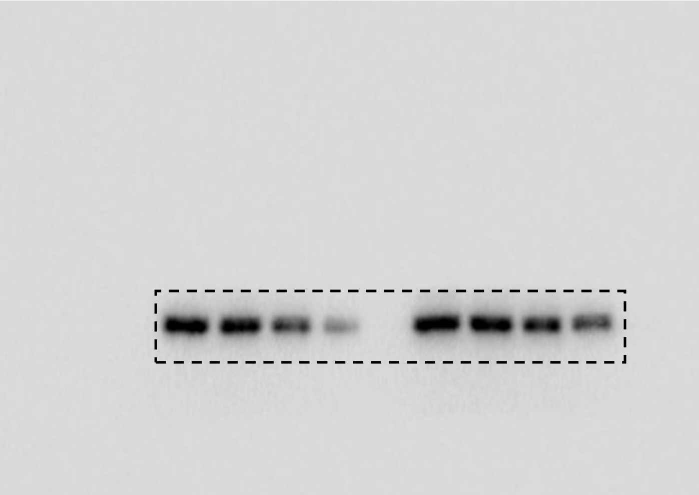

FIG3.C- C918 -CDK2

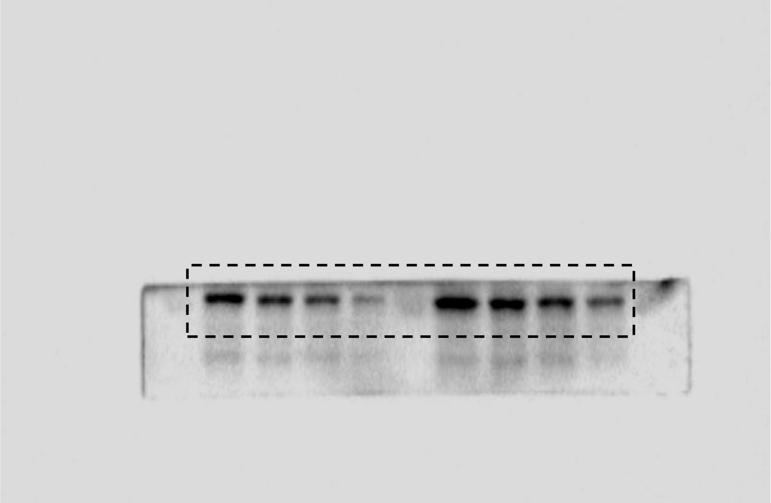

FIG3.C- C918 - CyclinA2

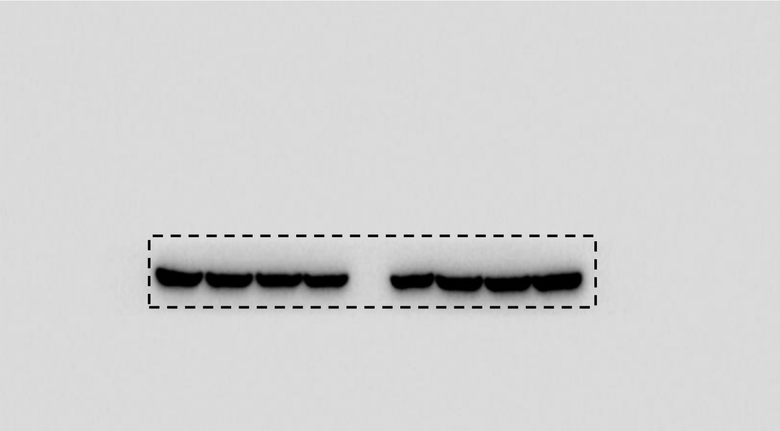

FIG3.C- C918 - β-actin

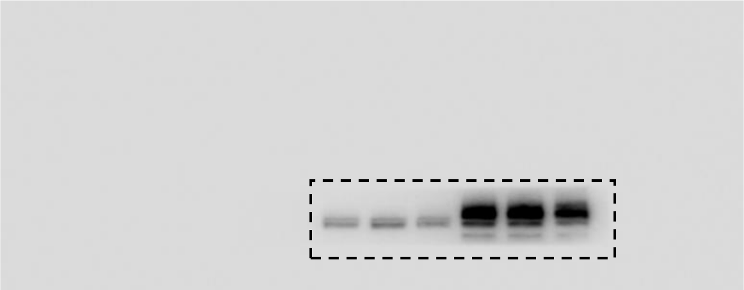

FIG4.C- OCM1 – HDAC7

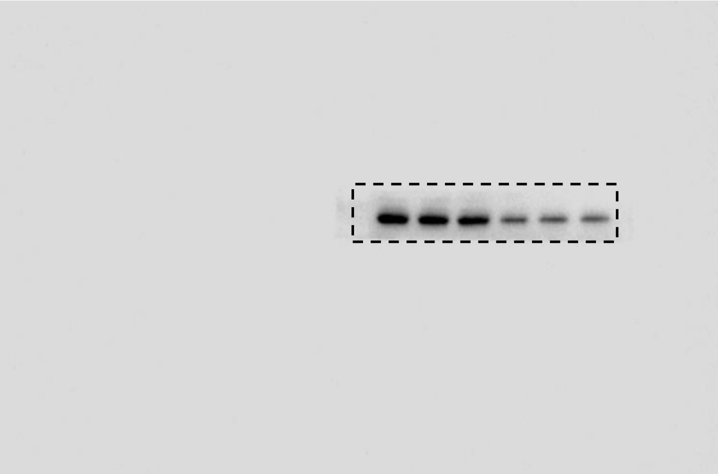

FIG4.C-OCM1- E-cadherin

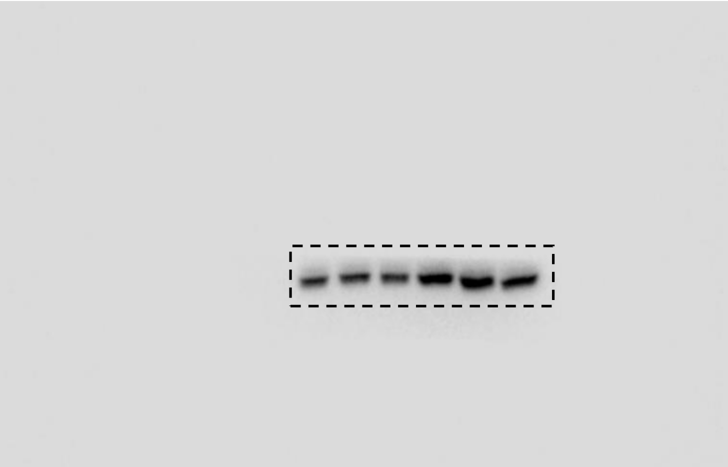

FIG4.C-OCM1- Zeb1

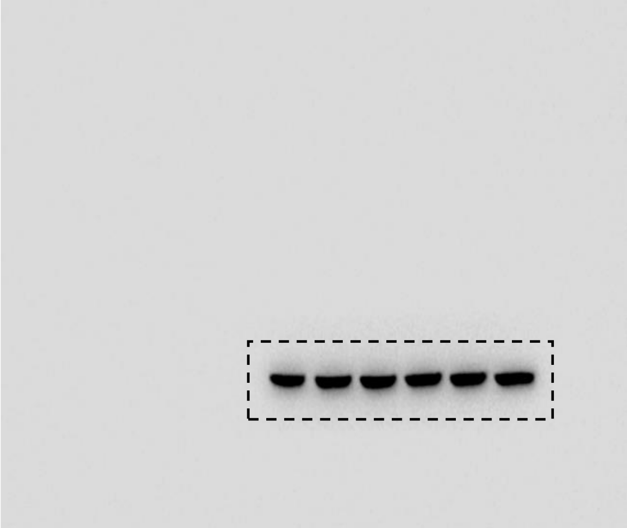

FIG4.C- OCM1 - α-SMA

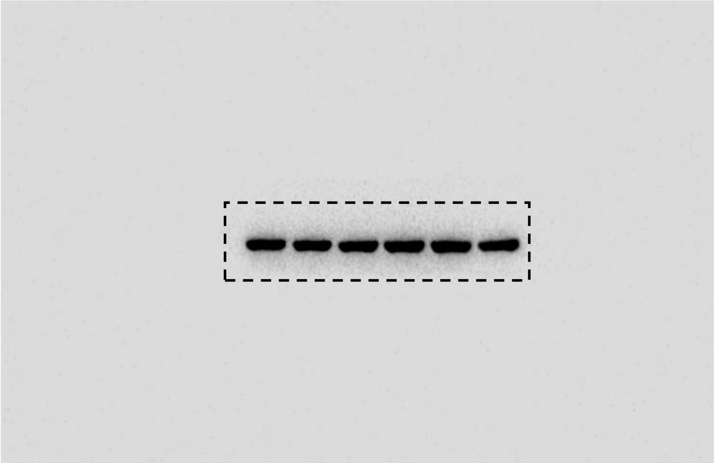

FIG4.C-OCM1- β-actin

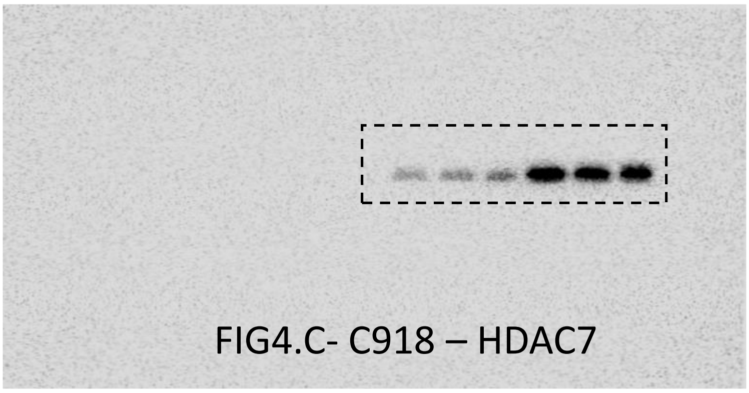

FIG4.C- C918 – HDAC7

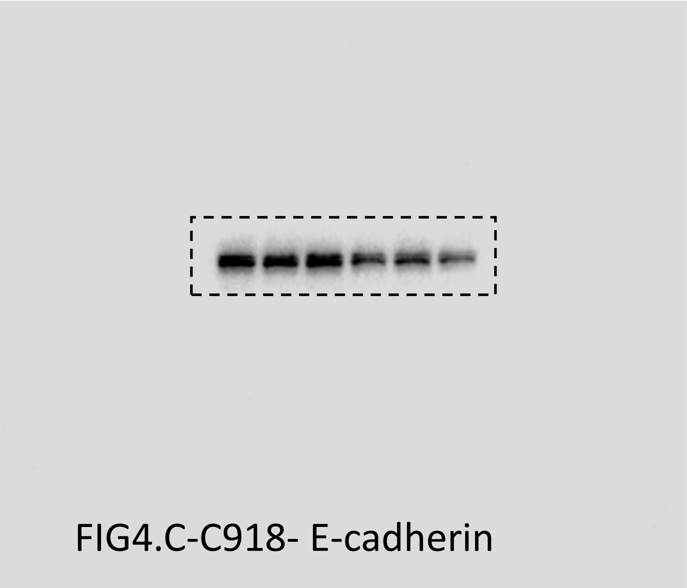

FIG4.C-C918- E-cadherin

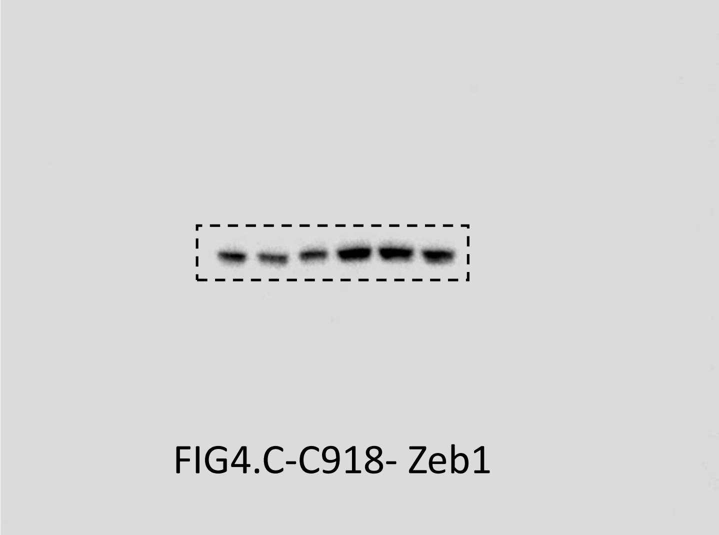

FIG4.C-C918- Zeb1

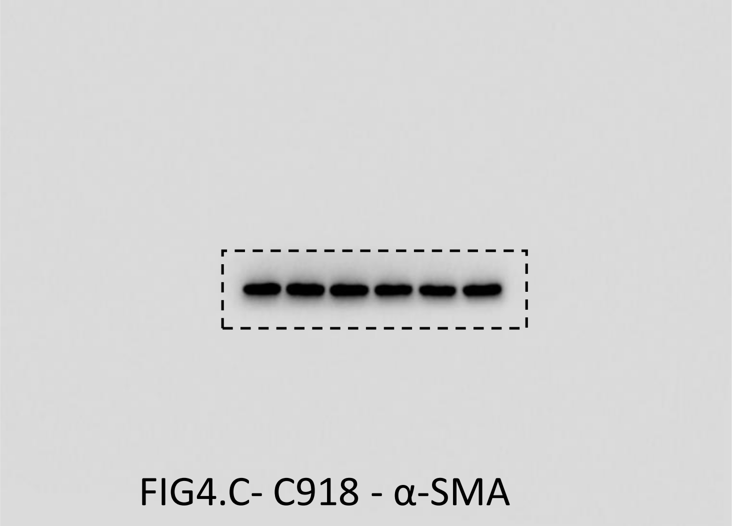

FIG4.C- C918 - α-SMA

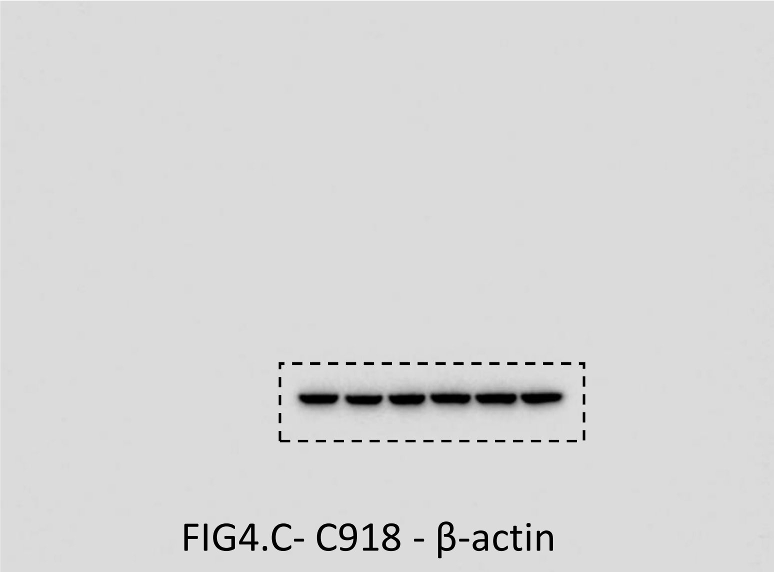

FIG4.C- C918 - β-actin

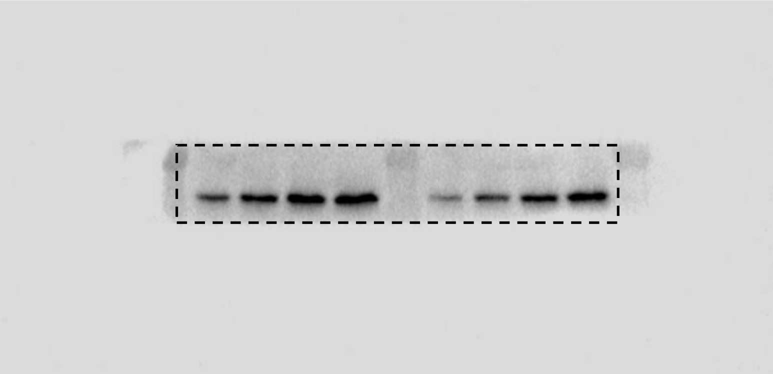

FIG5.C-OCM1- E-cadherin

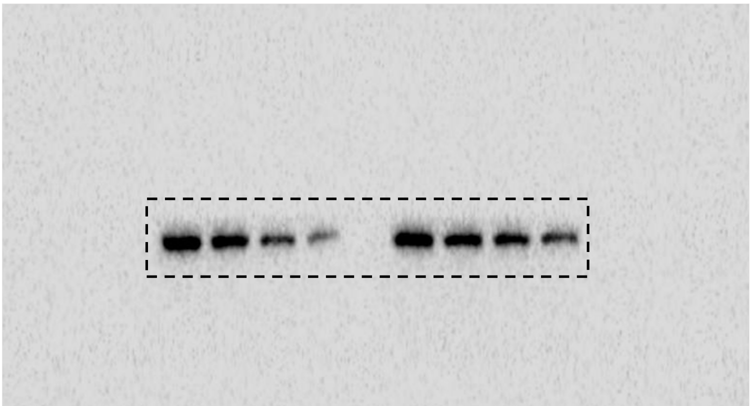

FIG5.C-OCM1- Zeb1

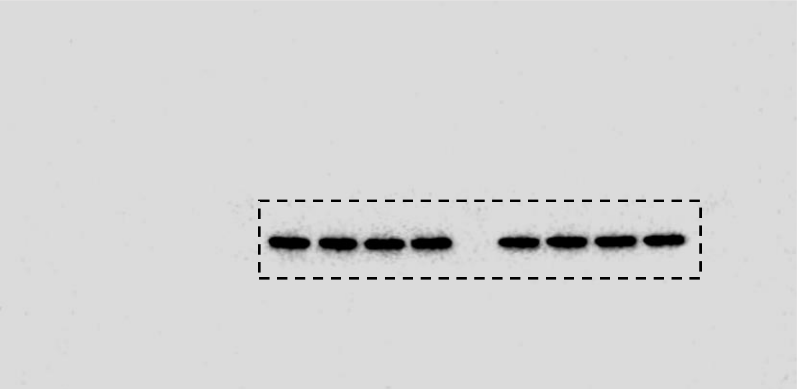

FIG5.C- OCM1 - α-SMA

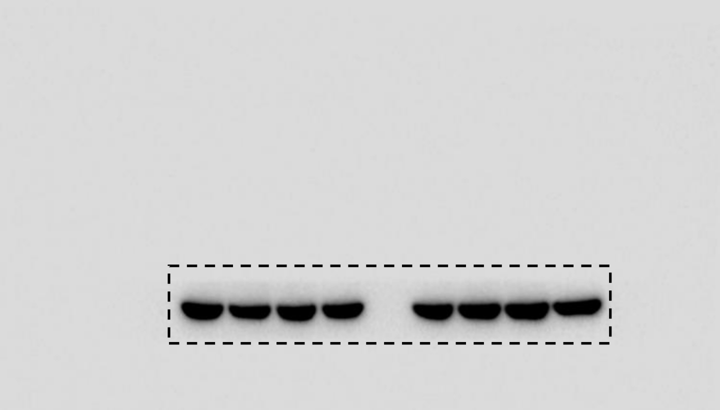

FIG5.C-OCM1- β-actin

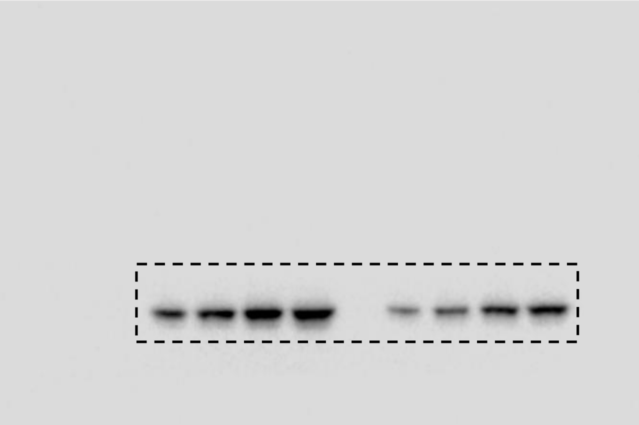

FIG5.C-C918- E-cadherin

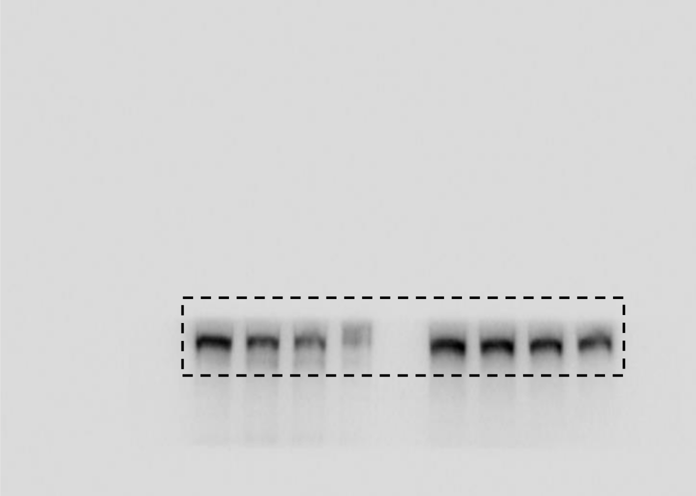

FIG5.C-C918- Zeb1

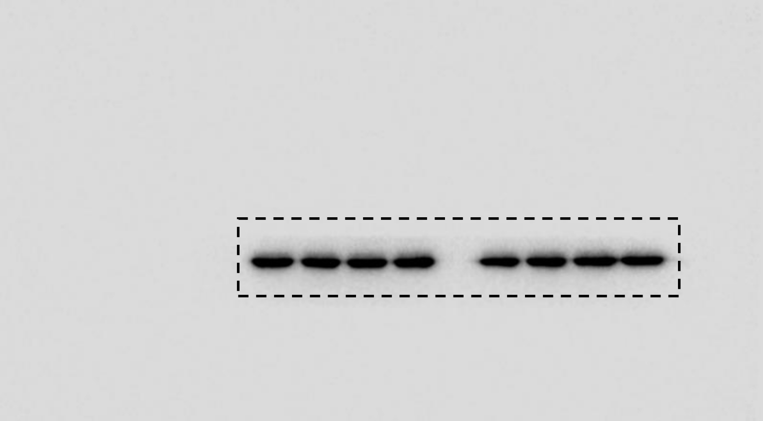

FIG5.C- C918 - α-SMA

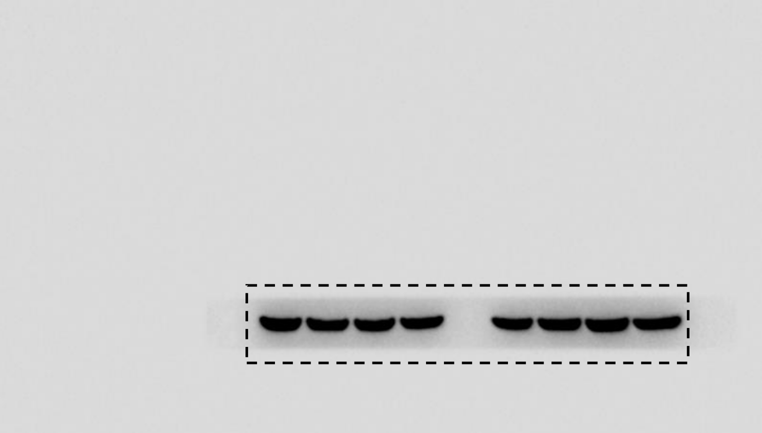

FIG5.C-C918- β-actin

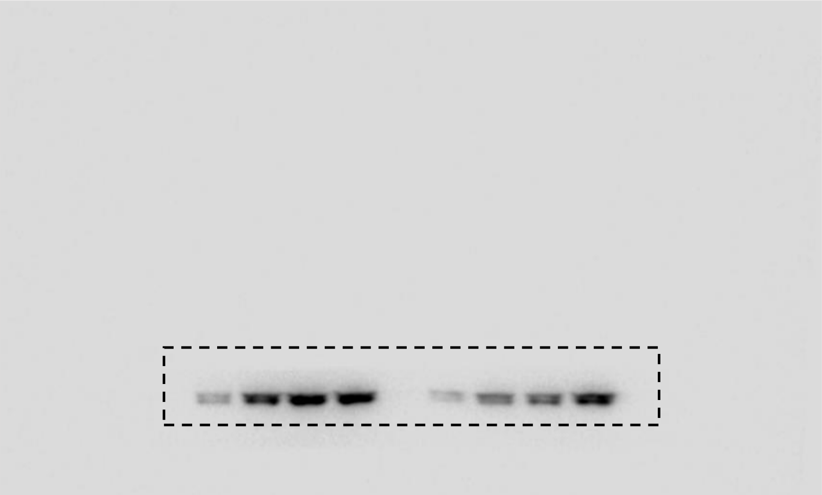

FIG6.C-OCM1- E-cadherin

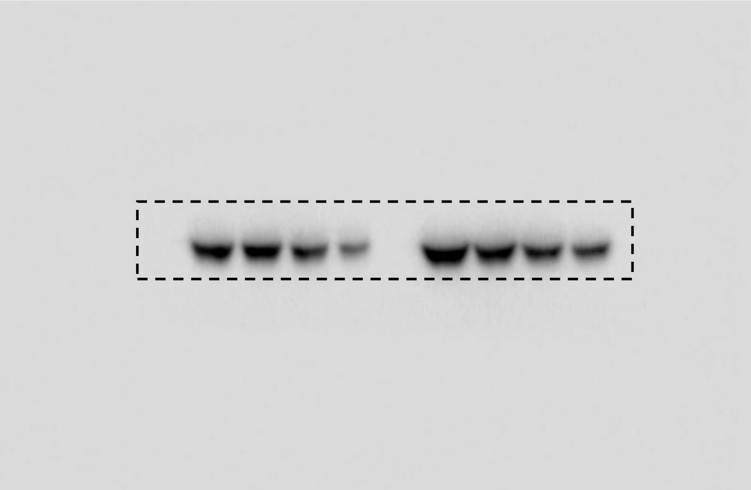

FIG6.C-OCM1- Zeb1

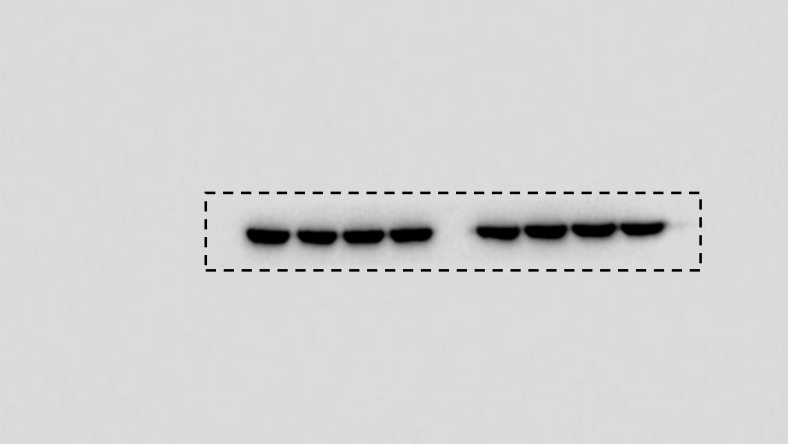

FIG6.C- OCM1 - α-SMA

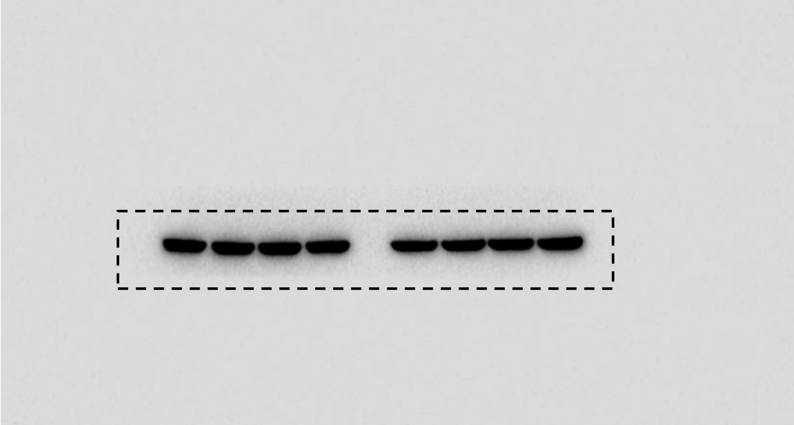

FIG6.C-OCM1- β-actin

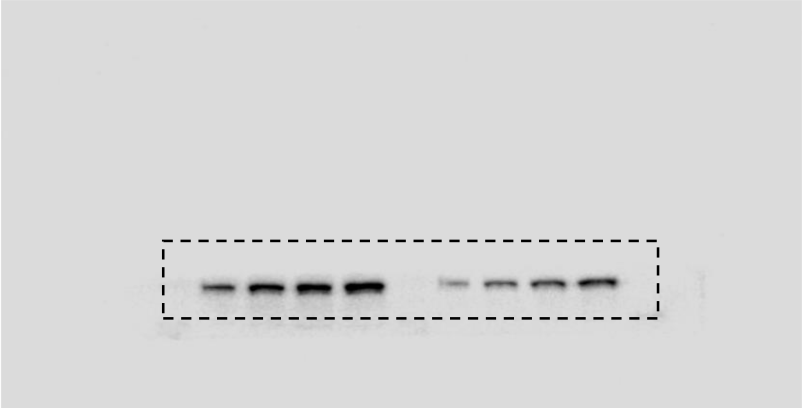

FIG6.C-C918- E-cadherin

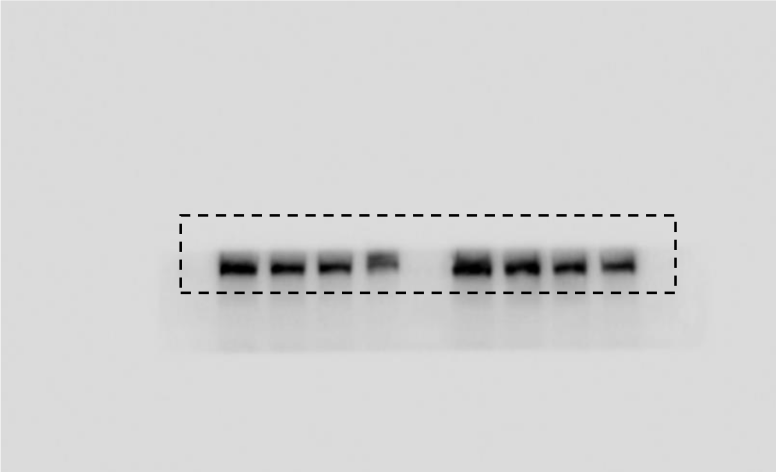

FIG6.C-C918- Zeb1

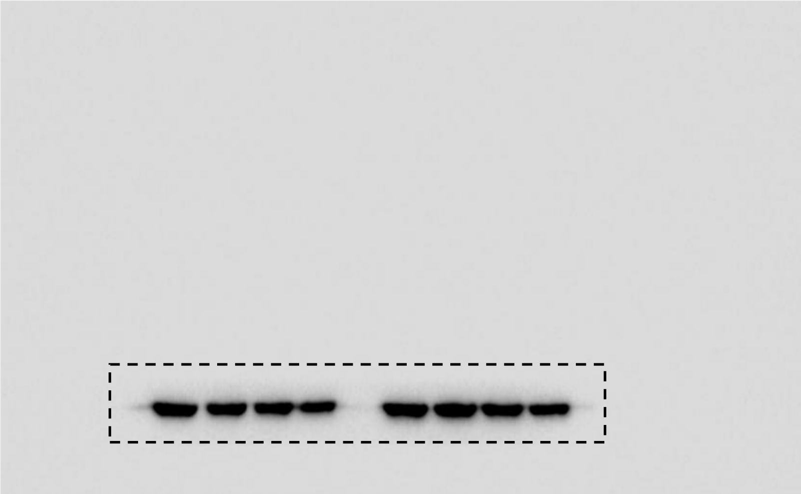

FIG6.C- C918 - α-SMA

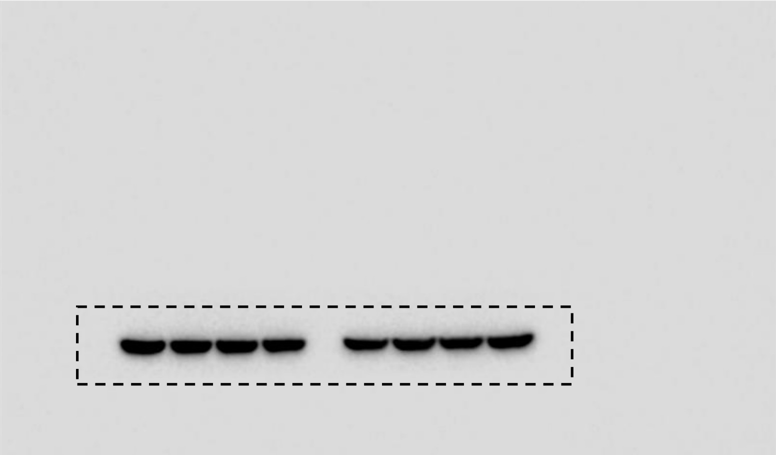

FIG6.C-C918- β-actin

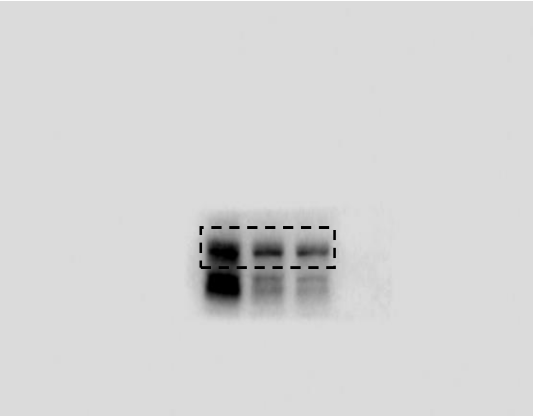

FIG7.C-OCM1- c-Myc

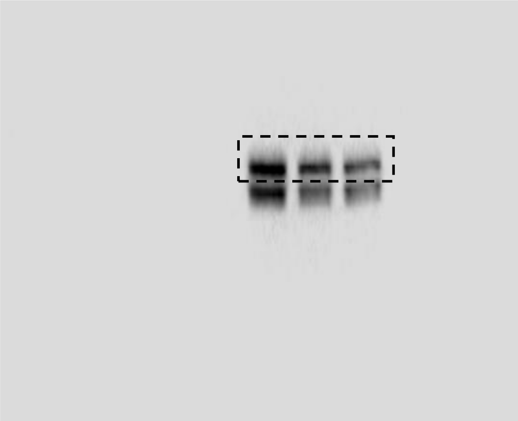

FIG7.C-OCM1-HDAC7

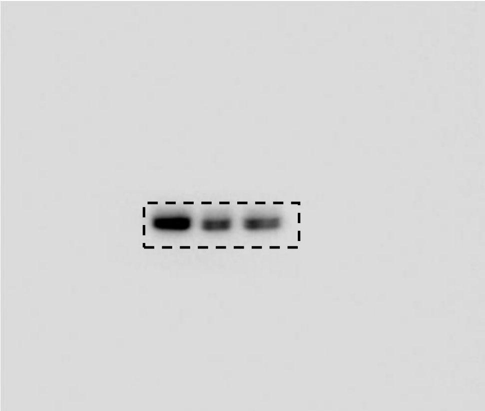

FIG7.C-OCM1-CDK1

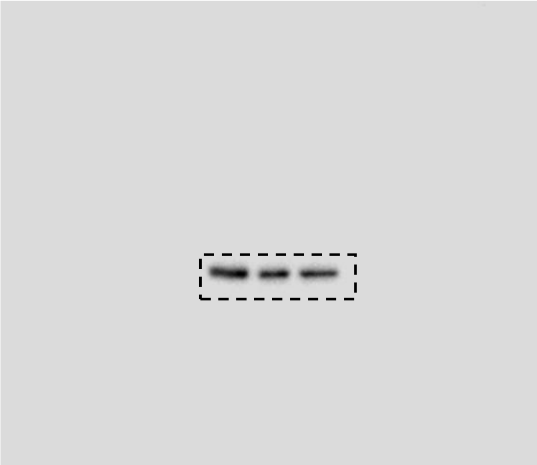

FIG7.C-OCM1- CyclinB1

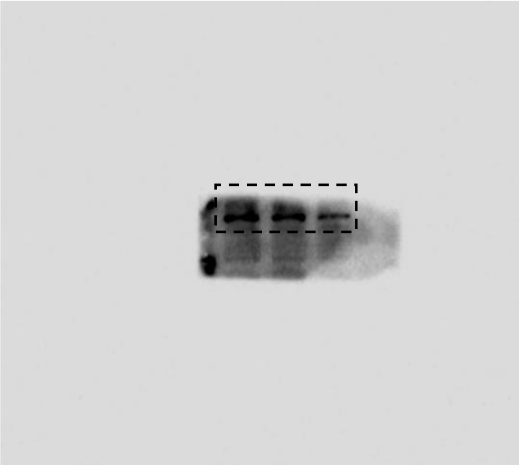

FIG7.C-OCM1-CDK2

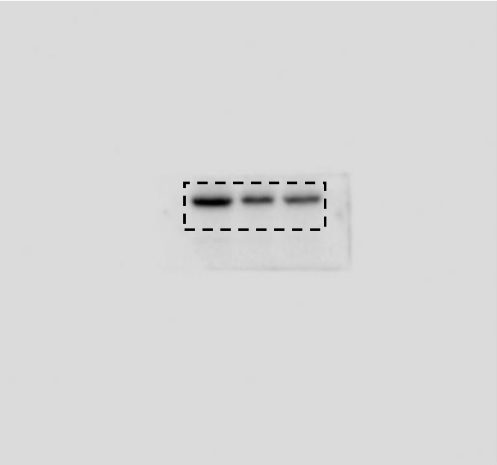

FIG7.C-OCM1- CyclinA2

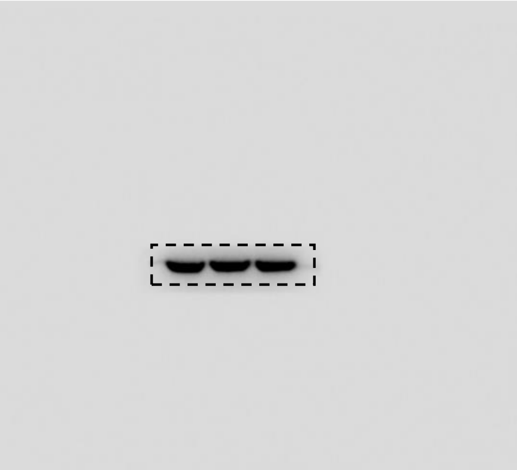

FIG7.C-OCM1- β-actin

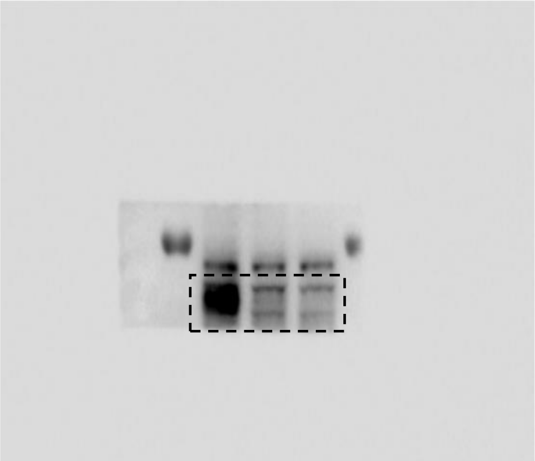

FIG7.C-C918- c-Myc

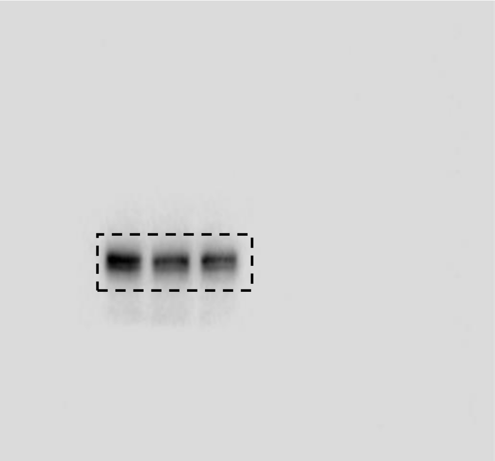

FIG7.C- C918 -HDAC7

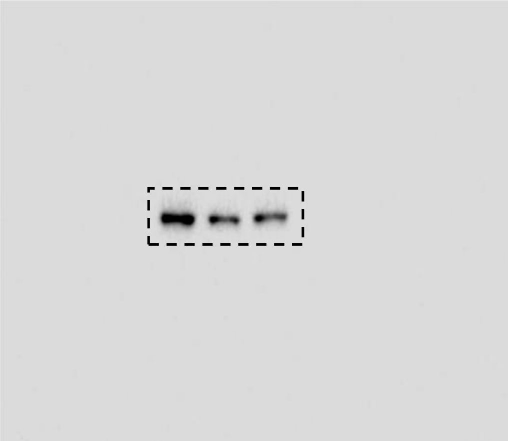

FIG7.C- C918 -CDK1

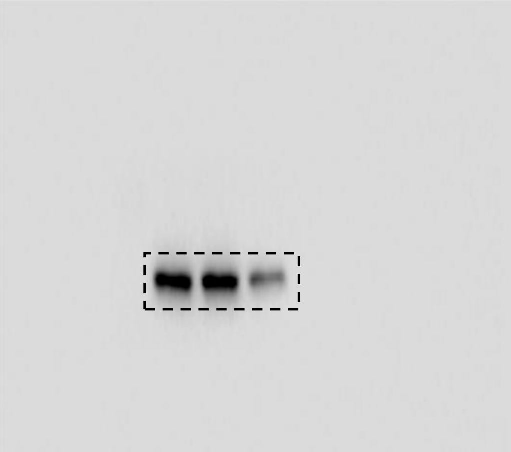

FIG7.C- C918 - CyclinB1

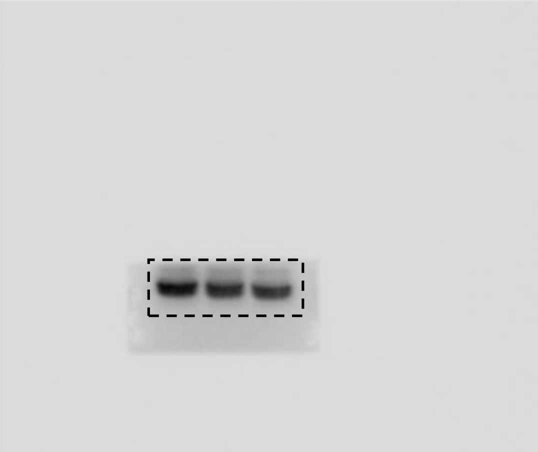

FIG7.C- C918 -CDK2

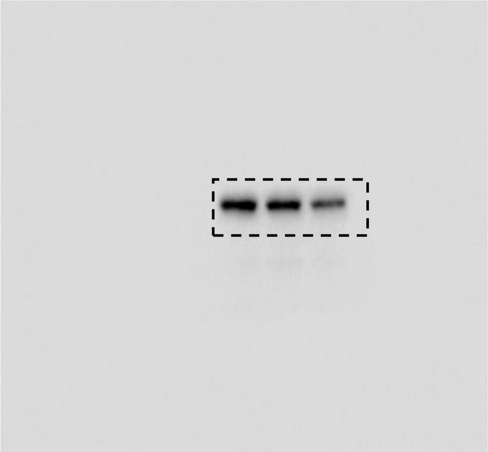

FIG7.C- C918 - CyclinA2

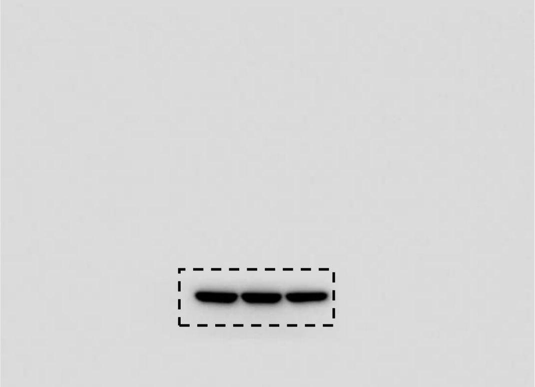

FIG7.C- C918 - β-actin

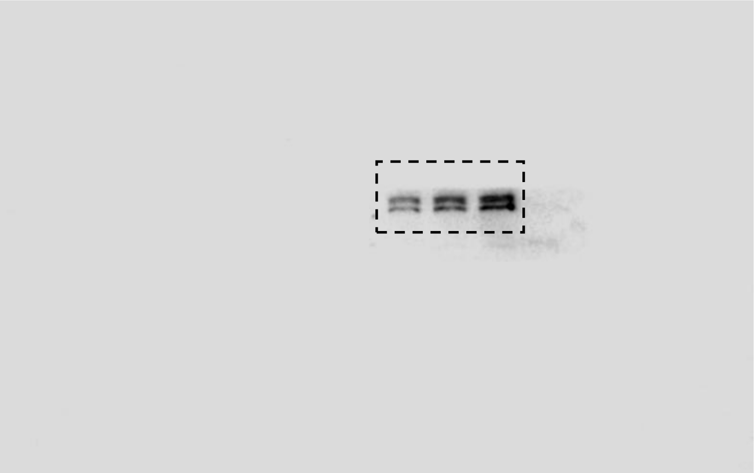

FIG7.F-OCM1- E-cadherin

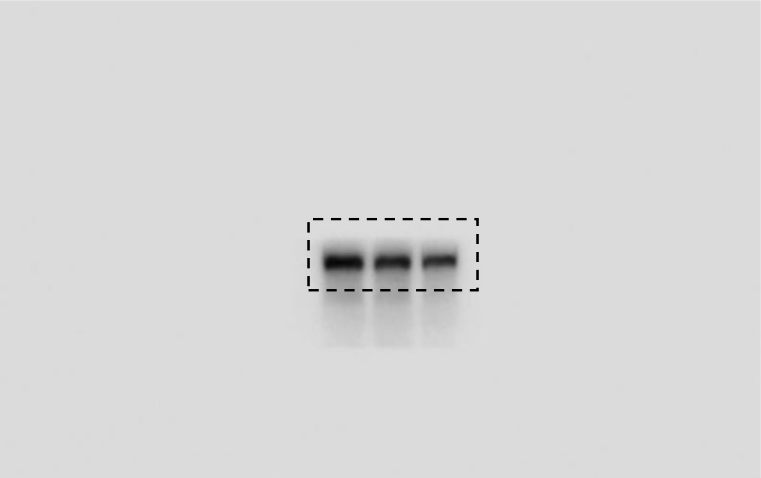

FIG7.F-OCM1- Zeb1

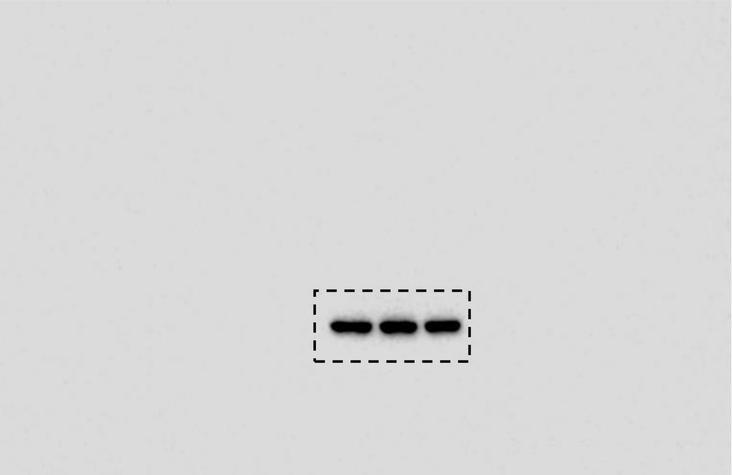

FIG7.F- OCM1 - α-SMA

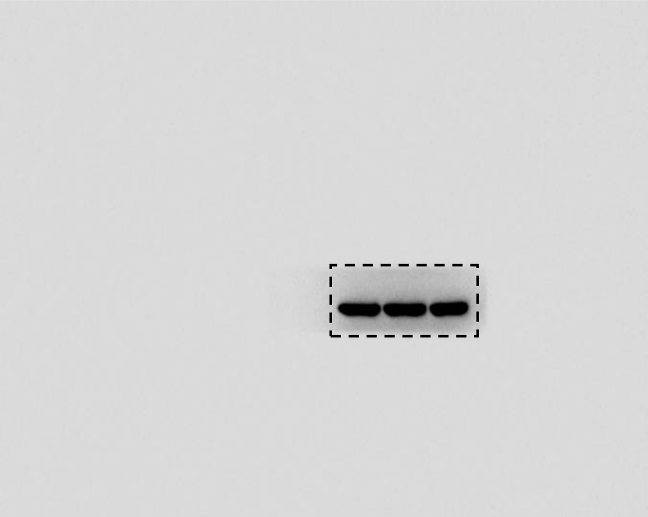

FIG7.F-OCM1- β-actin

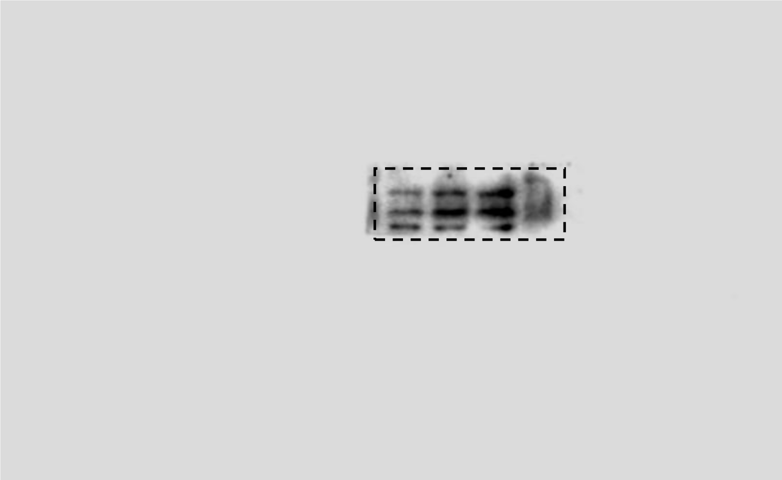

FIG7.F-C918- E-cadherin

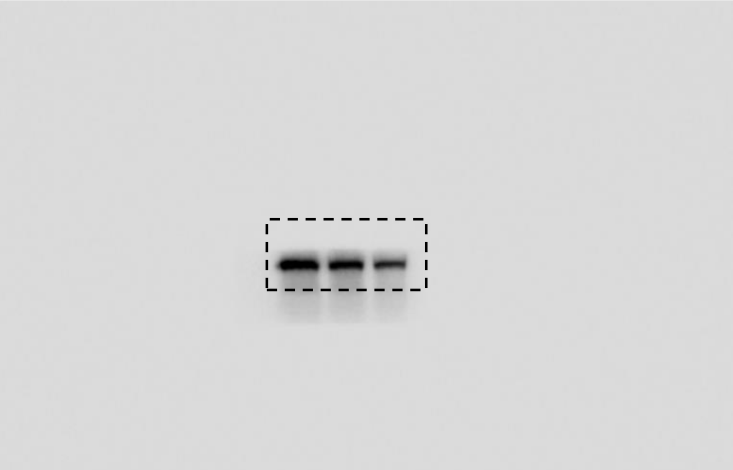

FIG7.F-C918- Zeb1

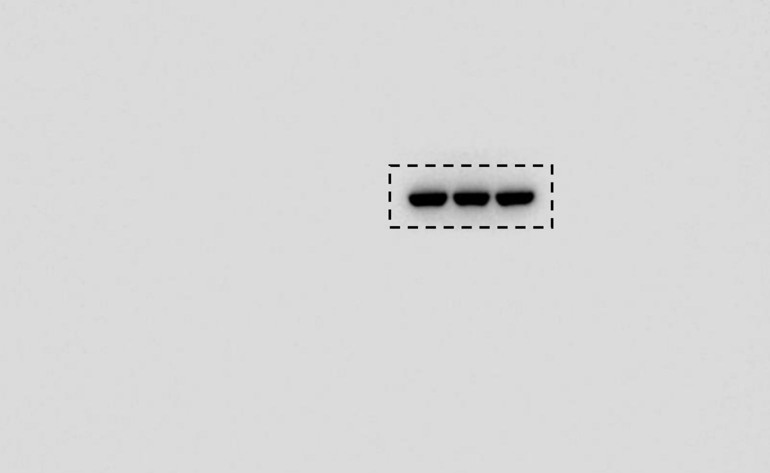

FIG7.F- C918 - α-SMA

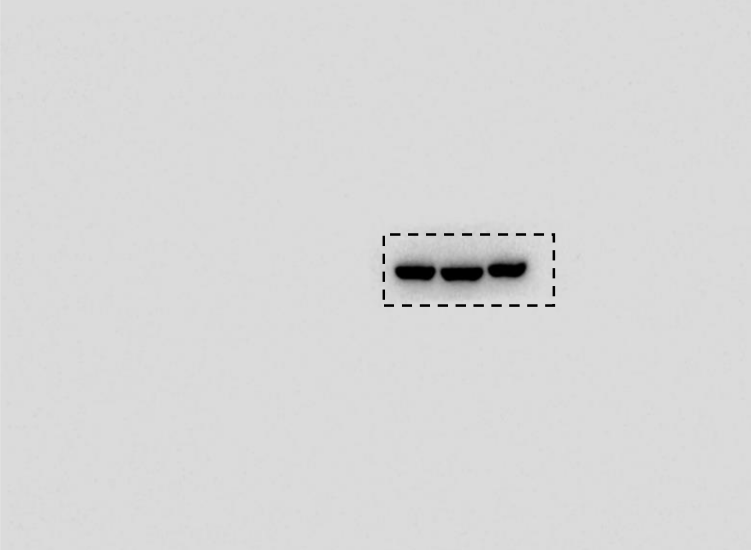

FIG7.F-C918- β-actin

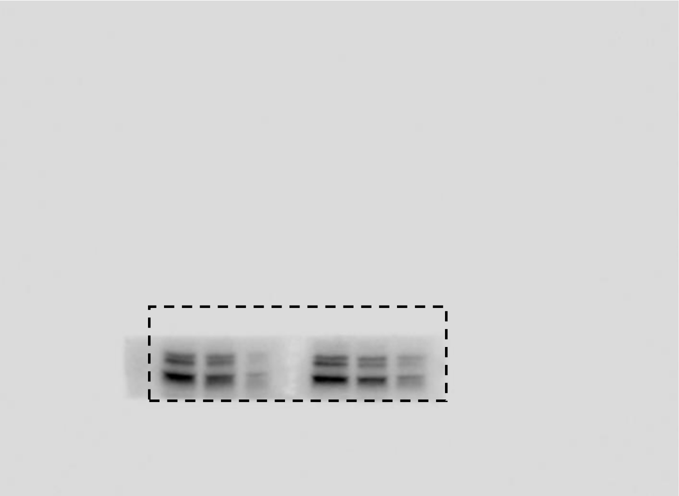

FIG8.C-OCM1- c-Myc

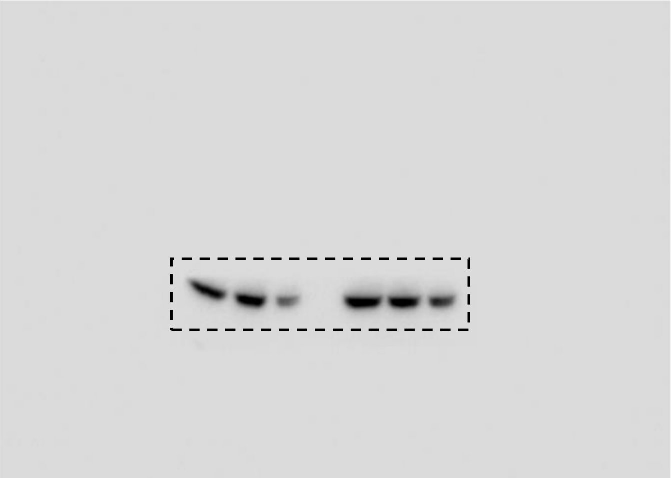

FIG8.C-OCM1-CDK1

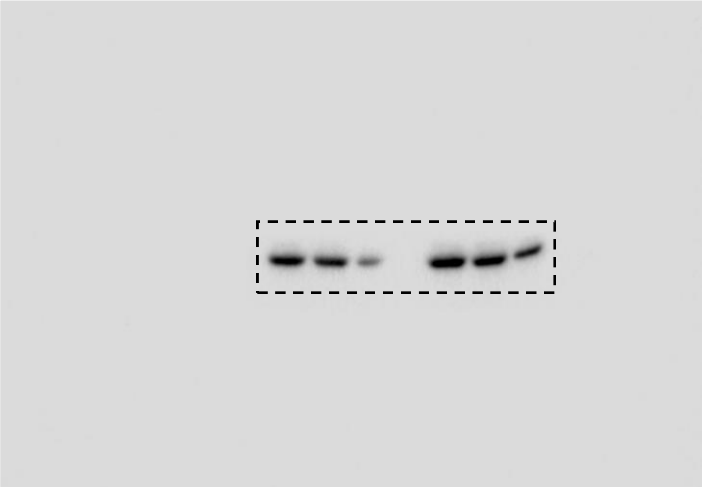

FIG8.C-OCM1-CDK2

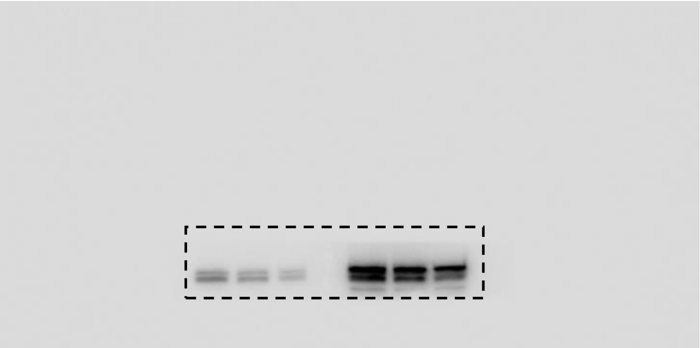

FIG8.C-OCM1-HDAC7

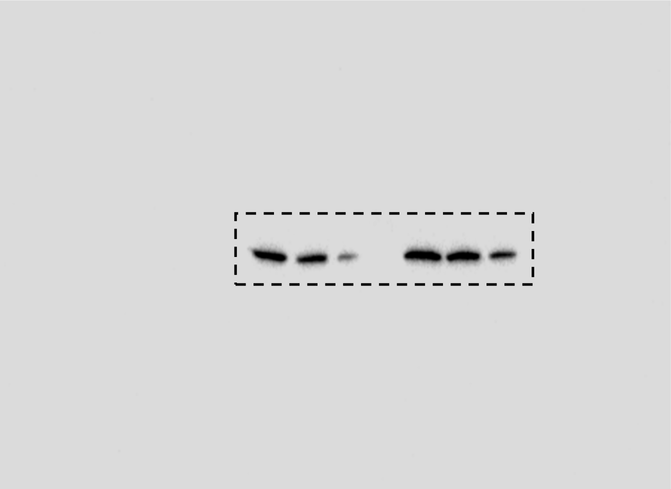

FIG8.C-OCM1- CyclinB1

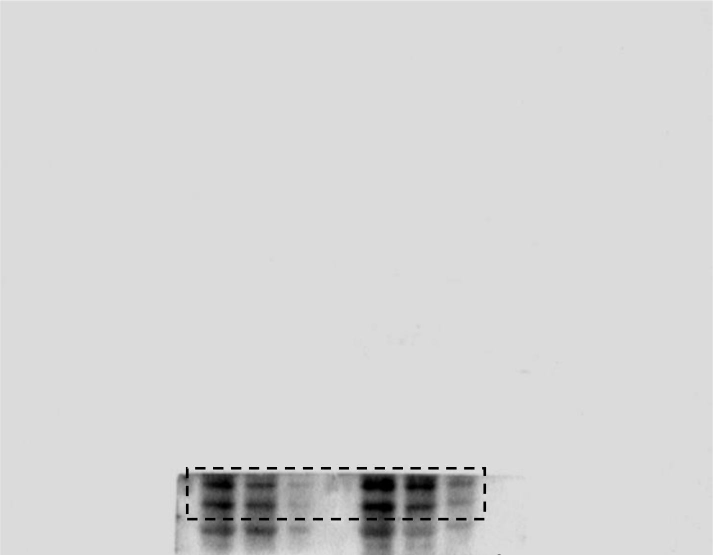

FIG8.C-OCM1- CyclinA2

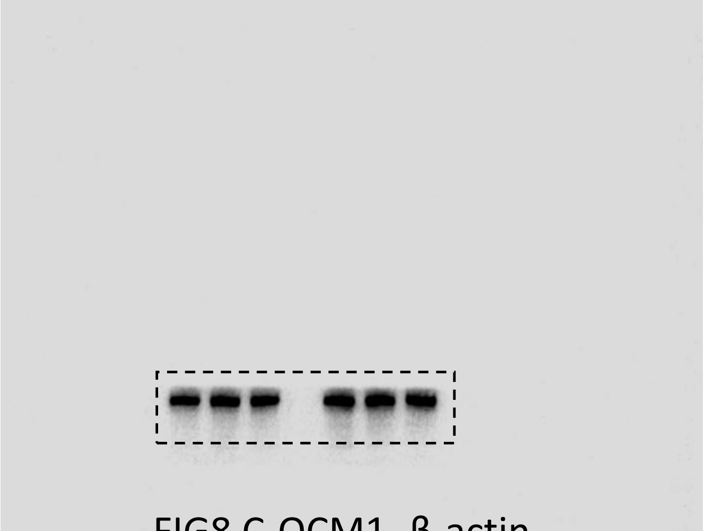

FIG8.C-OCM1- β-actin

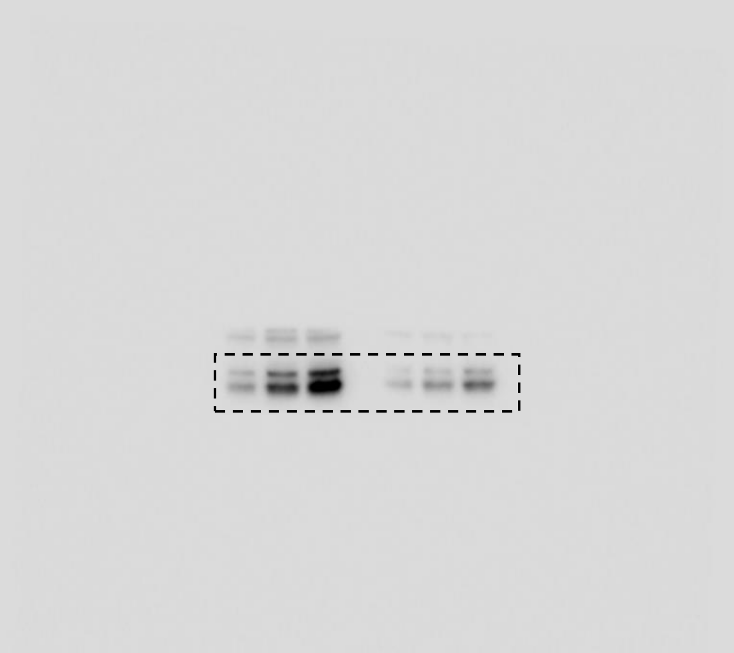
FIG8.F-OCM1- E-cadherin

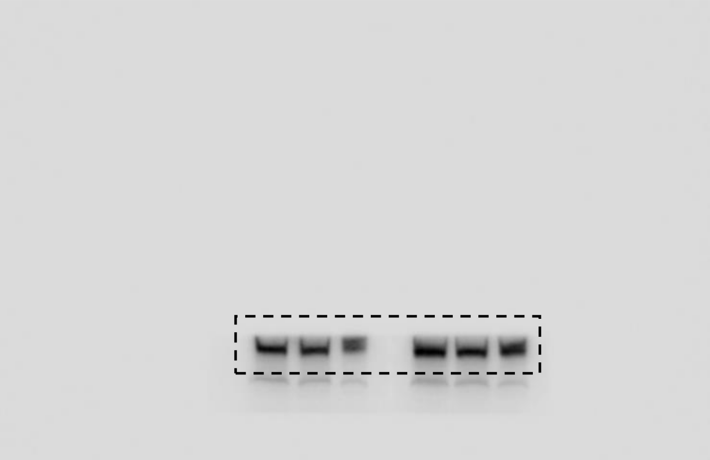
FIG8.F-OCM1- Zeb1

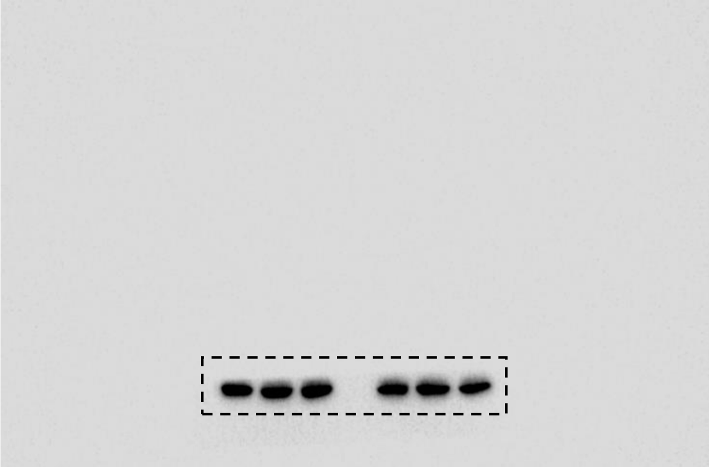
FIG8.F- OCM1 - α-SMA

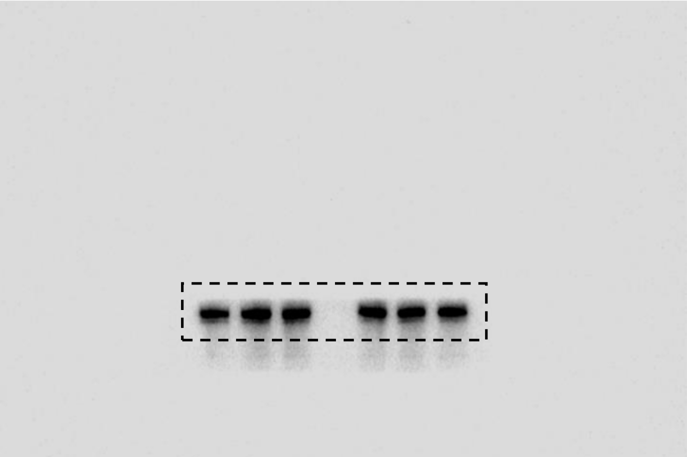
FIG8.F-OCM1- β-actin

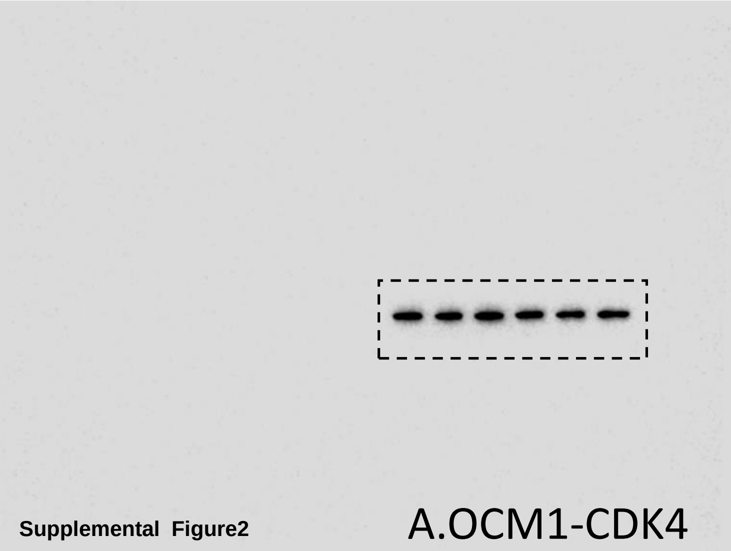
Supplemental Figure2
A.OCM1-CDK4

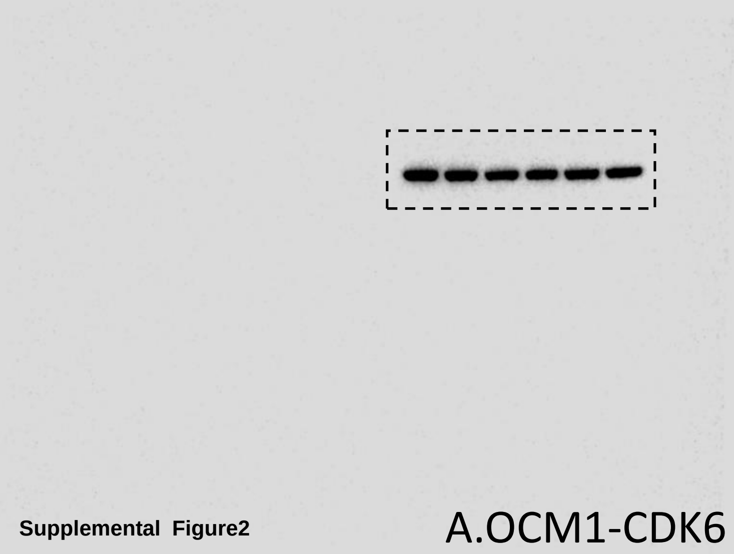
Supplemental Figure2
A.OCM1-CDK6

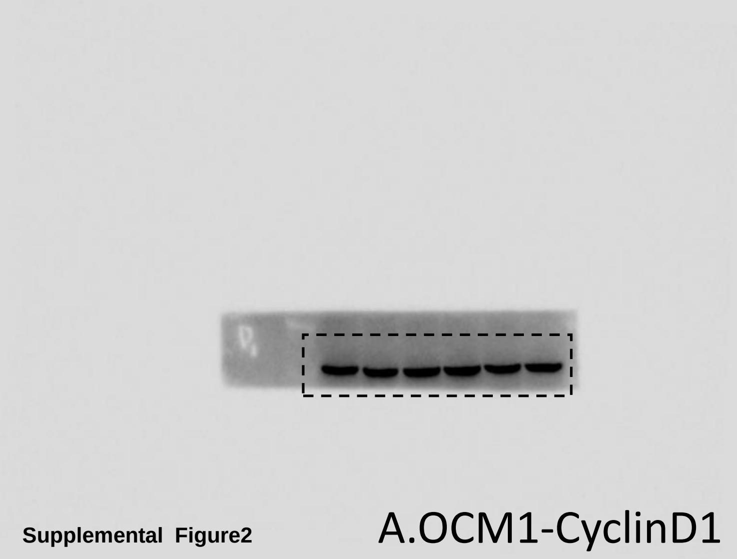
Supplemental Figure2
A.OCM1-CyclinD1

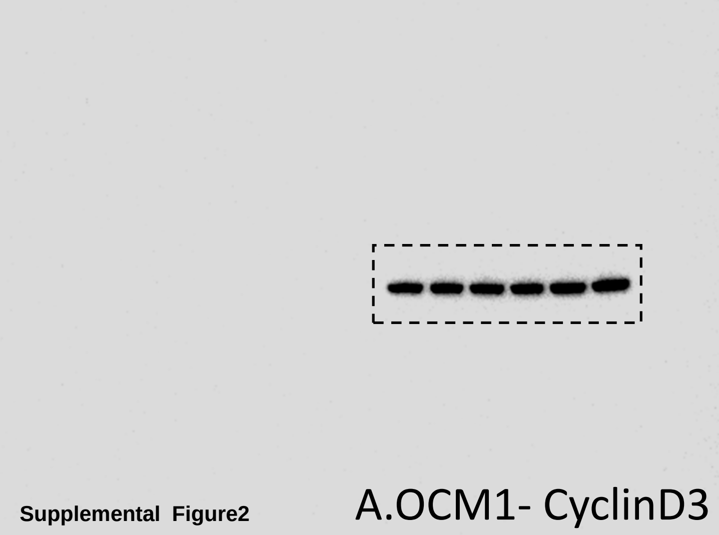
Supplemental Figure2
A.OCM1- CyclinD3

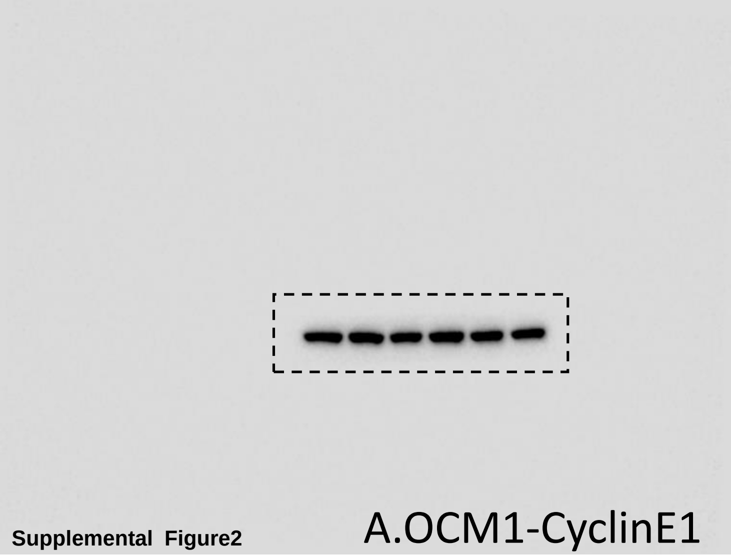
Supplemental Figure2
A.OCM1-CyclinE1

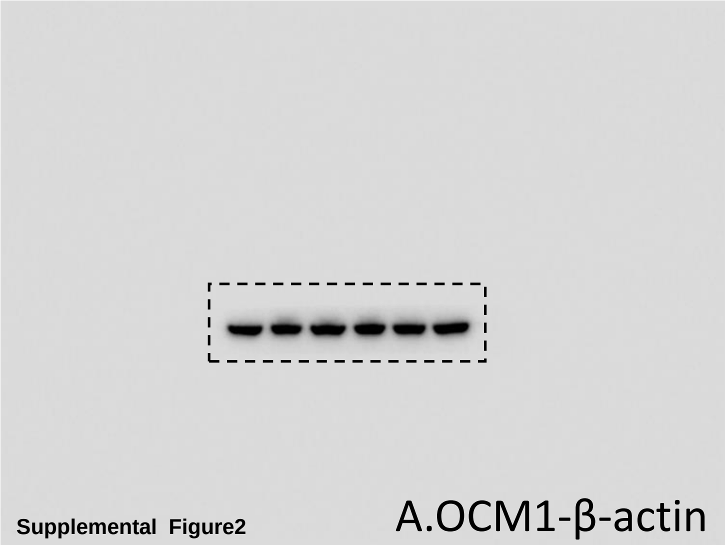
Supplemental Figure2
A.OCM1-β-actin

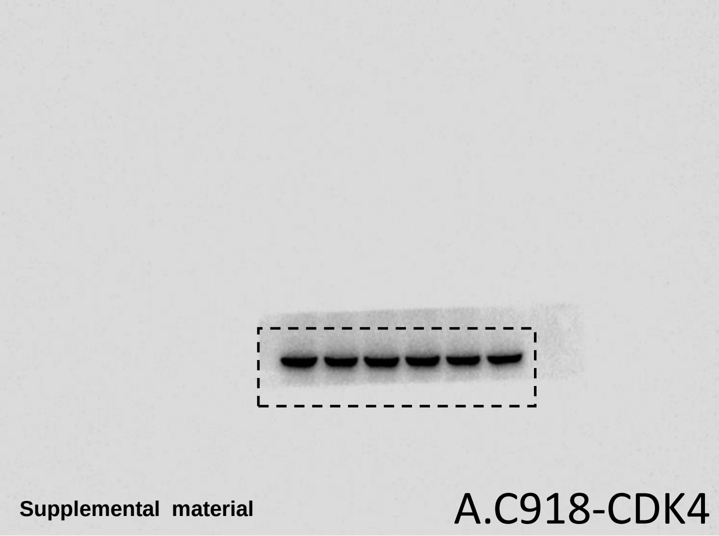
Supplemental material
A.C918-CDK4

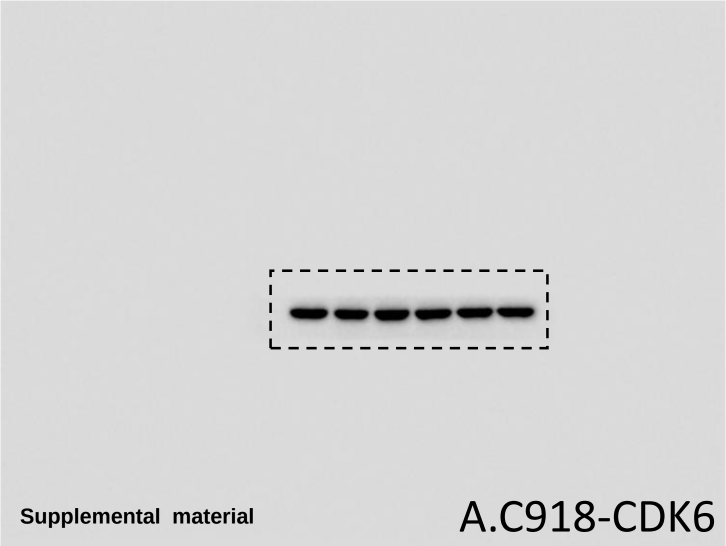
Supplemental material
A.C918-CDK6

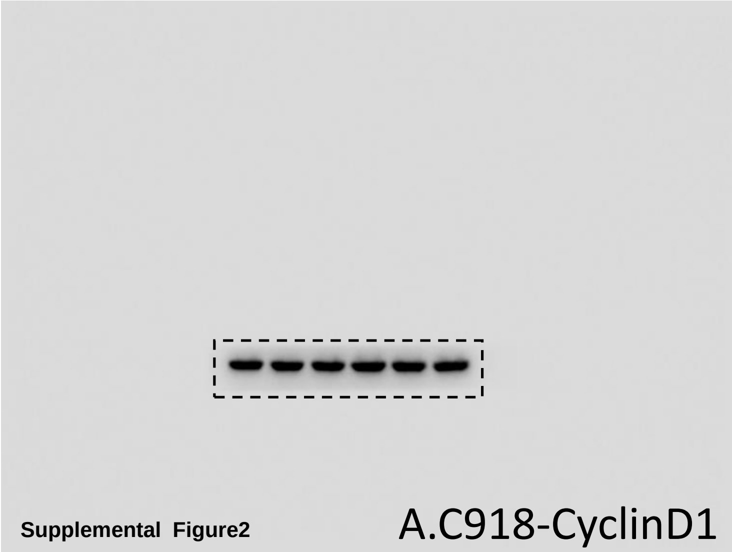
Supplemental Figure2
A.C918-CyclinD1

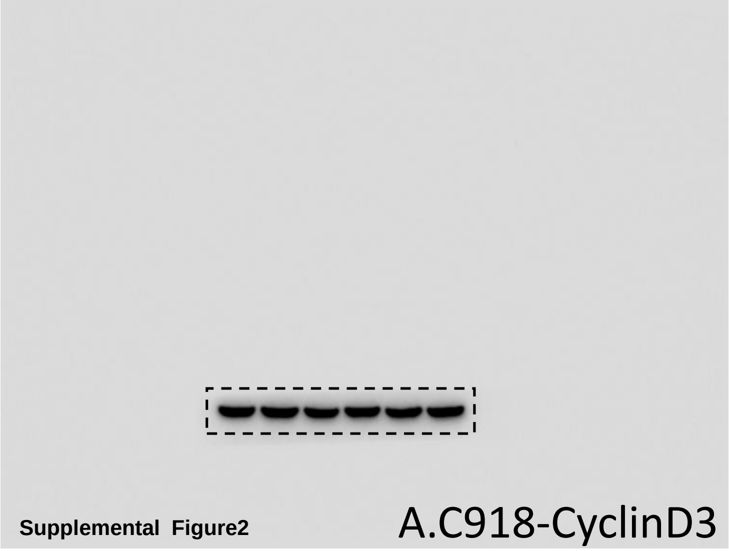
Supplemental Figure2
A.C918-CyclinD3

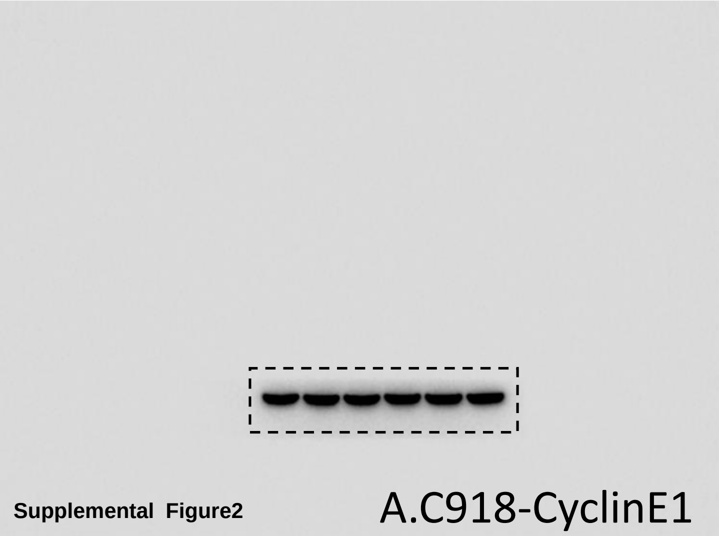
Supplemental Figure2
A.C918-CyclinE1

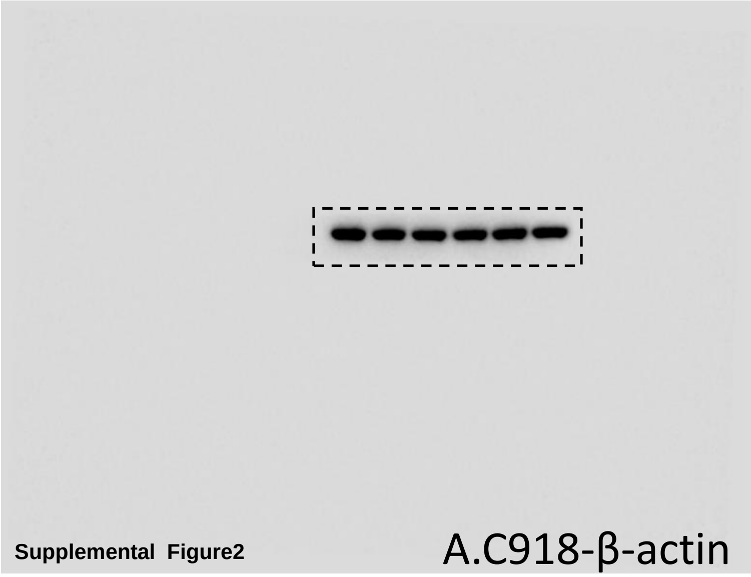
Supplemental Figure2
A.C918-β-actin

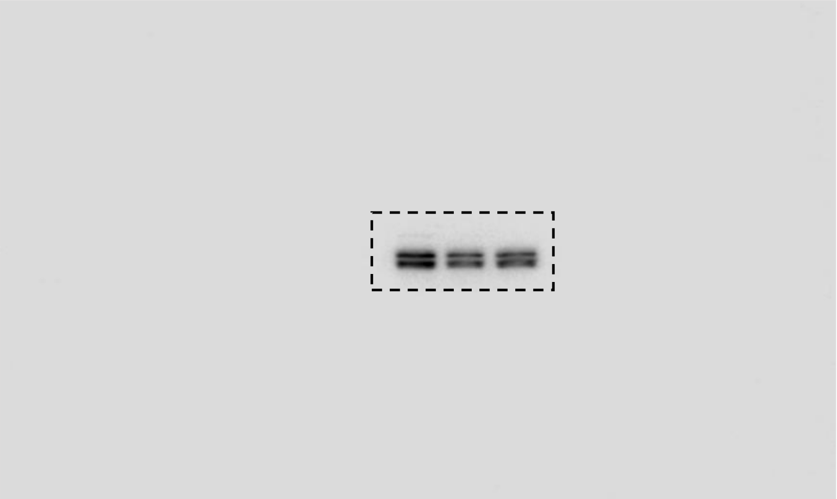

Supplemental material B.OCM1-shHDAC7

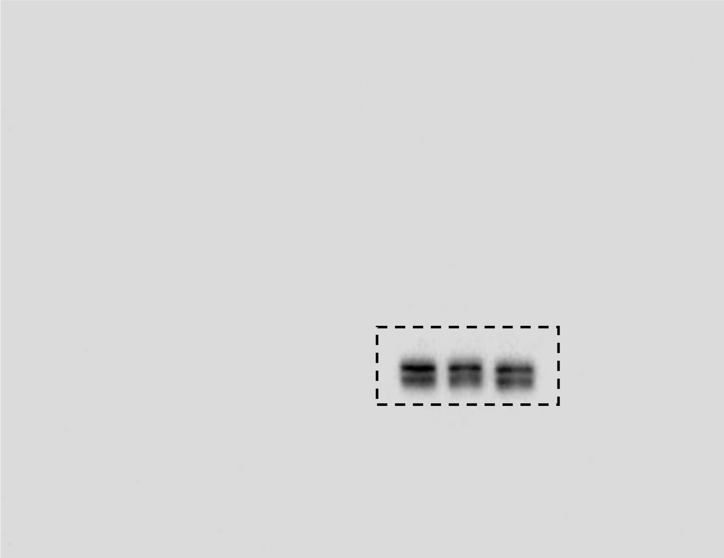

Supplemental material B.C918-shHD[illegible]

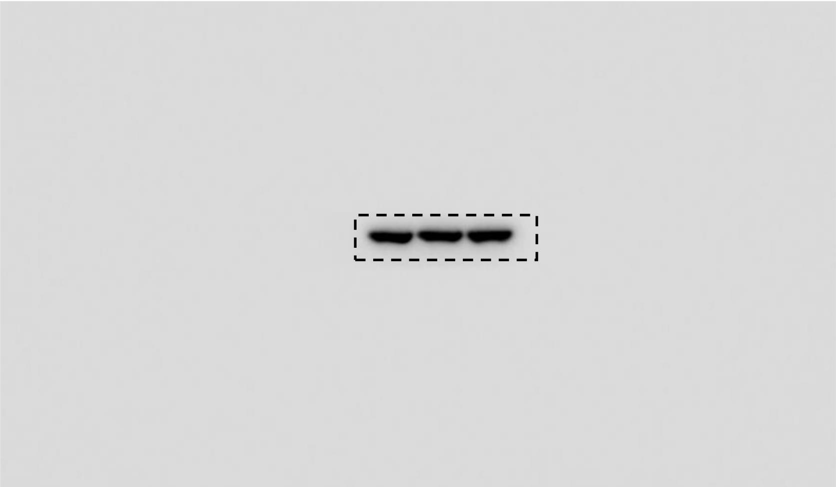

Supplemental Figure2 B.OCM1-β-actin

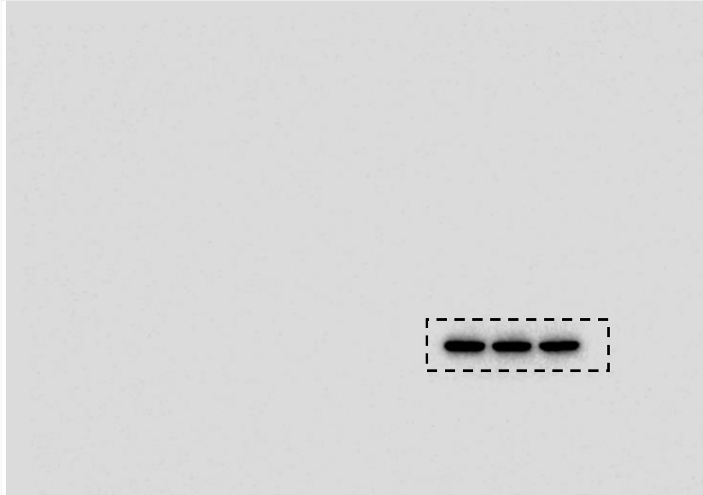

Supplemental Figure2 B.C918-[illegible]

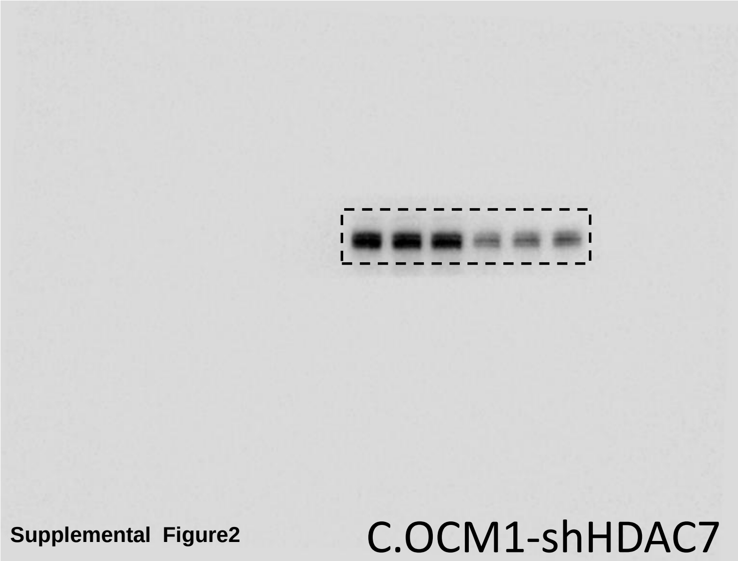
Supplemental Figure2
C.OCM1-shHDAC7

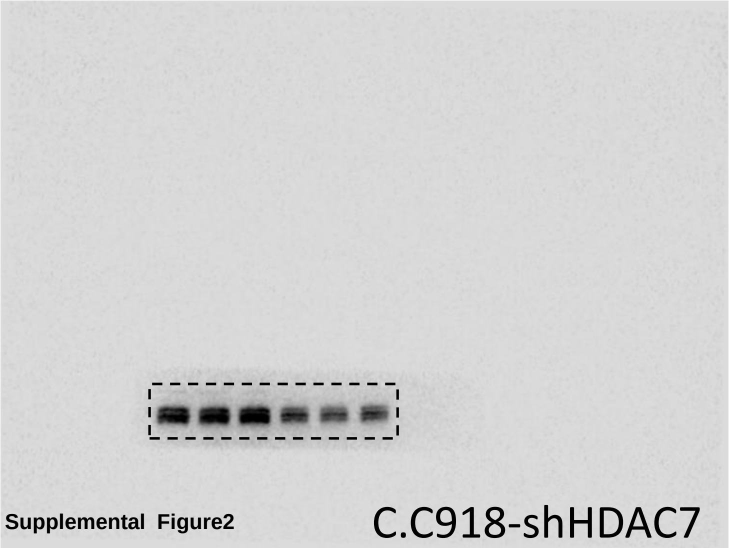
Supplemental Figure2
C.C918-shHDAC7

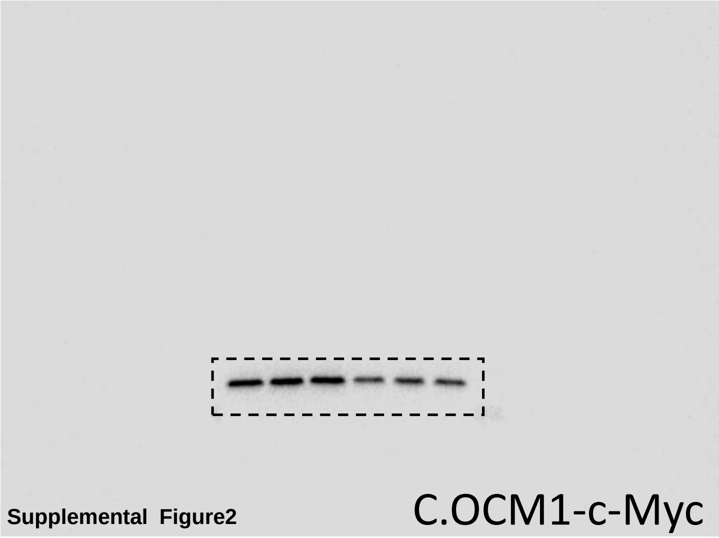
Supplemental Figure2
C.OCM1-c-Myc

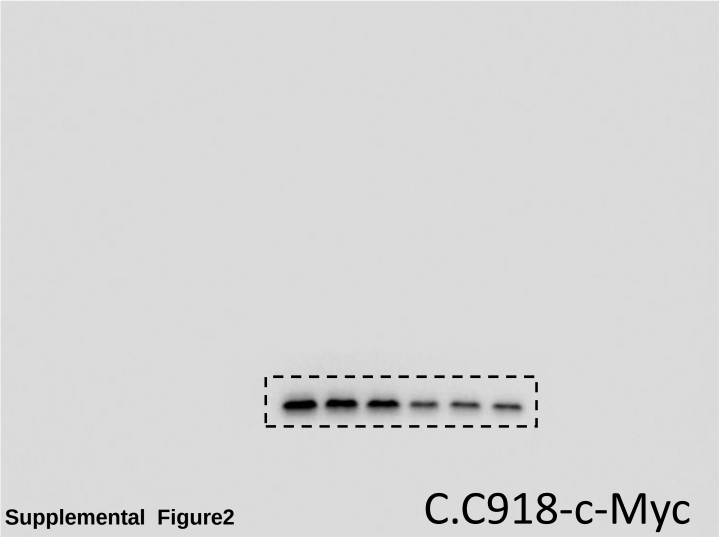
Supplemental Figure2
C.C918-c-Myc

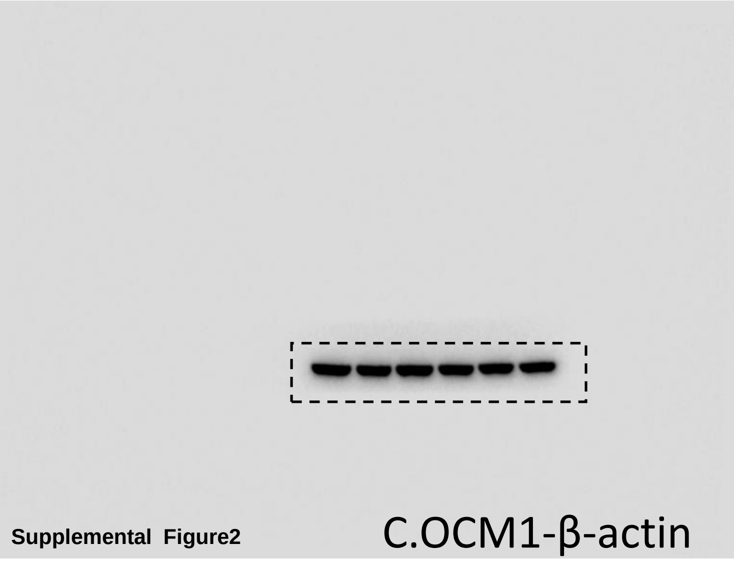
Supplemental Figure2
C.OCM1-β-actin

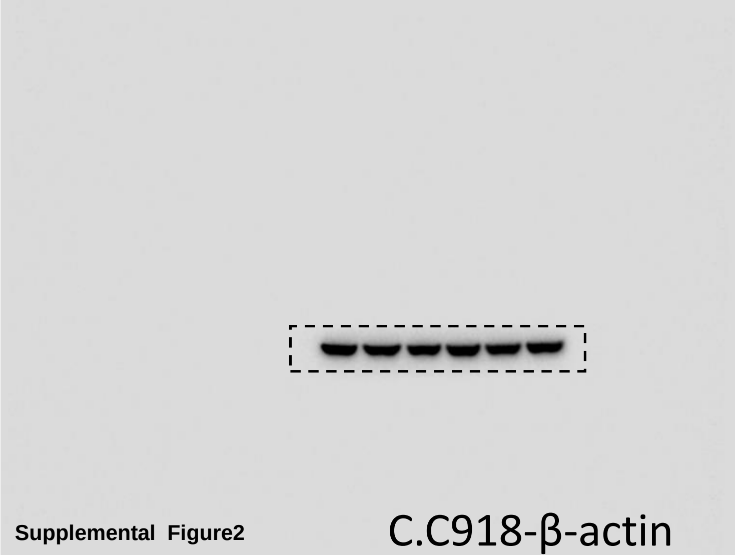
Supplemental Figure2
C.C918-β-actin
