# Supplementary material for: HDAC7/c-Myc signaling pathway promotes the proliferation and metastasis of choroidal melanoma cells
Source: Cell Death Dis. 2023 Jan 18;14(1):38. doi: 10.1038/s41419-022-05522-0 (PMC9849404; doi:10.1038/s41419-022-05522-0)

Fig1(A)/OCM1 Fig1(A)/C918

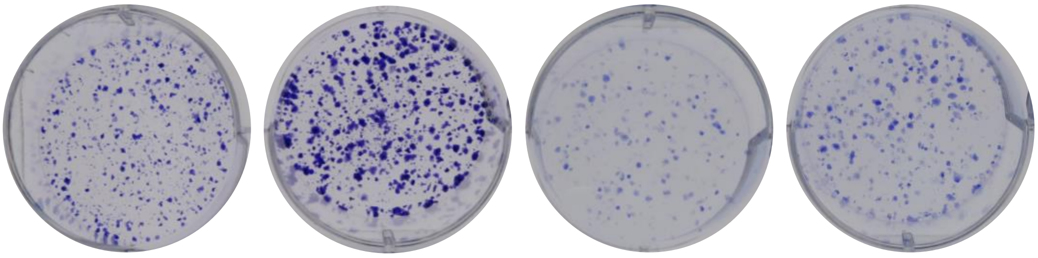

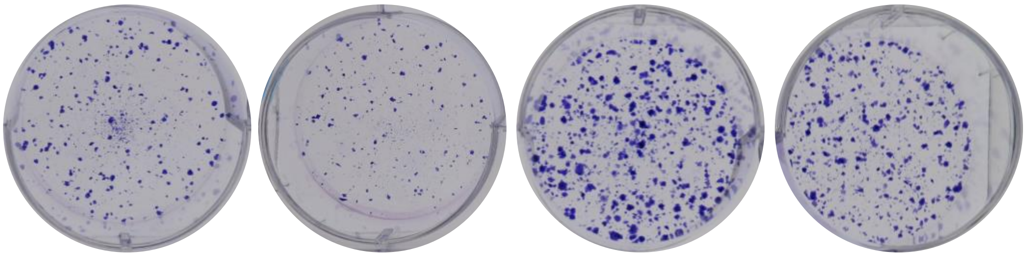

Fig2(A)/OCM1

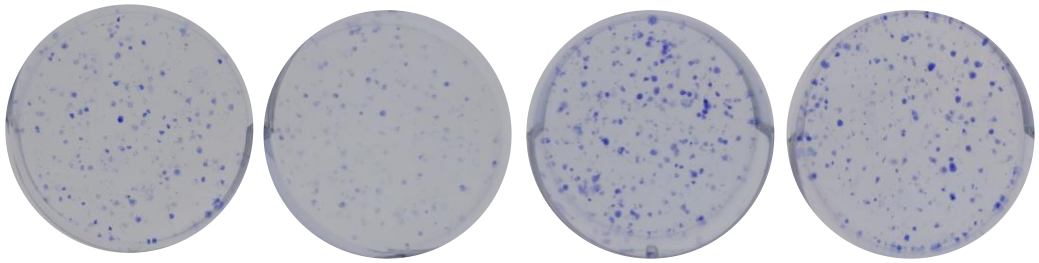

Fig2(A)/C918

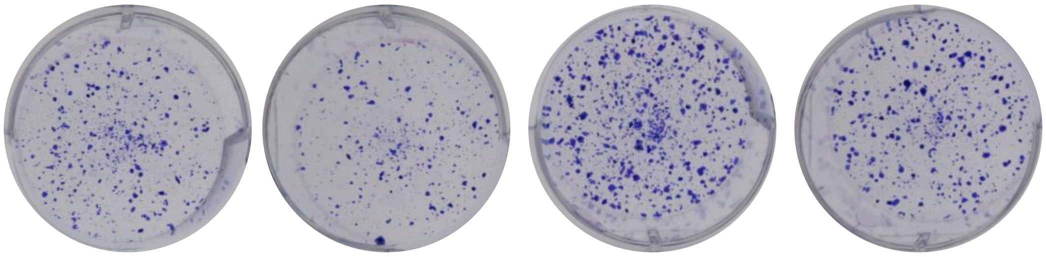

Fig3(A)/OCM1

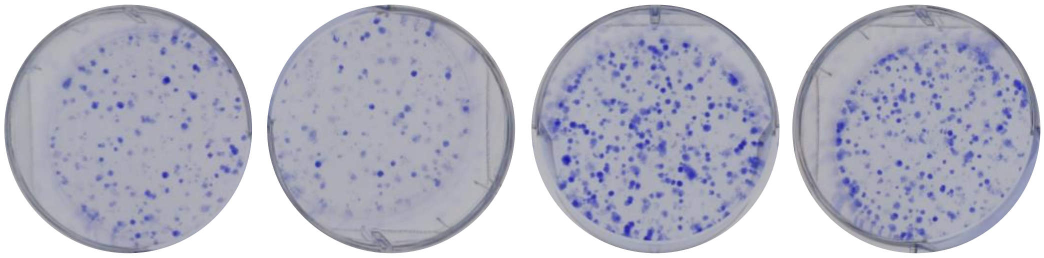

Fig3(A)/C918

Fig7(A)/OCM1 Fig7(A)/C918

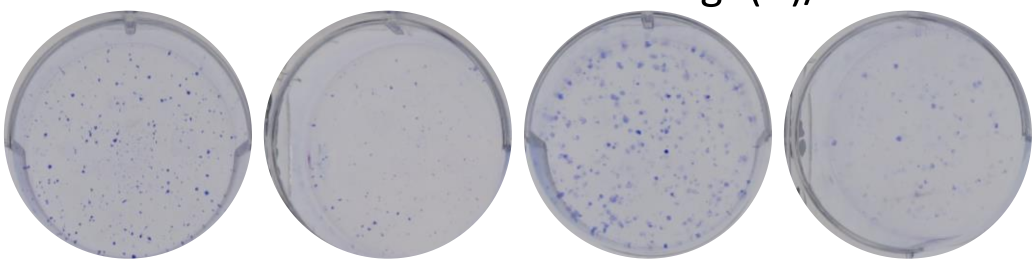

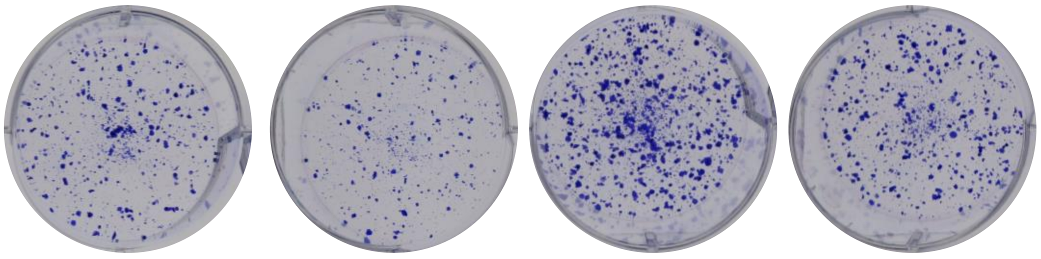

Fig8(A)/OCM1

Fig1(B)/OCM1

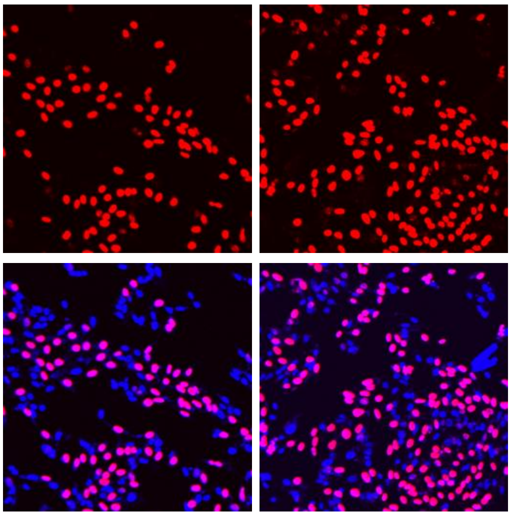

Fig1(B)/C918

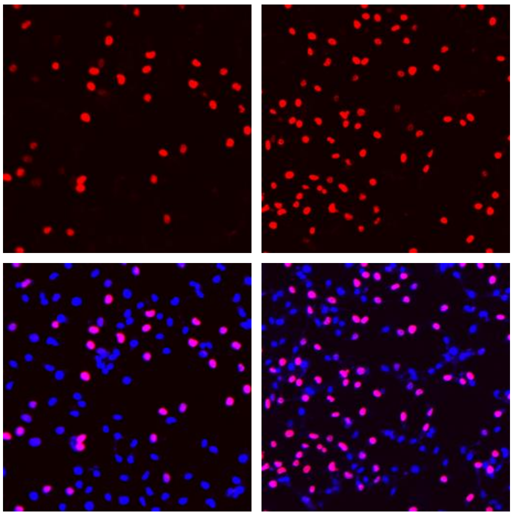

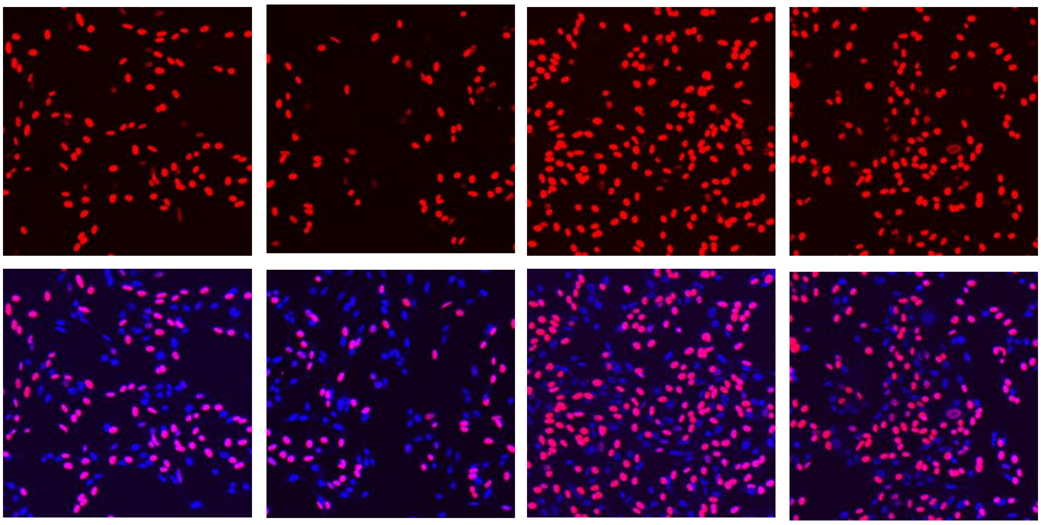

Fig2(B)/OCM1

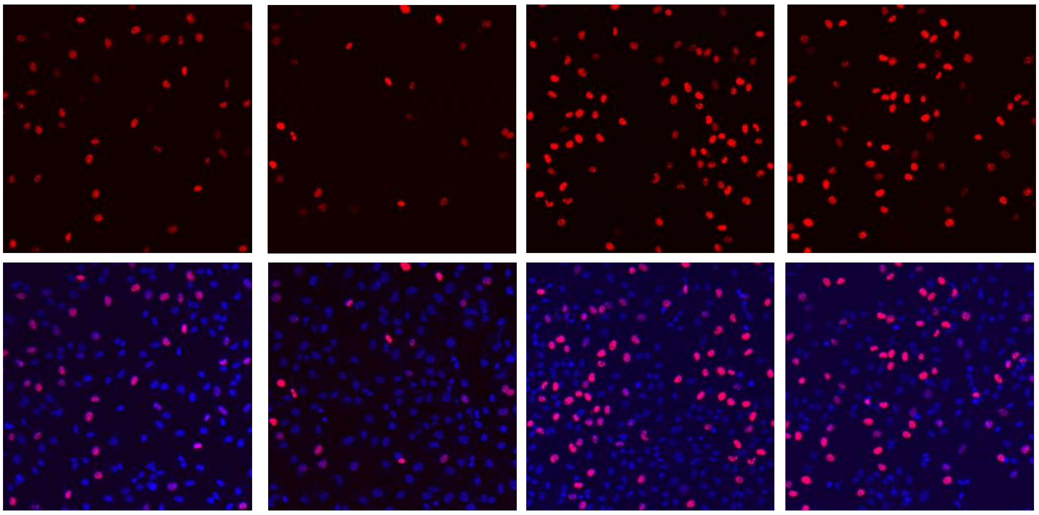

Fig2(B)/C918

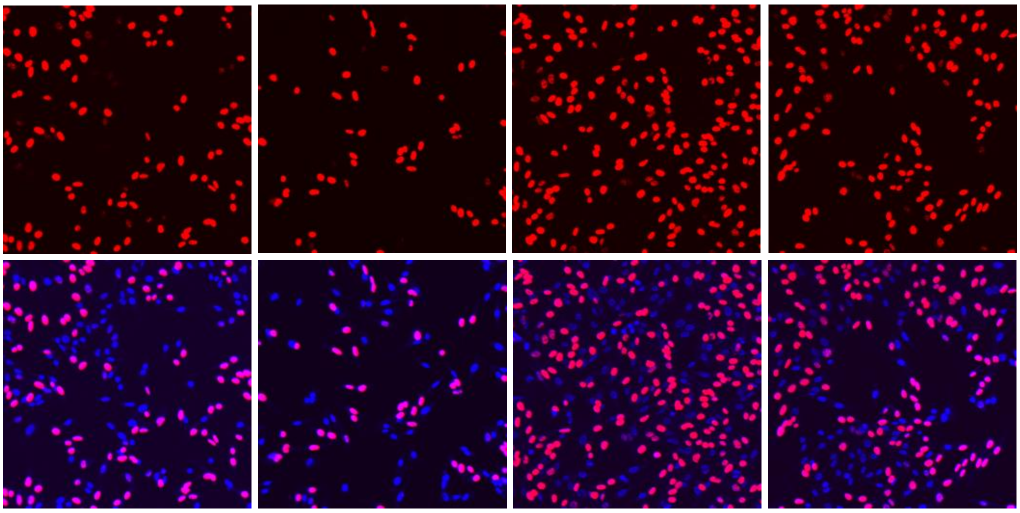

Fig3(B)/OCM1

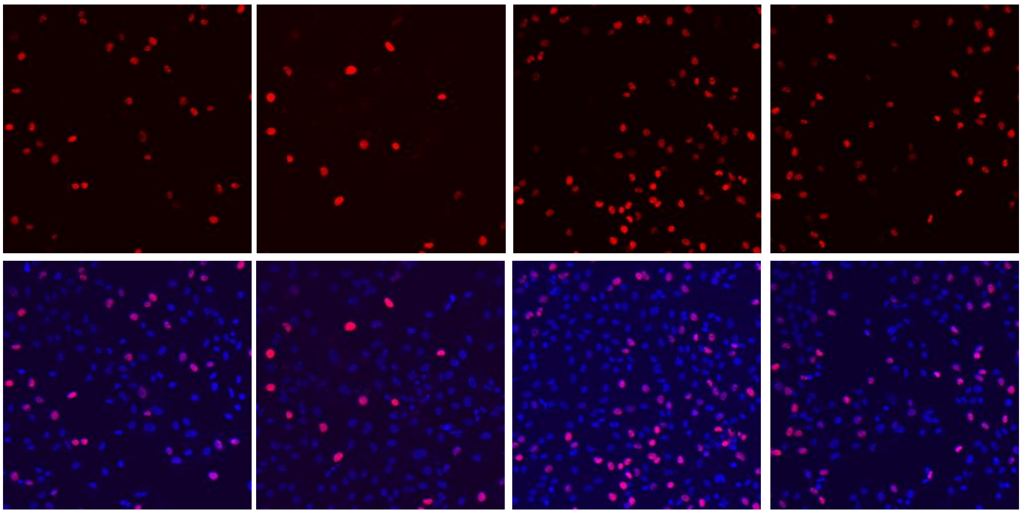

Fig3(B)/C918

Fig7(B)/ OCM1

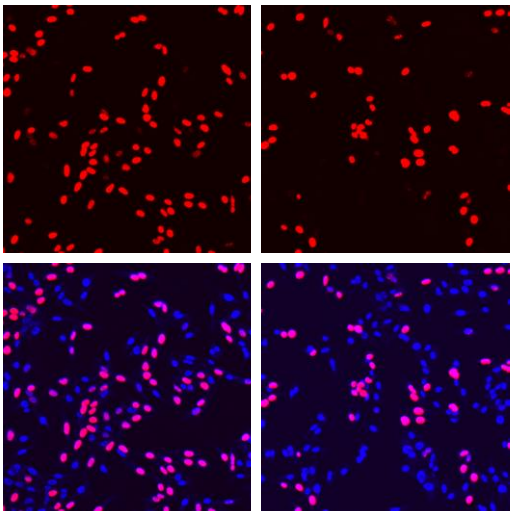

Fig7(B) /C918

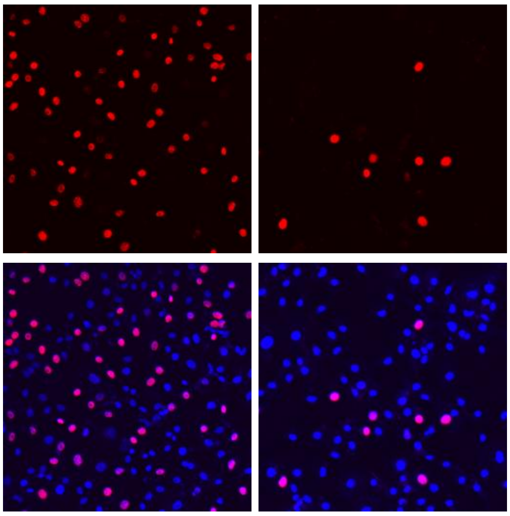

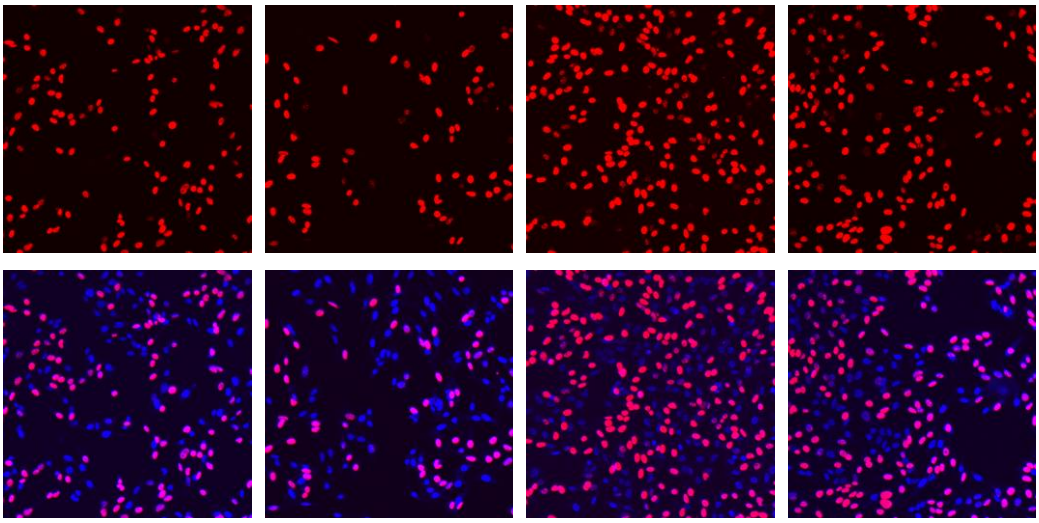

Fig8(B)/OCM1

Supplementary figure (A)

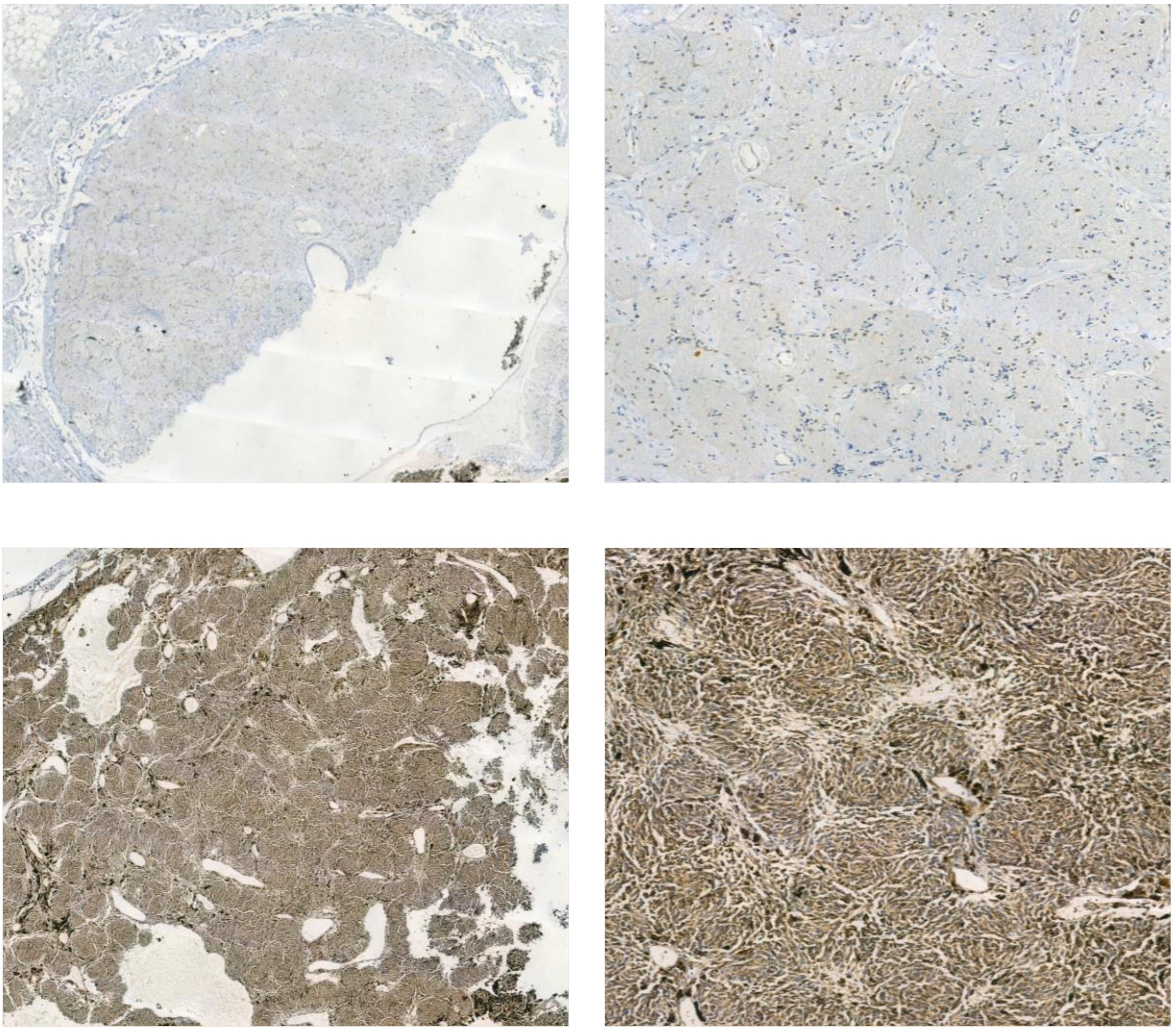

Fig4(B)/OCM1

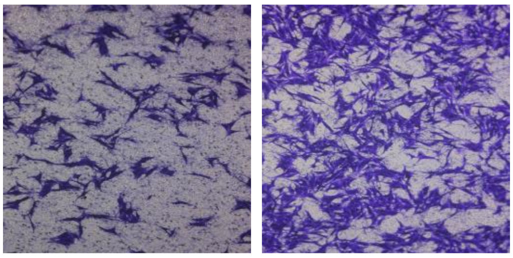

Fig4(B)/C918

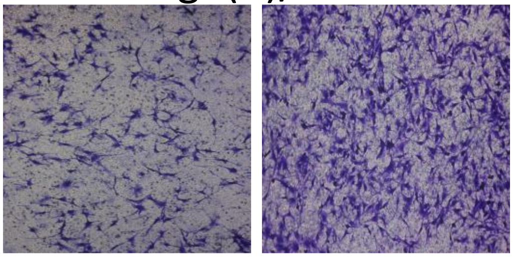

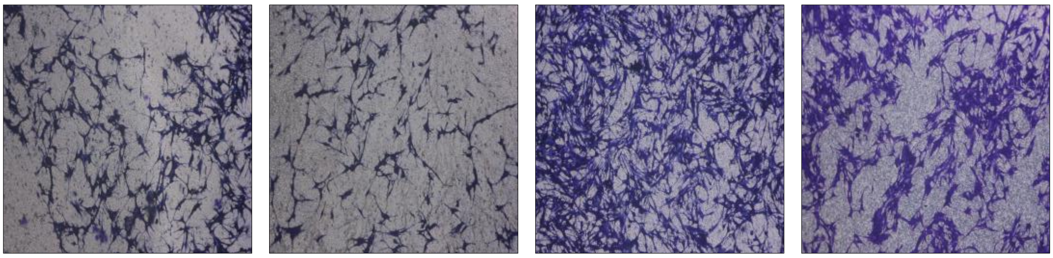

Fig5(B)/OCM1

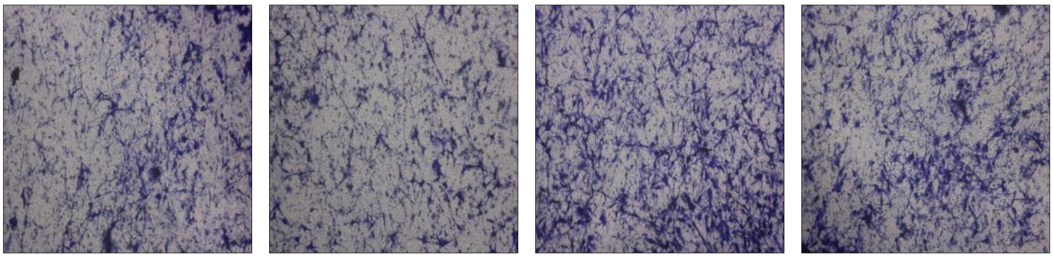

Fig5(B)/C918

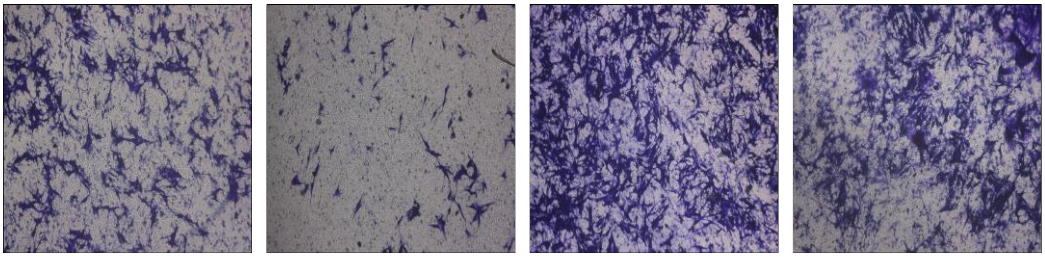

Fig6(B)/ OCM1

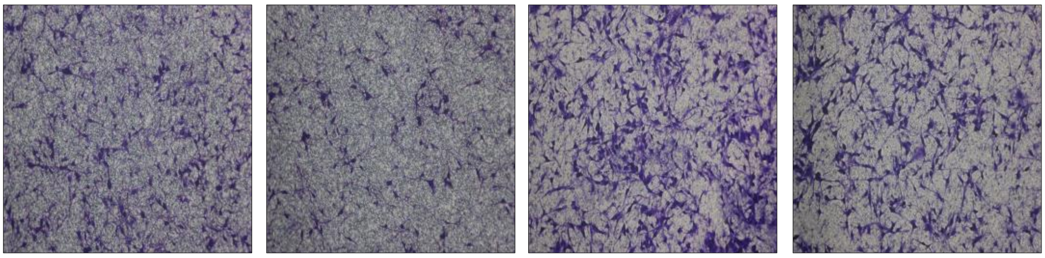

Fig6(B) /C918

Fig7(E)/OCM1

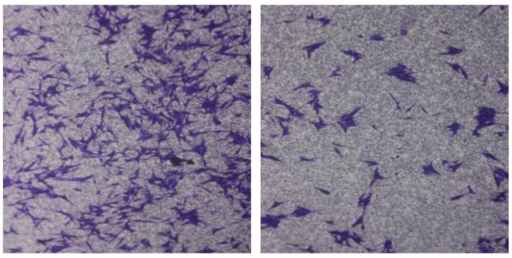

Fig7(E)/C918

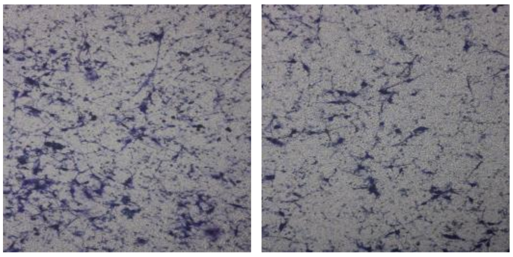

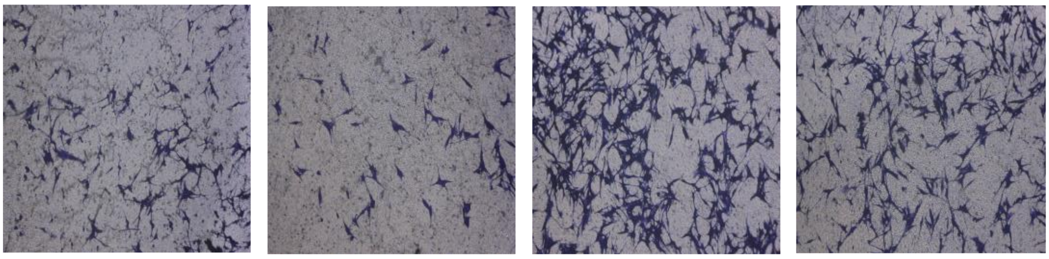

Fig8(E)/OCM1

Fig4(A)/OCM1

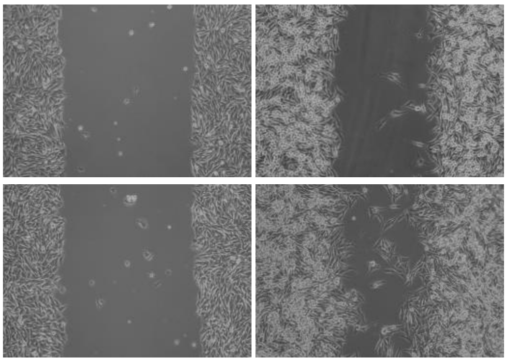

Fig4(A)/C918

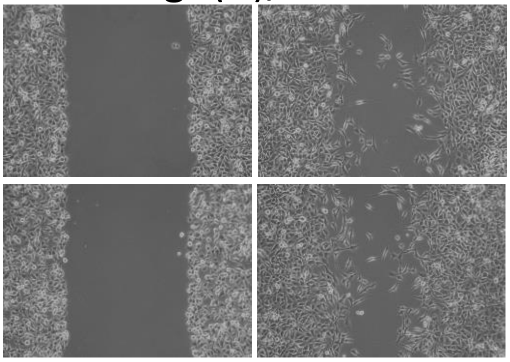

Fig5(A)/OCM1

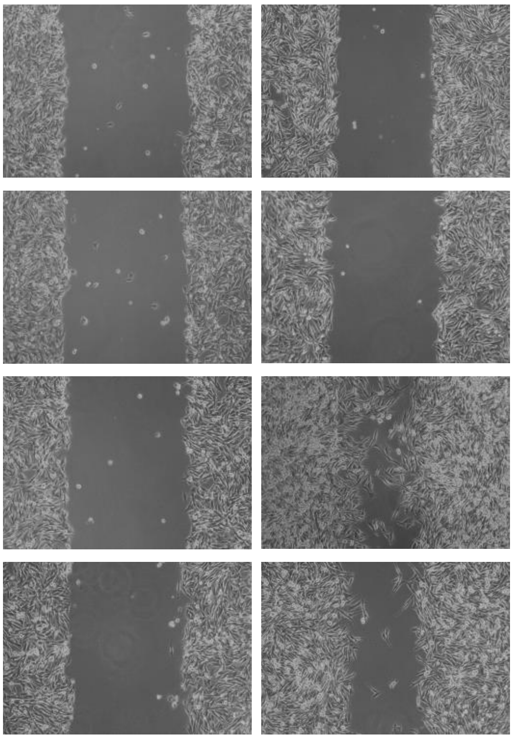

Fig5(A)/C918

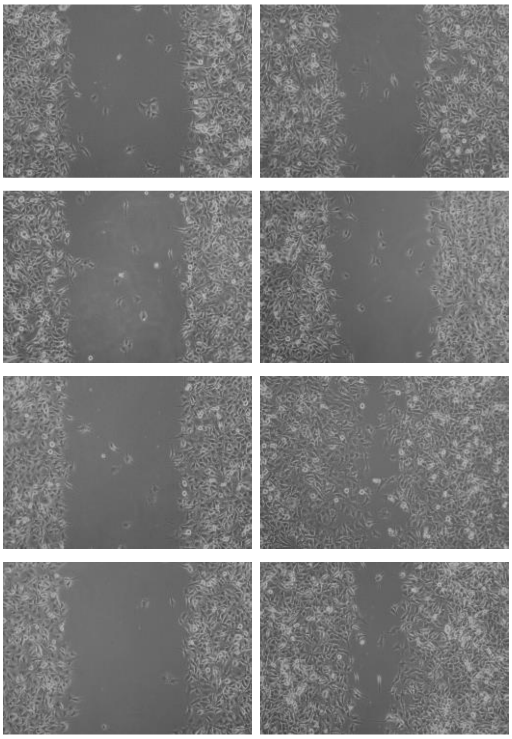

Fig6(A) /OCM1

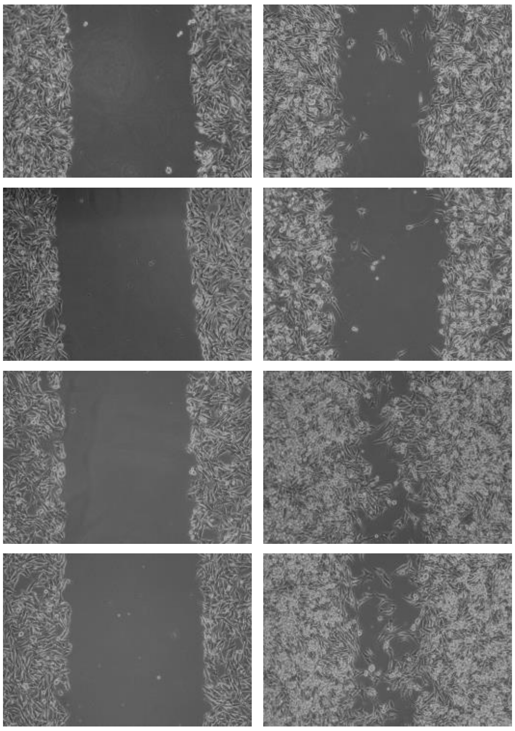

Fig6(A) C918

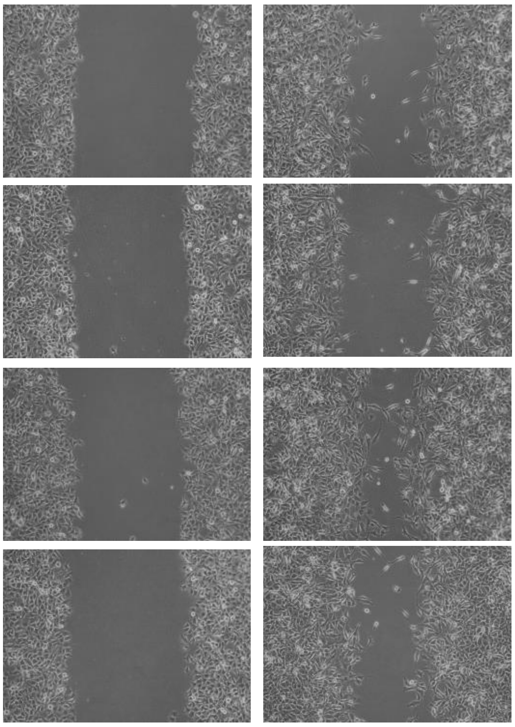

Fig7(D)/OCM1

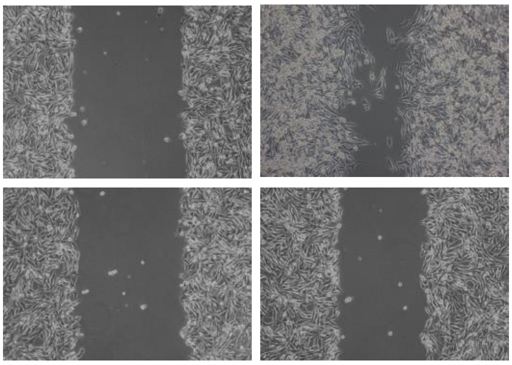

Fig7(D) /C918

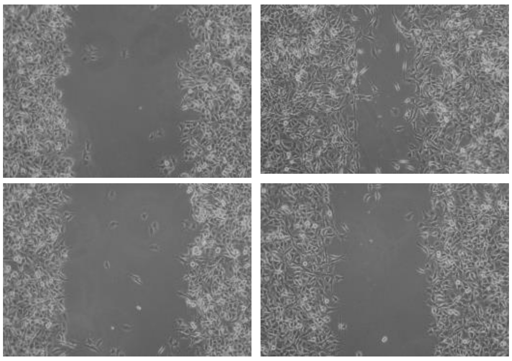

Fig8(D)/OCM1

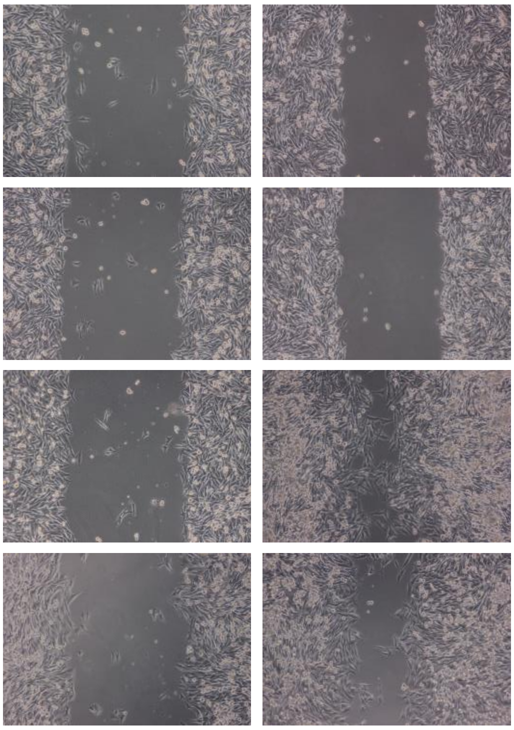

Supplement: Supplementary file 6 — Original Data File [file 41419_2022_5522_MOESM6_ESM.pdf]
